# Physical activity and oxidative stress biomarkers in humans: a systematic review and quantitative meta-analysis

**DOI:** 10.1186/s13102-026-02059-z

**Published:** 2026-09-18

**Authors:** Hassan Rahim Wahib, Roghayyeh Afroundeh, Reza Farzizadeh, Reza Malekzadeh

**Affiliations:** 1 Department of Sport Physiology, Faculty of Educational Science and Psychology, University of Mohaghegh Ardabili, Ardabil, Iran; 2 Department of Medical Physics, School of Medicine, Tabriz University of Medical Sciences, Tabriz, Iran

**Keywords:** Physical activity, Oxidative stress, Meta-analysis, MDA, GSH, Redox regulation

## Abstract

**Background:**

The effects of physical activity on oxidative stress biomarkers in humans remain heterogeneous. This systematic review and meta-analysis quantified exercise-induced changes in key redox biomarkers.

**Methods:**

Following PRISMA 2020 (PROSPERO: CRD420251144711), a systematic search of PubMed, Scopus, and Web of Science (2000–2025) identified human studies assessing malondialdehyde (MDA), superoxide dismutase (SOD), catalase (CAT), glutathione (GSH), total antioxidant capacity (TAC), and total antioxidant status (TAS). Random-effects models estimated pooled standardized mean differences (SMD) with 95% confidence intervals (CI). Subgroup analyses, meta-regression, sensitivity analyses, publication bias, and risk-of-bias assessments were fully performed.

**Results:**

Among 98 eligible studies (up to 31 meta-analyzed, *n =* 4,742), physical activity significantly reduced MDA (SMD = -1.02; 95% CI: -1.95 to -0.09; *p =* 0.032; I² = 94.6%) and increased TAS (SMD = 0.76; 95% CI: 0.38 to 1.15; *p <* 0.001; I² = 0.0%). GSH showed a non-significant trend toward reduction (SMD = -0.58; *p =* 0.075; I² = 88.5%), while CAT, SOD, and TAC demonstrated no significant pooled effects. Subgroup analyses identified effect modification by age, BMI, and duration, though meta-regression found no linear relationships with age or duration. Sensitivity analyses confirmed robustness for SOD, GSH, and TAS, while MDA, CAT, and TAC were influenced by influential studies. Publication bias was detected for GSH (Egger’s *p =* 0.006). Heterogeneity was substantial (I² = 87–97%), and most studies were of moderate methodological quality.

**Conclusion:**

Physical activity selectively reduces lipid peroxidation and enhances non-enzymatic antioxidant capacity without uniformly upregulating enzymatic defenses. However, due to substantial heterogeneity, moderate methodological quality, and potential publication bias for GSH, these findings should be interpreted cautiously as exploratory.

**Trial registration:**

*PROSPERO CRD420251144711*.

**Supplementary Information:**

The online version contains supplementary material available at https://doi.org/10.1186/s13102-026-02059-z.

## Introduction

Physical activity ranks among the most potent and accessible interventions for preventing non-communicable diseases, reducing global mortality by 8–13% according to WHO estimates [1, 2]. Regular physical activity is widely recognized as a cornerstone of health promotion, contributing to reduced risk of cardiovascular disease, metabolic disorders, certain cancers, and premature mortality [3, 4]. Beyond its well-established effects on body composition, insulin sensitivity, and cardiorespiratory fitness, physical activity also influences cellular redox homeostasis, a dynamic balance between pro-oxidant and antioxidant processes that is essential for maintaining physiological function [5].

In recent years, growing attention has been directed toward the relationship between exercise and oxidative stress biomarkers, because this pathway may help explain both the beneficial adaptations and the potential adverse effects associated with different levels, intensities, and modes of physical activity [6]. Oxidative stress occurs when the generation of reactive oxygen and nitrogen species exceeds the capacity of endogenous antioxidant defenses, resulting in oxidative damage to lipids, proteins, and nucleic acids [7, 8]. Common biomarkers used to assess this process include malondialdehyde (MDA), total antioxidant capacity (TAC), superoxide dismutase (SOD), glutathione peroxidase (GPx), catalase (CAT), and related indices of oxidative injury or antioxidant status [9, 10]. These biomarkers are clinically and biologically important because they provide measurable insight into systemic redox balance and may reflect long-term health risk or adaptation to physiological stress [11, 12]. 

However, the interpretation of these markers in relation to physical activity remains complex, as exercise can both transiently increase reactive species production and stimulate adaptive upregulation of antioxidant defenses [13]. The extent to which physical activity modulates oxidative stress appears to depend on multiple factors, including intensity, duration, frequency, training status, age, sex, body composition, and health condition [14]. Moderate habitual activity may enhance antioxidant capacity and improve oxidative resilience, whereas unaccustomed or prolonged high-intensity exertion may acutely elevate oxidative damage markers [15]. This dual effect has led to inconsistent findings across human studies, with some reporting reductions in oxidative stress biomarkers after regular activity and others showing null or mixed associations [15].

Differences in biomarker selection, study design, sample characteristics, and exercise protocols further contribute to this heterogeneity, making it difficult to draw firm conclusions from individual investigations [16]. Early studies, such as those by Mănescu et al. [17], Zeng et al. [16] and Alessio et al. [18], established exercise as a potent modulator of ROS, showing acute bouts elevate MDA and F2-isoprostanes while chronically upregulating SOD and GPx in trained athletes. Several reviews highlighted the “exercise paradox”: high-intensity efforts spike oxidative damage transiently, yet regular moderate activity bolsters mitochondrial efficiency and Nrf2-mediated antioxidant gene expression, mitigating long-term stress [19, 20].

### Rationale and objectives

The redox‑modulating effects of physical activity remain incompletely understood, with conflicting evidence regarding whether exercise acts as a broad‑spectrum antioxidant enhancer or a selective redox modulator. This systematic review and meta‑analysis is the first to quantitatively synthesize the effects of physical activity on six major redox biomarkers, MDA, SOD, CAT, GSH, TAC, and TAS, within a unified framework. This approach provides the most comprehensive quantitative evidence to date on exercise‑induced redox changes, identifies sources of heterogeneity, and determines which biomarkers are most responsive to physical activity. This evidence is relevant for understanding biological mechanisms and informing precision exercise recommendations. The main objectives are:

- To quantify pooled effects of physical activity on oxidative damage (MDA) and antioxidant biomarkers (SOD, CAT, GSH, TAC, TAS).
- To compare effects on enzymatic (SOD, CAT) versus non‑enzymatic (TAS, TAC) antioxidants and glutathione status (GSH).
- To identify moderators (age, BMI, duration) of exercise‑redox relationships.

## Methodology

### Rationale for combining heterogeneous evidence

We acknowledge that our broad inclusion criteria, spanning diverse populations, exercise modalities, and study designs, would inevitably introduce substantial heterogeneity. However, this diversity was intentional and pre-specified in our PROSPERO-registered protocol. Our primary aim was to provide the first comprehensive quantitative synthesis of physical activity effects on six major redox biomarkers across the full spectrum of human conditions, enabling the identification of key effect modifiers (age, BMI, duration) that would be undetectable in a restricted meta-analysis. To manage this anticipated heterogeneity, we implemented a pre-specified analytical hierarchy: random-effects models (REML) with Hartung–Knapp adjustment, prediction intervals, subgroup analyses, meta-regression, and complementary sensitivity and publication bias assessments. This multi-layered approach ensures that our conclusions are drawn at the appropriate level of evidence, with greater weight placed on subgroup-specific findings where heterogeneity is substantially reduced.

### Study design and protocol registration

This systematic review and meta-analysis was conducted in accordance with the Preferred Reporting Items for Systematic Reviews and Meta-Analyses (PRISMA) 2020 statement and its corresponding methodological recommendations [21]. The protocol of the study was prospectively registered in the PROSPERO international prospective register of systematic reviews (registration ID: CRD420251144711). The review protocol predefined the research objectives, eligibility criteria, information sources, search strategy, study selection process, data extraction procedures, and statistical methods used for the meta-analysis. The reporting of the study followed the PRISMA 2020 checklist to ensure transparency and reproducibility. The completed PRISMA checklist is provided in Table S1.

The research question was developed using the PICOs (Population, Intervention, Comparator, Outcomes, and Study design) framework to define the eligibility criteria. The population included human participants of any age or sex. The exposure of interest was physical activity or exercise interventions. Comparator groups included sedentary controls or baseline measurements when applicable. The primary outcomes were biomarkers of oxidative stress and antioxidant status measured in human biological samples (blood, plasma, serum). Eligible study designs included randomized controlled trials (RCTs), controlled clinical, and cross-section, and observational studies reporting relevant quantitative data. Detailed eligibility criteria based on the PICOS framework are presented in Table S2.

### Eligibility

#### Inclusion criteria

Studies were considered eligible for inclusion if they met all of the following criteria: (1) they were original, peer-reviewed quantitative research articles; (2) they investigated the effects of physical activity or structured exercise interventions, including acute single bouts or chronic training programs (≥ 2 weeks) such as aerobic/endurance training, resistance training, high-intensity interval training (HIIT), sprint interval exercise (SIE), combined aerobic-resistance programs, or sport-specific exercise performed in laboratory or field settings, this broad inclusion was intentional to capture the full spectrum of redox responses across diverse modalities, populations, and contexts; (3) they included therapeutic physical maneuvers (namely, analith repositioning for benign paroxysmal positional vertigo) as ‘exercise-like’ interventions that induce systemic physiological stress through head and body positioning, provided they reported relevant redox biomarkers, with sensitivity analyses pre-planned to exclude these non-conventional interventions and subgroup analyses/meta-regression employed to account for this diversity; (4) they reported at least one biomarker of oxidative stress or antioxidant defense measured in human biological samples (blood, plasma, or serum), including oxidative damage markers (namely, MDA, lipid peroxidation products) and antioxidant indicators such as SOD, CAT, GPx, glutathione reductase (GR), reduced glutathione (GSH), oxidized glutathione (GSSG), or total antioxidant capacity (TAC) assessed by FRAP, ORAC, or similar assays; (5) they provided quantitative pre- and/or post-exercise data or sufficient statistical information to calculate effect sizes; and (6) they reported adequate details regarding exercise characteristics, including intensity, duration, frequency, or workload.

#### Exclusion criteria

Studies were excluded if they met any of the following criteria: (1) absence of a physical activity or exercise stimulus (namely, studies examining purely pharmacological, nutritional, or other interventions without a concurrent exercise component); (2) lack of direct assessment of oxidative stress or antioxidant biomarkers; (3) focus exclusively on training or performance outcomes without examining oxidative stress or redox-related mechanisms; (4) insufficient reporting of exercise characteristics (intensity, duration, frequency, or workload), preventing interpretation of exercise-related effects; (5) publication types without original empirical data, including narrative reviews, systematic reviews, meta-analyses, perspectives, editorials, commentaries, and letters; (6) study protocols or methodological papers without reported outcome data; (7) unclear, incomplete, or poorly described methodology regarding exercise prescription or biomarker measurement; and (8) studies not directly related to exercise-induced oxidative stress or redox biology in humans.

### Information sources and search strategy

The search strategy for this systematic review and meta-analysis was developed a priori to comprehensively identify studies investigating the effects of physical activity and exercise on oxidative stress and redox-related biomarkers in humans. A systematic search was conducted in three major electronic databases namely, PubMed, Scopus, and Web of Science from January 2000 to November 2025 by two independent reviewers (*R. F. and R. M.*).

The search strategy combined free-text keywords and, where applicable, Medical Subject Headings (MeSH) related to physical activity and oxidative stress biomarkers. The core search concepts included: (i) physical activity or exercise (exercise, physical activity, training, aerobic training, resistance training, treadmill exercise); (ii) oxidative stress and redox biology; and (iii) specific oxidative stress and antioxidant biomarkers (MDA, SOD, CAT, GPx, GSH, GSSG, and TAC). These terms were combined using Boolean operators (“AND”, “OR” and “NOT”) and truncation where appropriate to maximize search sensitivity while maintaining specificity for exercise-related oxidative stress outcomes. The search was limited to peer-reviewed articles published in English. In addition to database searching, backward and forward citation tracking was conducted. The reference lists of all included studies and relevant review articles were manually screened, and articles citing the included studies were also examined to identify additional eligible publications. The complete search syntax for each database and the number of retrieved records are presented in Table S3.

### Study selection and screening process

**Fig. 1:**
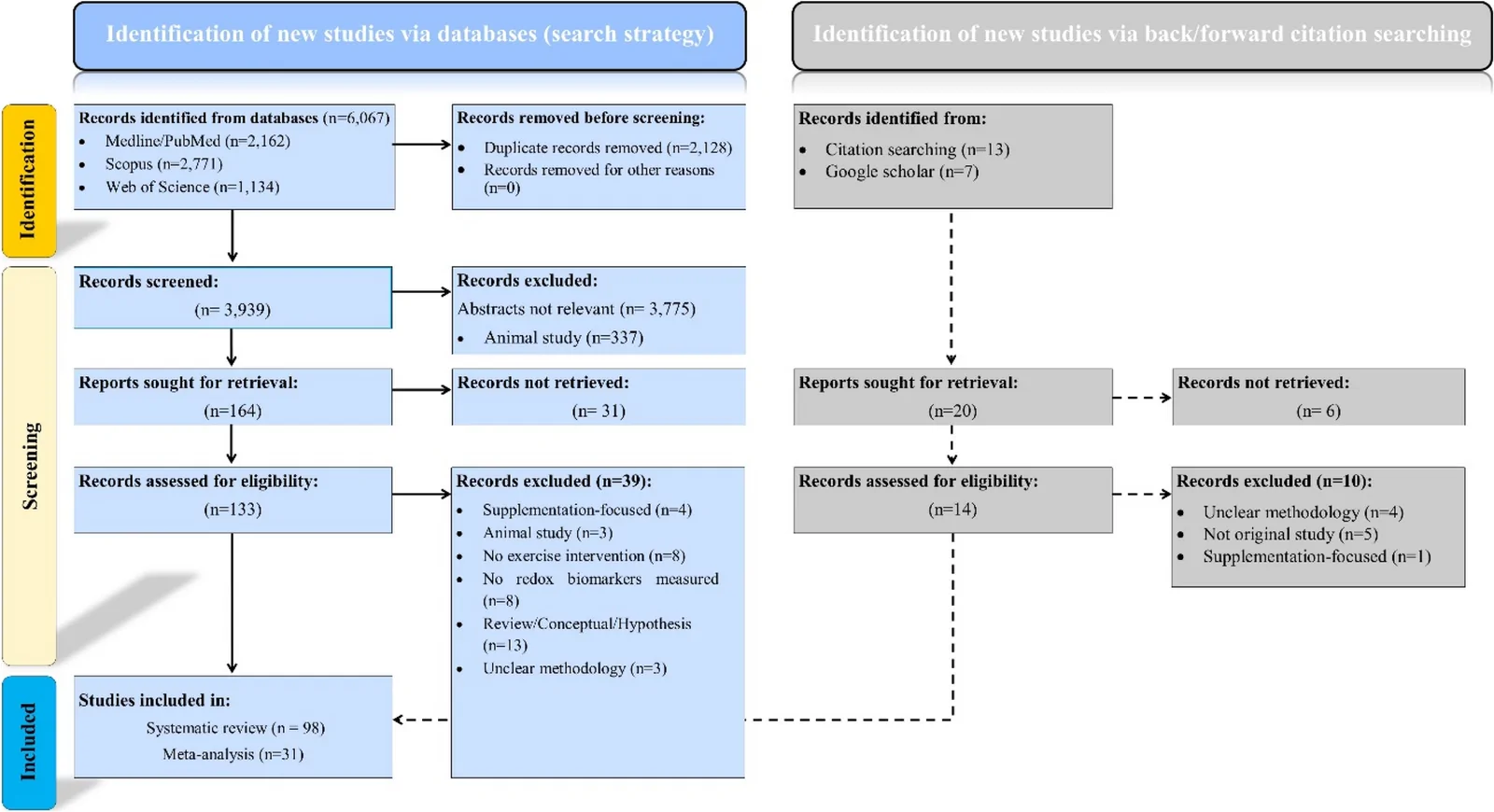
PRISMA flow diagram illustrating the study selection process, including identification, screening, eligibility assessment, and final inclusion of studies

All records retrieved from the database searches were imported into EndNote reference management software (version 2025.3.1; v19926), and duplicate entries were removed. The study selection process was conducted in two sequential stages in accordance with PRISMA guidelines. First, titles and abstracts were screened independently by two reviewers (*R. F. and R. M.*) based on the predefined inclusion and exclusion criteria. Studies that were clearly irrelevant, such as those lacking an exercise or physical activity component, involving non-human populations, or not reporting oxidative stress or antioxidant biomarkers were excluded at this stage. Second, the full texts of all potentially eligible studies were retrieved and assessed independently by the same reviewers for final inclusion. Any disagreements during the screening or full-text evaluation stages were resolved through discussion and consensus. When necessary, a third reviewer was consulted to resolve unresolved discrepancies. Reasons for exclusion at the full‑text stage (wrong population, absence of structured exercise intervention, lack of oxidative stress biomarker assessment, or insufficient reporting of exercise characteristics) were recorded and are summarized in Table S4. The overall process of study identification, screening, eligibility assessment, and final inclusion is illustrated in the PRISMA flow diagram (Fig. 1).

### Data extraction

After finalizing the eligible studies through the two-stage screening process (title/abstract screening and full-text review), data extraction was performed independently by two reviewers (*R. F. and R. M.*) using a pre-piloted and standardized data extraction form. Extracted data were cross-checked to ensure accuracy and completeness.

For each included study, the following information was recorded: (1) study characteristics, including first author, year of publication, country, and study design; (2) participant characteristics, including sample size, age, sex, health or disease status, and key inclusion or exclusion criteria, as well as baseline oxidative stress biomarker profiles where available; (3) intervention or exposure characteristics, including exercise modality (aerobic, resistance, HIIT), exercise intensity, session duration, training frequency, and total intervention duration; (4) outcome measures, including oxidative stress and antioxidant biomarkers such as GSH, GSSG, SOD, CAT, GPx, MDA and TAC, as well as the timing of biological sample collection relative to exercise; (5) for each outcome, we extracted the sample size, mean, and standard deviation for both intervention and control groups at post-intervention, and where available, pre-intervention values and change scores; (6) for crossover and single-group studies, we extracted within-group change data and recorded whether the study reported change score standard deviations or provided sufficient data for imputation; and (5) additional study notes, including reported study limitations, potential sources of bias, or methodological considerations relevant to the interpretation of results.

Any discrepancies during data extraction were resolved through discussion and consensus between the reviewers. In cases of missing or unclear information, the corresponding authors of the original studies were contacted when feasible.

### Geographic distribution and trend analysis

To visualize the geographic distribution of the included studies, the diagram was generated using SCImago Graphica (beta 1.0.55) based on the country of origin [22]. Additionally, to map the scientific landscape and research trends of the effect of exercise on cancer, a keyword co-occurrence network analysis was performed on 2,162 articles from PubMed. 240 keywords were entered into the network with at least 20 repetitions and normalized using VOSviewer (version 1.6.21.) software and the association strength method. Nodes were colored based on the average year of publication, with a blue (old) to yellow (new) spectrum, showing the temporal evolution of the themes. In the network, node size represents keyword frequency [23].

### Risk of bias (RoB) assessment

Methodological quality was assessed using design‑specific tools. For the RCTs, the Cochrane Risk of Bias tool (RoB 2) was applied across five domains [24]. For the non‑randomized intervention studies (including acute pre‑post designs), the ROBINS‑I (Risk Of Bias In Non‑randomized Studies – of Interventions) tool was used to evaluate confounding, selection, and measurement biases [25]. For the remaining observational and cross‑sectional studies, the Newcastle‑Ottawa Scale (NOS) was employed to assess selection, comparability, and outcome domains [26]. To visualize the risk‑of‑bias assessments, we used the NOS‑TLPlot Python tool, which is specifically designed for graphical presentation of NOS results [27]. Additionally, the Risk of Bias Visualization (robvis) tool was utilized to display the results of the RoB 2 and ROBINS‑I assessments [28]. All evaluations were performed independently by two reviewers (*R. F. and R. M.*), with disagreements resolved through consensus. Detailed results of these assessments for each included study are presented in Figs. S4–S6 and summarized in the main text.

### Formal analysis

#### Meta-analysis

A quantitative meta-analysis was conducted to estimate the overall effects of physical activity and exercise on oxidative stress and antioxidant biomarkers in humans. Statistical analyses were performed using Comprehensive Meta-Analysis (CMA) software (version 4) to generate pooled effect estimates, forest plots, funnel plots, and subgroup analyses. Random-effects models were applied using the restricted maximum likelihood (REML) estimator, and the Hartung–Knapp adjustment was used to provide more robust CIs [29]. Effect sizes were calculated as standardized mean differences (SMD) with 95% confidence intervals (95% CI) for continuous outcomes, including SOD, GPx, CAT, GSH, TAC, and MDA.

To calculate effect sizes, we employed the SMD approach. For parallel-group designs (intervention vs. control), effect sizes were computed using post-intervention means and pooled standard deviations for each group. For pre-post within-group designs (single-group or crossover studies), we used change scores (post-intervention minus pre-intervention), calculating SMD as the difference in change scores between intervention and control conditions divided by the pooled standard deviation of change scores. Studies were included in the meta-analysis only if they clearly reported a structured exercise intervention alongside a suitable comparator group (either a parallel control group or a pre-post within-group design with extractable baseline and post-intervention data) [30]. For each included study, we required sample size, mean, and standard deviation for both the intervention and control conditions. Studies that reported outcomes as medians without dispersion measures that could not be converted to means and standard deviations were excluded from the quantitative synthesis. In studies with multiple intervention arms (namely, different exercise intensities or modalities) sharing a single control group, we maintained the control group and entered each intervention arm as a separate comparison, using appropriate statistical corrections to avoid unit-of-analysis errors. For each included study, we entered the sample size, mean, and standard deviation for both intervention and control groups into the meta-analysis software (CMA, version 4), and effect sizes were automatically computed using the SMD formula. Studies were excluded from the quantitative synthesis if they: (i) lacked a suitable control or comparator group; (ii) reported outcomes as medians without means and SDs; (iii) provided insufficient statistical information; (iv) involved non-structured interventions such as supplementation-only protocols, therapeutic maneuvers, or purely observational seasonal exposures without a controlled exercise component; or (v) were cross-sectional or parallel-group studies without a true control condition.

When numerical data were presented only in graphical format, values were extracted using GetData Graph Digitizer (version 2.26). Studies were excluded from the quantitative synthesis if they lacked a suitable comparator group, reported only single-arm outcomes without extractable variance measures, presented results solely as medians without sufficient dispersion measures, or provided data that could not be reliably extracted for meta-analysis.

#### Heterogeneity assessment

Between-study heterogeneity was evaluated to determine the extent of variability in effect sizes across the included studies beyond that expected by chance. Statistical heterogeneity was assessed using Cochran’s Q statistic, the inconsistency index (I²), and the between-study variance (Tau²) [31]. Cochran’s Q test was used to determine whether the dispersion of study-specific effect sizes exceeded that expected from sampling error alone, with *p <* 0.05 indicating potential heterogeneity. Because the Q test is sensitive to the number of studies, it was interpreted alongside additional indices. The magnitude of heterogeneity was quantified using the I² statistic, representing the proportion of total variability attributable to true differences between studies rather than random error. I² values of 25%, 50%, and 75% were interpreted as low, moderate, and high heterogeneity, respectively. Tau² (τ²), estimated using the restricted maximum likelihood method within the random-effects model, provided an estimate of the absolute between-study variance.

#### Publication bias assessment and sensitivity analysis

Potential publication bias and small-study effects were assessed using funnel plots and statistical tests. Funnel plot asymmetry was evaluated visually and using Egger’s regression test, with *p <* 0.05 indicating potential bias [32]. Begg’s rank correlation test was applied as a complementary non-parametric method. The fail-safe N was calculated to estimate the influence of potential missing studies. To accurately assess the influence of individual studies on the pooled effect sizes, we performed a leave-one-out sensitivity analysis using Python 3 software with the following libraries: Pandas, Matplotlib, Seaborn, and custom data processing modules. This approach was chosen over the built-in sensitivity functions of CMA software, as Python enabled the generation of more detailed graphical outputs for sensitivity testing. In this analysis, each study was sequentially removed, and the pooled effect size was recalculated for the remaining studies. Studies whose deletion changed the main effect size by more than 0.3 were identified as potentially influential and were visually highlighted in red in the corresponding graphs (labeled as “LOO influential study”) [33].

#### Meta-regression analysis

Meta-regression analyses were conducted within a random-effects framework using the REML estimator to identify potential sources of heterogeneity. Univariate models examined the influence of mean participant age on effect sizes for CAT, GSH, MDA, SOD, and TAC. A significance threshold of *p <* 0.10 was considered exploratory evidence of effect modification, with results interpreted cautiously to generate hypotheses rather than establish definitive moderator effects [34].

#### Subgroup analysis

Subgroup analyses were conducted to investigate potential sources of heterogeneity. Studies were stratified by intervention duration (≤ 6 vs. > 6 weeks), mean age (≤ 30 vs. > 30 years), and BMI category (≤ 25 vs. > 25). Pooled effect sizes were estimated using a random-effects model, and subgroup differences were evaluated using the Q_between statistic (*p <* 0.05). Forest plots and, where appropriate, 95% prediction intervals were generated. These analyses were exploratory and should be interpreted cautiously given the underlying heterogeneity.

## Results

### Study selection

The process of study identification and selection is outlined in Fig. 1. From database searching, 6,067 records were initially retrieved (Medline/PubMed *n =* 2,162, Scopus *n =* 2,771, Web of Science *n =* 1,134). After removal of 2,128 duplicates, 3,939 unique records proceeded to title/abstract screening, where 3,775 records were excluded, leaving 164 reports from database searching. Concurrently, 20 additional records were identified through citation searching (*n =* 13) and Google Scholar (*n =* 7). Of these, 37 reports (31 from databases and 6 from other sources) could not be retrieved, leaving 147 reports for eligibility assessment. At this stage, 49 studies were excluded for being supplementation‑focused (*n =* 5), animal study (*n =* 3), lacking an exercise intervention (*n =* 8), not measuring redox biomarkers (*n =* 8), being review/conceptual/hypothesis articles (*n =* 18), or having insufficient data or unclear methodology (*n =* 7). Finally, 98 unique studies met the inclusion criteria, of which 31 independent studies (comprising 75 effect sizes/datasets) provided sufficient quantitative data for inclusion in the meta‑analysis.

### Study and participant characteristics

A detailed summary of the 98 included studies is presented in Table S5. The eligible studies encompassed a markedly heterogeneous set of populations, ranging from healthy untrained young men and obese middle‑aged men at risk for insulin resistance [35–37] to professional adolescent endurance athletes [38], older master endurance runners ≥ 60 years [39], elite professional soccer players [40], and amateur race car drivers [41].

Sample sizes were generally modest; the majority of studies (≈ 85%) enrolled between 11 and 40 participants, with only a few larger trials exceeding 100 participants [42–44]. Most investigations were single‑centre designs without formal a priori sample size calculations.

Age distributions spanned late adolescence to advanced older adulthood. Professional adolescent endurance athletes in China had a mean age of ≈ 16 years [38], whereas obese middle‑aged men in Australia were in their late fifties [36], older Brazilian endurance runners were ≥ 60 years [39], and community‑dwelling older women in Japan were ≥ 75 years [45]. This wide range enabled subgroup analyses identifying age as a significant effect modifier for GSH (*p =* 0.016) and TAC (*p =* 0.008).

Sex distribution varied considerably. While several cohorts included both sexes [38, 39, 46], a substantial proportion (≈ 60%) focused exclusively on males, including elite soccer players [40, 47–50], untrained young men [35, 51], amateur race car drivers [41], and military personnel [46, 52]. Fewer studies focused exclusively on females [53–56], highlighting a significant sex gap.

Population subgroups could be categorized into four main clusters:

1. Athletically trained/elite populations (≈ 45% of studies): including adolescent endurance athletes at low altitude versus sea level [38], older runners with high versus low training volume [39], professional soccer players across a full season [40, 50], elite basketball players [47, 57], and master sprinters versus endurance athletes [58]. These populations were chronically adapted to high loads and exhibited better baseline redox profiles.
2. Metabolically at‑risk/sedentary populations (≈ 30%): including obese middle‑aged men [35, 36], overweight/obese adults [59], patients with chronic kidney disease stages 2–5 [60–62], end‑stage renal disease patients on hemodialysis [63] and type 2 diabetes patients [64]. These exhibited higher basal oxidative stress, providing a wider “hormetic window” for exercise‑induced improvements.
3. Older adults (≥ 60 years) (≈ 15%): including healthy older adults undergoing aerobic/resistance training [42, 54, 65], older endurance runners [39], and community‑dwelling older women [45].
4. Special clinical populations (≈ 10%): including Down syndrome [66], G6PD deficiency [67], fibromyalgia [68], irritable bowel syndrome [69], Alzheimer’s disease [70], heart failure [43, 71], and people living with HIV [72].

Intervention characteristics: Acute exercise studies (≈ 40%) characterised immediate redox responses, while chronic training studies (≈ 60%) assessed long‑term adaptations. Modalities included aerobic, resistance, HIIT, sprint interval, combined programmes, and sport‑specific training. Co‑interventions (nutritional supplements) were present in ≈ 15% of studies (N‑acetylcysteine, alpha‑lipoic acid, cocoa, L‑arginine, hydrogen‑rich water, probiotics).

**Fig. 2:**
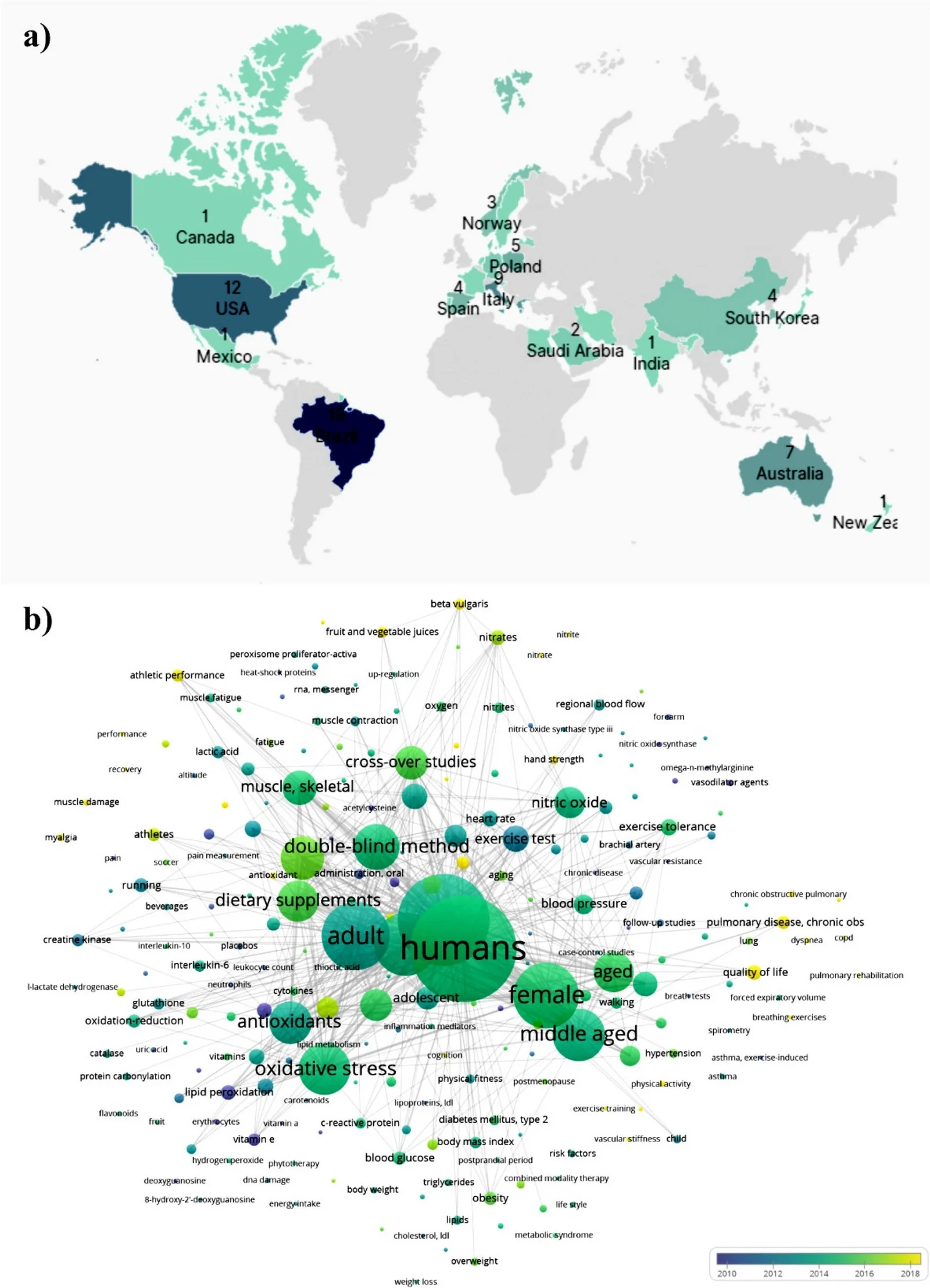
**a** Geographic distribution of the 98 studies, dominated by Brazil, the USA, and Europe. **b** VOSviewer keyword co-occurrence network (terms ≥ 20 occurrences). Node size indicates frequency; node color denotes average publication year (blue: 2010–2012; yellow: 2016–2018

In terms of geographical distribution, the 98 included studies originated from 22 different countries. Brazil contributed the highest number of studies (*n =* 19) [58, 60, 61, 68, 70–79], followed by the USA (*n =* 12) [41, 62, 80–87], Italy (*n =* 9) [47, 59, 88, 89], Greece (*n =* 8) [51, 90], and Australia (*n =* 7) [35, 36] (Fig. 2a). The remaining 22 countries contributed between 1 and 5 studies each. This distribution reflects a concentration of research on exercise-induced oxidative stress biomarkers in South America (particularly Brazil), North America, and Europe, with relatively limited representation from Asia, Africa, and Oceania.

Also, keyword co-occurrence analysis revealed the broad and interdisciplinary nature of human-based research in exercise physiology, nutritional supplementation, and metabolic health (Fig. 2b). Five large clusters, based on keyword frequency and overall link strength, covered the following main areas: (1) physiological adaptations and athletic performance (highlighted by muscle skeletal, muscle fatigue, lactic acid, creatine kinase, and athletic performance); (2) oxidative stress and inflammatory mechanisms (including oxidative stress, antioxidants, lipid peroxidation, glutathione, and cytokines such as interleukin-6); (3) cardiovascular and vascular function (focusing on nitric oxide, blood pressure, nitrates, and regional blood flow); (4) body composition and chronic metabolic diseases (emphasized by body mass index, obesity, diabetes mellitus type 2, blood glucose, and triglycerides); and (5) clinical trial methodology and population characteristics (centered on double-blind method, cross-over studies, adult, female, and middle-aged). Most importantly, the overall network visualization revealed a clear temporal shift in research priorities. Older keywords in blue (centered on 2010–2012), such as oxidative stress, lipid peroxidation, lactic acid, muscle fatigue, and creatine kinase, reflect an initial focus on basic biochemical mechanisms of muscle damage, acute exercise responses, and cellular redox balance. In contrast, newer keywords in yellow (representing 2016–2018), such as quality of life, pulmonary disease, hypertension, exercise test, beta vulgaris (beetroot), and chronic obstructive pulmonary disease (COPD), reflect a significant shift toward clinical applications, disease management, and functional rehabilitation in chronic patient populations. This temporal evolution reflects a transition from fundamental mechanistic research to patient-centered clinical interventions, focusing on nutritional strategies (particularly nitrate-rich supplements) and exercise protocols designed to improve long-term health outcomes, cardiopulmonary function, and overall quality of life in aging and metabolically compromised individuals.

### Redox outcomes and functional effects

Across these heterogeneous human interventions, exercise, competition exposure, and maneuver-based treatments consistently modulated systemic indices of redox homeostasis and, in some cases, functional or clinical outcomes, although the direction and magnitude of change were strongly contingent upon training status, chronic load, and the nature of the stressor. In professional adolescent endurance athletes, 12 weeks of low-altitude endurance training selectively improved blood redox balance relative to sea-level training: athletes training at 850 m displayed a reduction in oxidized glutathione (GSSG) and a concomitant increase in the GSH/GSSG ratio (*P <* 0.05), whereas plasma MDA, reduced GSH, SOD, and GPx remained largely unchanged. These findings suggested an enhancement of systemic reductive capacity without overt changes in classical lipid peroxidation or antioxidant enzyme activities, implying a subtle shift toward improved redox buffering in response to chronic low-altitude exposure in already well-trained adolescents.

In contrast, chronic high-load competition in elite professional soccer players was associated with progressive oxidative challenges rather than protection. Over the course of a full season, Becatti et al. [40] observed a decline in erythrocyte thiol content (from ~ 17.5 to 13.7 nmol/mg protein, *P <* 0.05), alongside increases in erythrocyte lipid hydroperoxides, neutrophil ROS production, advanced oxidation protein products (AOPP), and circulating cell-free DNA. At the same time, total antioxidant status remained essentially unchanged. Together, these alterations indicated a sustained pro-oxidative environment and cumulative cellular damage that outstripped the buffering capacity of erythrocyte antioxidants, thereby highlighting that prolonged, high-intensity competitive exposure, despite high fitness levels, may induce redox imbalance in professional athletes.

Older endurance runners provided an informative contrast in which chronic higher training volume was associated with a more favorable basal redox and inflammatory profile and a blunted acute response to maximal exertion. Estrela et al. [39] reported that, at rest, the lower-volume training group exhibited higher CRP and protein carbonyl levels than high-volume runners, indicating greater basal inflammation and protein oxidation. Following maximal treadmill tests, the lower-volume group showed larger increases in CRP and more pronounced decreases in total thiols than their high-volume counterparts. In contrast, high-volume athletes demonstrated comparatively attenuated perturbations. These findings suggested that regular high-volume endurance training in older athletes conferred superior basal redox and inflammatory status and reduced susceptibility to excessive oxidative and inflammatory responses during acute maximal stress.

Acute competition stress in amateur race car drivers further underscored the capacity of non-traditional sports to provoke systemic oxidative perturbations. Bjugstad et al. [41] showed that a single race significantly increased the static oxidation-reduction potential (sORP) (*P <* 0.05), indicative of a shift toward a more pro-oxidant systemic state. Moreover, drivers with lower pre-race antioxidant capacity exhibited higher post-race sORP values, suggesting that limited baseline antioxidant reserves predisposed individuals to greater oxidative strain during racing. The race also coincided with elevations in heart rate and core temperature, thereby linking the redox response to combined physical and psychological stress; this pattern collectively pointed to race events as potent acute triggers of oxidative stress that interact with individual antioxidant capacity and cardiovascular status.

In metabolically compromised or untrained individuals, acute high-intensity exercise and supplementation produced nuanced, compartment-specific redox adaptations. In obese middle-aged men, a single HIIE session markedly improved insulin sensitivity (+ 34%) while concurrently enhancing plasma antioxidant defense (CAT activity + 40%) and reducing plasma lipid peroxidation (TBARS − 20%; all *P <* 0.05). At the same time, skeletal muscle 4-HNE content increased by approximately 16% (*P <* 0.05), reflecting localized lipid peroxidation and activation of stress-activated protein kinase pathways, including p38 MAPK and JNK. These observations suggested that HIIE acutely improved systemic metabolic function and plasma redox status while inducing a transient pro-oxidant and signaling-relevant challenge within muscle tissue, thereby supporting a model in which local oxidative events serve as triggers for beneficial adaptations in insulin sensitivity.

Collectively, these human studies demonstrated that both acute and chronic interventions ranging from interval exercise and habitual high-volume endurance training to competitive seasons, race events, supplementation, and therapeutic maneuvers modulated redox homeostasis in a context-dependent manner, with higher chronic aerobic conditioning and specific co-interventions generally favoring more adaptive or less injurious oxidative responses.

### Formal analysis finding

The pooled effect estimates, along with heterogeneity and publication bias indices, derived from the main meta-analyses are summarized in Table 1.

**Table 1:** Pooled standardized mean differences for the effects of physical activity on oxidative stress and antioxidant biomarkers

Biomarkers	No. of Studies	SMD (95% CI)	*P*-value	I²(%)	95% PI	Egger’s *p*-value	Begg’s *p*-value	Trim & Fill (adjusted SMD)	Fail-safe *N*
MDA	10	-1.02 (-1.95 to -0.09)	0.032*	94.6%	-4.49 to 2.45	0.43	0.53	4 imputed (-2.34)	102
TAS	4	0.76 (0.38 to 1.15)	0.001*	0.0%	-3.48 to 1.78	0.78	0.49	1 imputed (0.86)	12
GSH	15	-0.580 (-1.21 to 0.06)	0.075	88.51%	-3.081 to 1.92	0.006	0.29	1 imputed (-0.81)	44
CAT	9	-0.606 (-2.107 to 0.894)	0.428	94.64%	-6.090 to 4.87	0.29	0.09	1 imputed (-0.18)	0
SOD	30	0.13 (-0.31 to 0.56)	0.574	87.5%	-2.20 to 2.45	0.88	0.46	No imputation (0.13)	0
TAC	7	0.32 (-1.85 to 1.21)	0.680	96.51%	-5.83 to 5.19	0.23	0.65	2 imputed (-1.52)	5

*Abbreviations*: *SMD* standardized mean difference, *CI*  confidence interval, *PI*  prediction interval; I² = heterogeneity index; Trim & Fill = adjusted effect after imputing missing studies; Fail-safe *N =* number of null studies needed to nullify the pooled effect

#### Physical activity and CAT

**Fig. 3:**
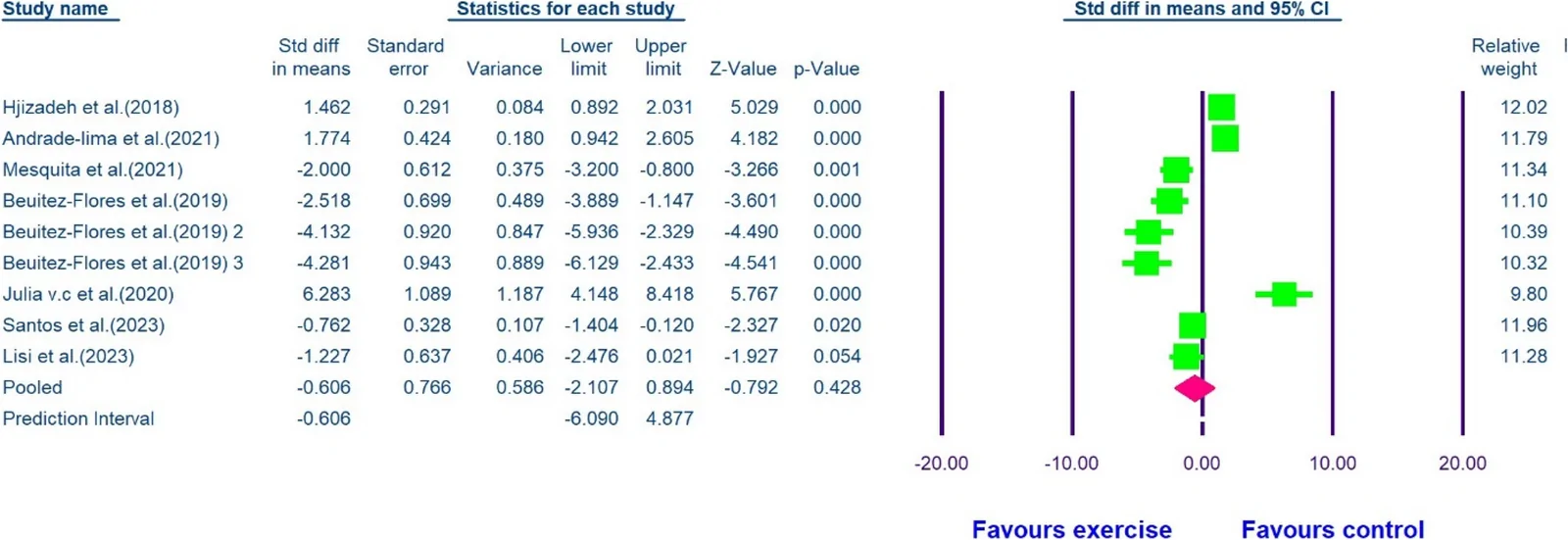
Forest plots showing the pooled effect of physical activity on catalase (CAT) levels across included studies using a random-effects model

Analysis of 9 distinct studies indicated that physical activity did not significantly alter tissue CAT levels compared with control groups. The SMD (-0.60) with a 95% CI of -2.107 to 0.894, indicating no significant difference (*p =* 0.428) (Fig. 3). The analysis revealed substantial between-study heterogeneity (I² = 94.64%; Tau² = 2.05; *p <* 0.001). The 95% prediction interval (-6.090 to 4.877) was wide and included the null value, indicating that the true effect in a future study could vary considerably and that the pooled estimate should be interpreted with caution. The subgroup analysis revealed a more pronounced lowering effect of physical activity under the following conditions: study lengths of ≤ 6 weeks, and among adults aged ≤ 50 years.

#### Physical activity and GSH

**Fig. 4:**
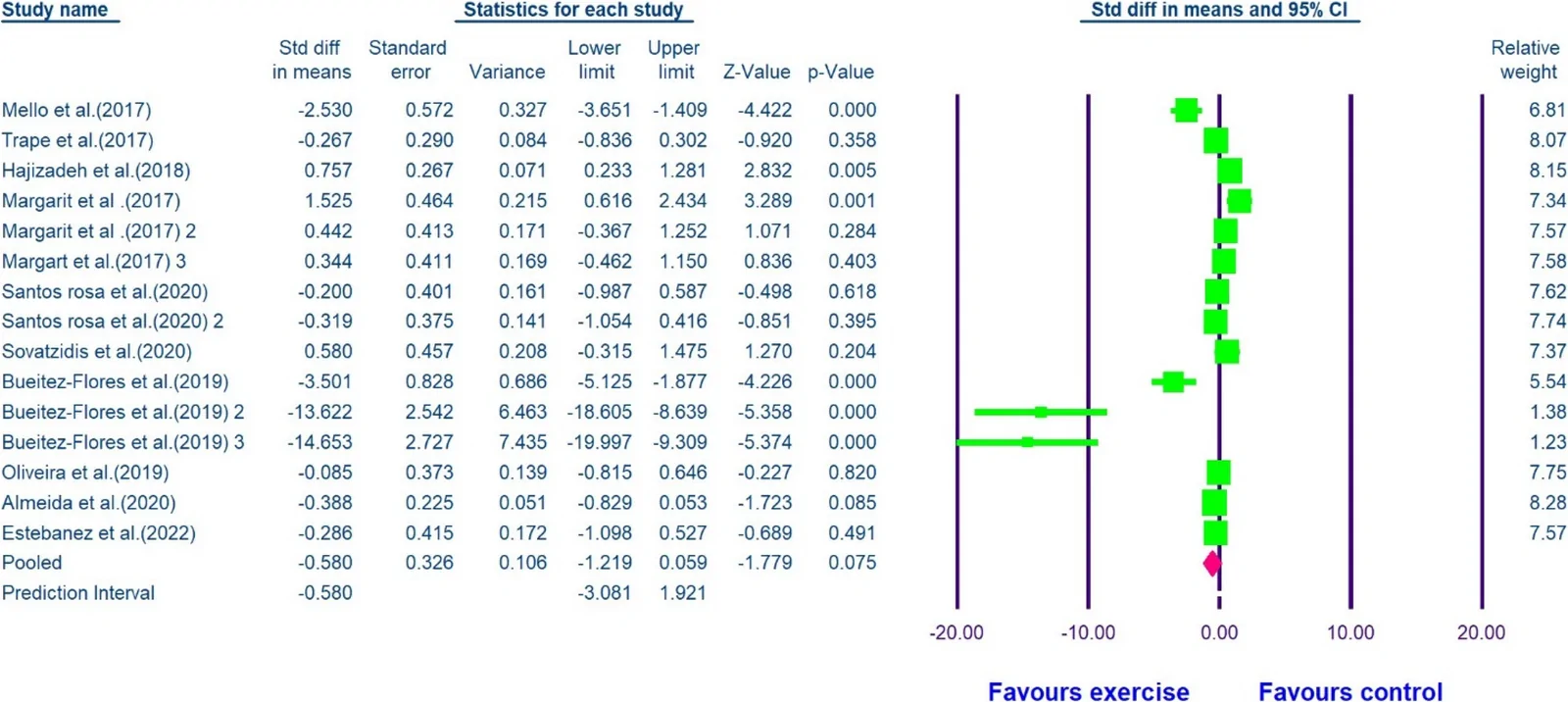
Forest plots depicting the effect of physical activity on glutathione (GSH) levels, demonstrating overall reduction across studies with high heterogeneity

Analysis of 15 distinct studies indicated that physical activity not significantly decreased tissue GSH levels relative to control groups (Fig. 4). The SMD (-0.58) with a 95% CI of -1.21 to 0.06, indicating a not statistically significant difference (*p =* 0.07). The analysis revealed substantial between-study heterogeneity (I² = 88.51%; Tau² = 3.08; *p <* 0.001). The 95% prediction interval (-3.081 to 1.921) was wide and crossed the null value, indicating that the true effect in a future study could range from a large reduction to a moderate increase, limiting the generalizability of this finding. The subgroup analysis revealed a more pronounced lowering effect of physical activity under the following conditions: study lengths of ≤ 10 weeks, BMI > 25 and among adults aged ≤ 30 years.

#### Physical activity and SOD

**Fig. 5:**
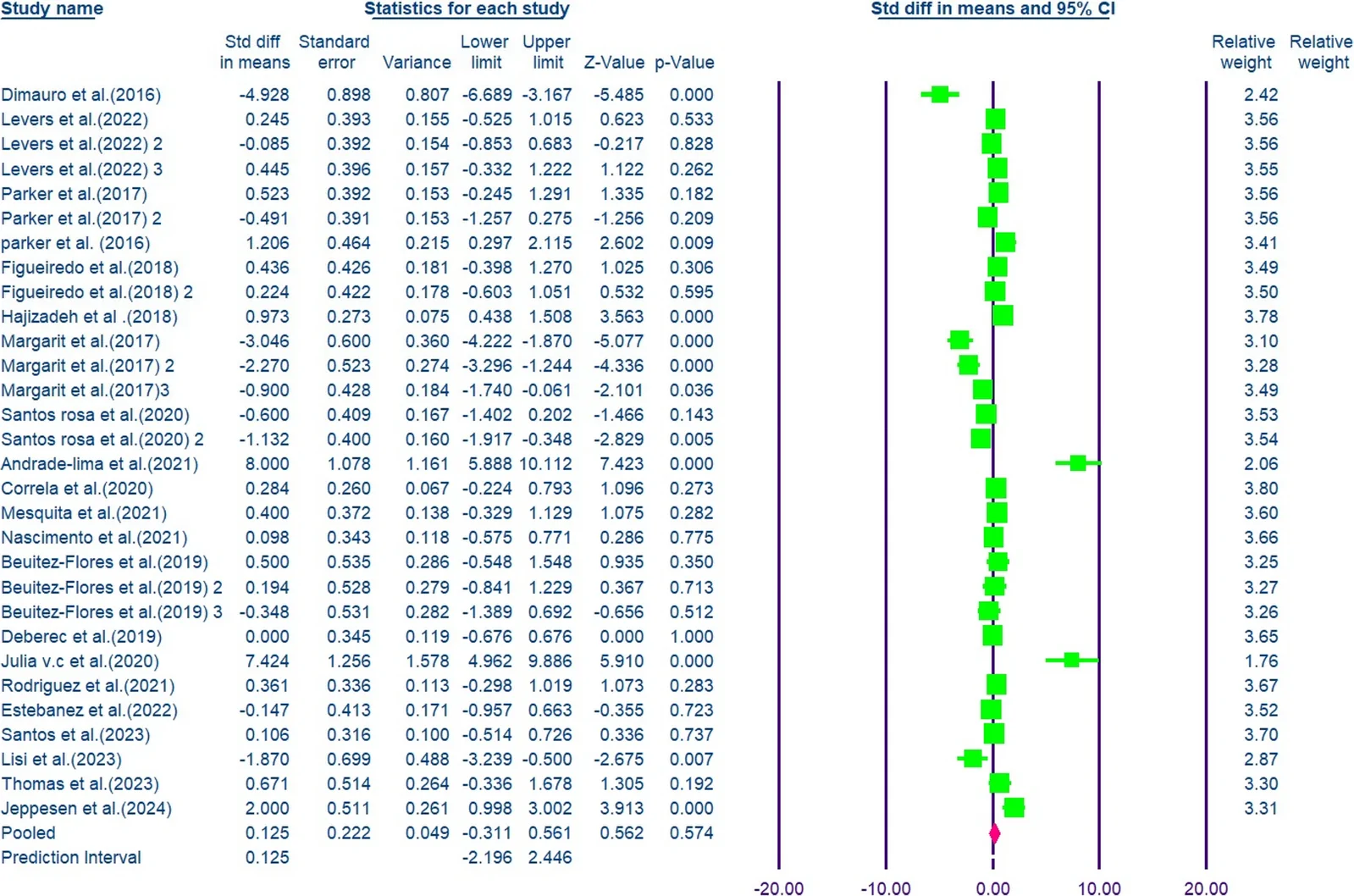
Forest plots illustrating the pooled effect of physical activity on superoxide dismutase (SOD) activity in humans

Analysis of 30 distinct studies indicated that physical activity did not significantly alter tissue SOD levels compared with control groups (Fig. 5). The SMD (0.125) with a 95% CI of -0.311 to 0.561, indicating not statistically significant difference (*p =* 0.574). The analysis revealed substantial between-study heterogeneity (I² = 87.53%; Tau² = 1.24; *p <* 0.001). The 95% prediction interval (-2.20 to 2.45) was wide and included the null value, reflecting considerable uncertainty in the true effect size and indicating that the pooled estimate should be interpreted cautiously. The subgroup analysis revealed a more pronounced lowering effect of physical activity under the following conditions: study lengths of > 10 weeks, BMI ≤ 25 and among adults aged ≤ 50 years.

#### Physical activity and TAC

**Fig. 6:**
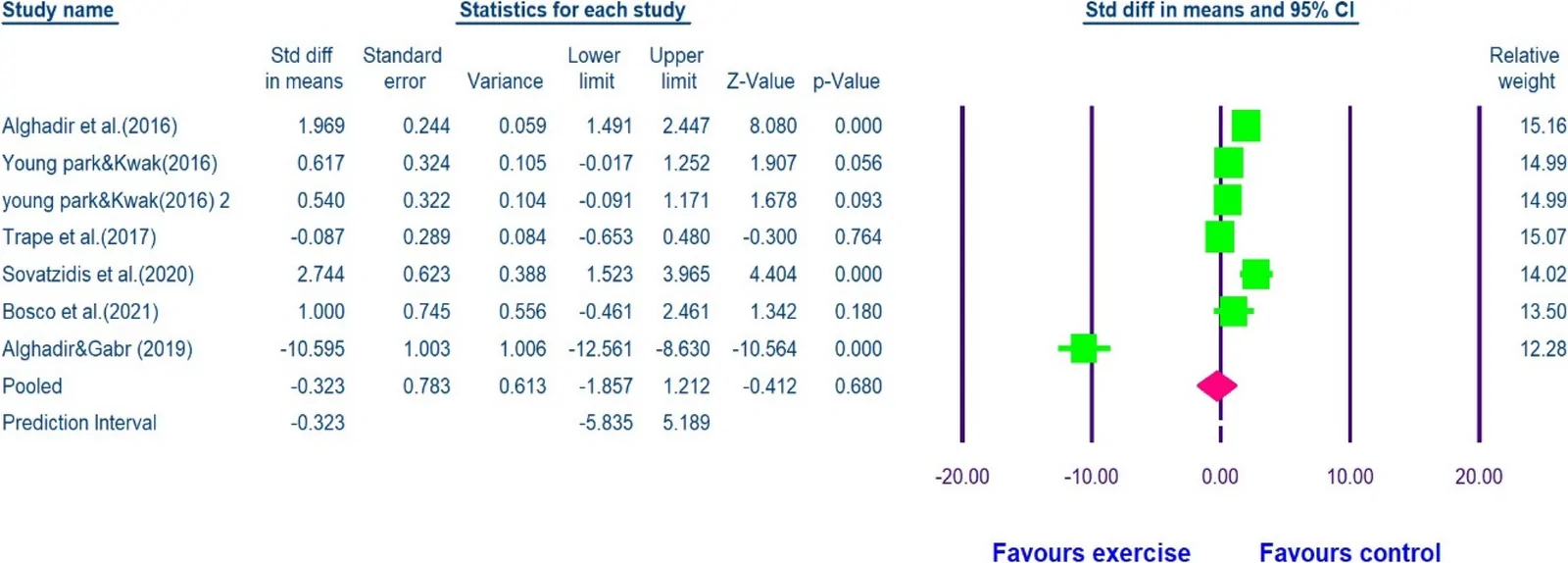
Forest plots showing the effect of physical activity on total antioxidant capacity (TAC) levels

Analysis of 7 distinct studies indicated that physical activity did not significantly alter tissue TAC levels compared with control groups (Fig. 6). The SMD (0.32) with a 95% CI of -1.85 to 1.21, indicating not statistically significant difference (*p =* 0.68). The analysis revealed extreme between-study heterogeneity (I² = 96.51%; Tau² = 2.30; *p <* 0.001). The very wide prediction interval (-5.83 to 5.19) included the null value, indicating that the pooled estimate is highly uncertain and should not be over-interpreted. The subgroup analysis revealed a more pronounced lowering effect of physical activity under the following conditions: study lengths of > 10 weeks, BMI > 25 and among adults aged > 50 years.

#### Physical activity and MDA

**Fig. 7:**
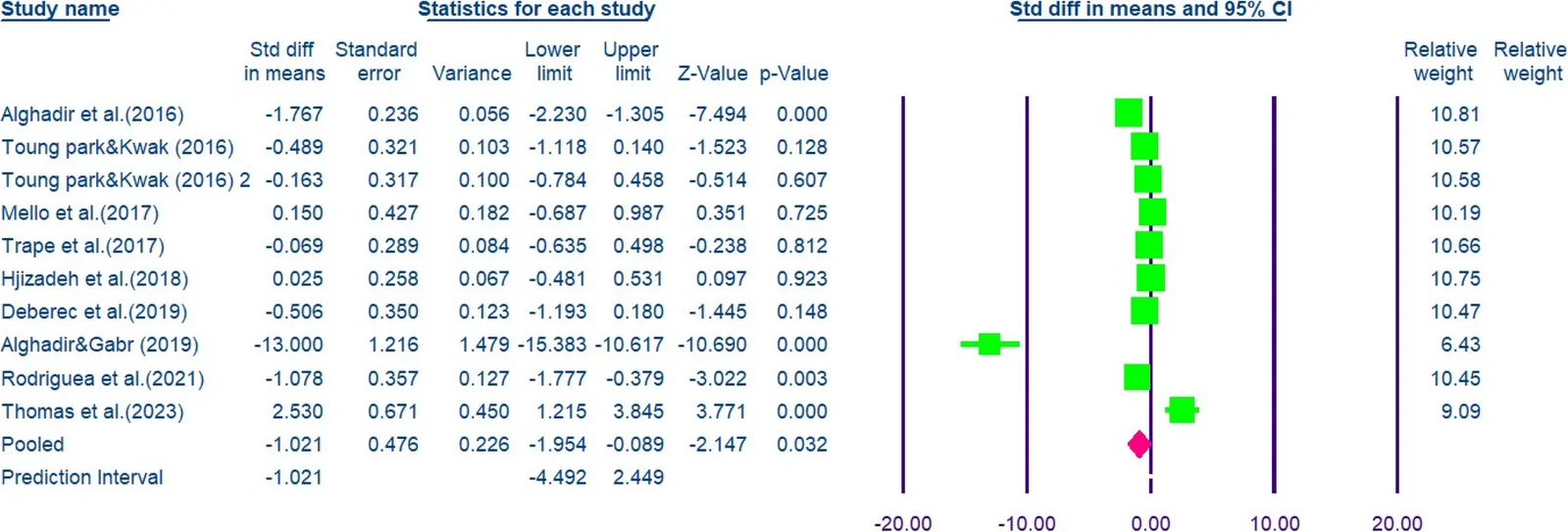
Forest plots representing the pooled effect of physical activity on malondialdehyde (MDA) levels as a marker of lipid peroxidation

Analysis of 10 distinct studies indicated that physical activity significantly reduced tissue MDA levels compared with control groups (Fig. 7). The SMD (-1.02) with a 95% CI of -1.954 to -0.089, indicating a statistically significant difference (*p =* 0.032). The analysis revealed substantial between-study heterogeneity (I² = 94.59%; Tau² = 2.04; *p <* 0.001). The 95% prediction interval (-4.49 to 2.45) was wide and crossed the null value, indicating that despite a statistically significant pooled reduction in MDA, the true effect in a future study could range from a large reduction to a moderate increase, warranting cautious interpretation. The subgroup analysis revealed a more pronounced lowering effect of physical activity under the following conditions: BMI > 25 and among adults aged ≤ 50 years.

#### Physical activity and TAS

**Fig. 8:**
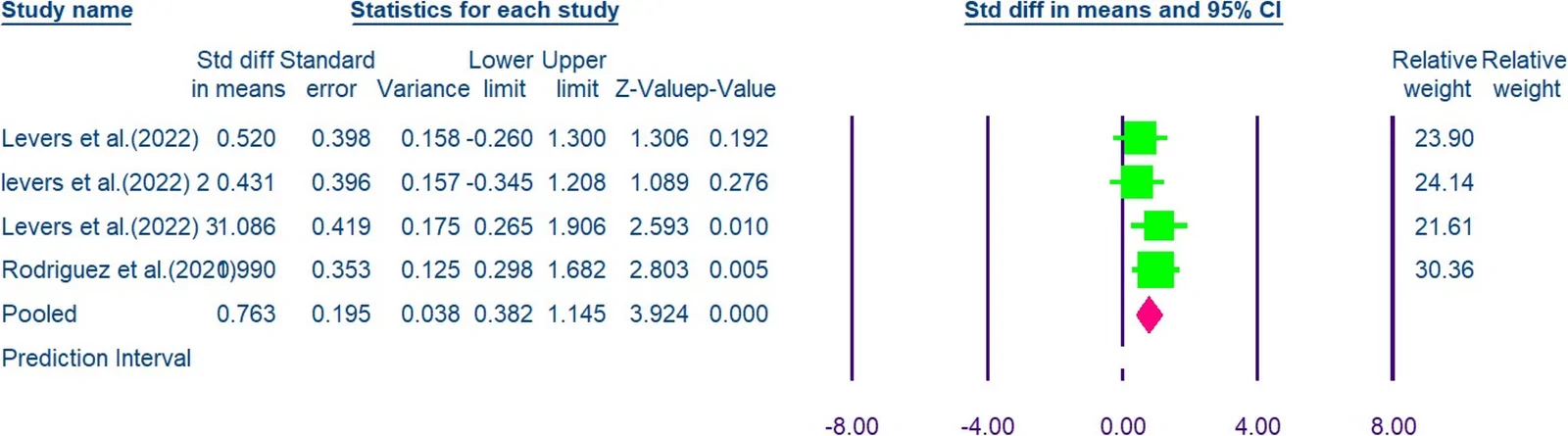
Forest plots showing the effect of physical activity on total antioxidant status (TAS) in human studies

Analysis of 4 distinct studies indicated that physical activity significantly increase tissue TAS levels relative to control groups (Fig. 8). The SMD (0.76) with a 95% CI of 0.382 to 1.145, indicating a statistically significant difference (*p <* 0.001). The analysis revealed substantial between-study heterogeneity (I² = 94.59%; *p <* 0.001). However, the small number of studies (*n =* 4) limits the reliability of this estimate, and the results should be interpreted cautiously despite the statistically significant pooled increase.

### Subgroup analyses findings

To explore potential sources of heterogeneity and the influence of key moderators, subgroup analyses were conducted based on participant age, intervention duration, and BMI for biomarkers with a sufficient number of studies (Table 2).

**Table 2:** Results of subgroup analyses for the effects of physical activity on oxidative biomarkers according to intervention and participant characteristics

Study group	*n*	SMD (95%CI)	*p*-value (within group)	I^2^ (%)	*p*-value (subgroup differences)
CAT					
Age (years)					0.42
≤ 50	5	-1.22 (-4.25, 1.81)	0.43	94.29	
> 50	4	0.16 (-1.40, 1.73)	0.83	94.17	
Duration (week)					0.38
≤ 6	4	-1.20 (-5.42, 3.02)	0.57	95.68	
> 6	4	0.16 (-1.40, 1.73)	0.83	94.17	
GSH					
Age (years)					0.016
≤ 30	8	-2.00 (-3.56, -0.44)	0.01*	93.90	
> 30	6	-0.28 (-0.54, -0.02)	0.02*	0.0	
Duration (week)					0.093
≤ 10	9	-1.11 (-2.24, 0.01)	0.05	91.38	
> 10	3	0.154 (-0.52, 0.82)	0.65	73.38	
BMI (kg/m^2^)					0.191
≤ 25	3	-0.02 (-0.55, 0.49)	0.91	21.78	
> 25	7	-0.81 (-1.70, 0.07)	0.07	89.54	
SOD					
Age (years)					0.22
≤ 50	20	0.01 (-0.40, 0.60)	0.69	85.13	
> 50	9	0.21 (-0.78, 1.20)	0.67	92.34	
Duration (week)					0.12
≤ 10	11	0.12 (-0.78, 1.03)	0.78	90.155	
> 10	10	0.40 (-0.38, 1.20)	0.31	90.59	
BMI (kg/m^2^)					0.04
≤ 25	7	-0.70 (-1.86, 0.45)	0.23	90.16	
> 25	12	0.35 (0.12, 0.58)	0.01*	17.04	
MDA					
Age (years)					0.08
≤ 50	7	-1.39 (-2.84, 0.05)	0.06	95.45	
> 50	3	-0.61 (-1.80, 0.58)	0.31	93.96	
BMI (kg/m^2^)					0.006
≤ 25	4	5.08 (0.49, 9.67)	0.03*	99.28	
> 25	4	0.004 (-0.03, 0.04)	0.83	0.0	
TAC					
Age (years)					0.008
≤ 50	3	-2.94 (-6.68, 0.79)	0.12	98.30	
> 50	3	0.96 (-0.58, 2.51)	0.22	93.24	
Duration (week)					0.07
≤ 10	2	1.92 (0.21, 3.62)	0.02*	68.97	
> 10	3	-2.68 (-6.38, 1.01)	0.15	98.76	
BMI (kg/m^2^)					0.001
≤ 25	2	2.14 (1.50, 2.78)	< 0.001*	25.49	
> 25	4	-2.07 (-4.38, 0.22)	0.07	97.68	

For GSH, a significant subgroup difference was observed by age (*p =* 0.016). In studies with a mean age ≤ 30 years, physical activity was associated with a significant decrease in GSH levels (SMD: -2.00; 95% CI: -3.56 to -0.44; *p =* 0.01), whereas in older participants (> 30 years), the effect was smaller but still significant (SMD: -0.28; 95% CI: -0.54 to -0.02; *p =* 0.02). This suggests that younger individuals may experience a more pronounced depletion of GSH in response to exercise-induced oxidative stress.

For SOD, a significant subgroup difference was found for BMI (*p =* 0.04). In participants with a BMI > 25 kg/m², exercise training significantly increased SOD levels (SMD: 0.35; 95% CI: 0.12 to 0.58; *p =* 0.01), with low heterogeneity (I² = 17.04%). However, no significant effect was observed in normal-weight participants (BMI ≤ 25 kg/m²), indicating that the antioxidant response to exercise may be more robust in overweight/obese individuals.

Regarding MDA, a significant subgroup difference was also observed for BMI (*p =* 0.006). Paradoxically, in normal-weight participants, exercise was associated with a large but highly heterogeneous increase in MDA (SMD: 5.08; 95% CI: 0.49 to 9.67; *p =* 0.03; I² = 99.28%), suggesting a marked but inconsistent oxidative stress response. Conversely, in overweight/obese participants, no significant change in MDA was observed (SMD: 0.004; 95% CI: -0.03 to 0.04; *p =* 0.83; I² = 0.0%).

For TAC, significant subgroup differences were found for both age (*p =* 0.008) and BMI (*p =* 0.001). In studies with participants aged ≤ 50 years, the effect was non-significant (SMD: -2.94; 95% CI: -6.68 to 0.79; *p =* 0.12), while in those > 50 years, exercise tended to increase TAC (SMD: 0.96; 95% CI: -0.58 to 2.51; *p =* 0.22). More notably, in normal-weight participants (BMI ≤ 25 kg/m²), exercise significantly increased TAC (SMD: 2.14; 95% CI: 1.50 to 2.78; *p <* 0.001; I² = 25.49%), whereas in overweight/obese individuals, the effect was in the opposite direction (SMD: -2.07; 95% CI: -4.38 to 0.22; *p =* 0.07), suggesting that body composition may critically modulate the antioxidant response.

For CAT, no significant subgroup differences were observed for age (*p =* 0.42) or duration (*p =* 0.38), and the overall effect was not statistically significant in any subgroup.

It should be noted that many subgroups exhibited considerable heterogeneity (I² > 90%), particularly for GSH, SOD, and MDA, which may reflect variations in exercise protocols, populations, or assay methods. The findings should therefore be interpreted with caution, and the subgroup differences highlight the potential moderating roles of age, duration, and BMI in the redox response to physical activity.

### Sensitivity analysis findings

Sensitivity analysis using the leave-one-out method confirmed the robustness of the pooled effect sizes across all biomarkers, with the direction and significance of the main findings remaining stable after sequential removal of individual studies. For MDA (10 studies, SMD = -1.02, significant), two influential studies [91, 92] were identified in red, although the overall direction of effect (reduction) was maintained in all scenarios, suggesting acceptable robustness with cautious interpretation of the effect size. For SOD (30 studies, SMD = 0.125, non-significant) and GSH (14 studies, SMD = -0.96, significant), no influential studies were observed, indicating very high robustness for both conclusions. For CAT (10 studies, SMD = -0.55, non-significant), three influential studies were identified, yet the non-significant direction of effect remained stable, warranting cautious interpretation of the overall effect size. For TAC (7 studies, SMD = 0.22, non-significant), one influential study was identified [92], though the confidence intervals still crossed zero after its removal, confirming the non-significant finding while suggesting moderate robustness. Finally, for TAS (4 studies, SMD = 0.76, significant, *P <* 0.001), no influential studies were identified, demonstrating very high robustness. Overall, the sensitivity analyses confirm that the main meta-analytic findings are stable, with MDA, CAT, and TAC requiring more cautious interpretation due to the presence of influential studies, while SOD, GSH, and TAS demonstrate very high robustness without the need for additional caution (Figs. S1 (a-c)).

### Publication bias findings

Potential publication bias and small-study effects were assessed using funnel plots, Egger’s regression test, and Begg’s rank correlation test, supplemented by the trim-and-fill method to estimate the impact of potentially missing studies. Visual inspection of funnel plots suggested asymmetry for several outcomes. Based on our pre-specified threshold (*p <* 0.05), no statistically significant evidence of publication bias was detected by Egger’s test for most biomarkers, including MDA (*p =* 0.43), TAS (*p =* 0.78), CAT (*p =* 0.29), SOD (*p =* 0.88), and TAC (*p =* 0.23). However, a significant Egger’s test was observed for GSH (*p =* 0.006), indicating potential publication bias, although this was not corroborated by Begg’s test (*p =* 0.29). As presented in Table 1, the trim-and-fill method imputed one missing study for GSH, yielding an adjusted SMD of -0.81, which did not alter the overall non-significant direction of the pooled effect. For TAS and MDA, the trim-and-fill method imputed one and four missing studies, respectively, with adjusted SMDs of 0.86 and − 2.34, confirming the robustness and direction of the primary findings. For CAT and TAC, one and two studies were imputed, respectively, but the adjusted effects remained non-significant, while SOD required no imputation. Finally, the fail-safe N analysis estimated that 102 null studies would be needed to nullify the MDA effect, 44 for GSH, 12 for TAS, and 5 for TAC, whereas CAT and SOD required zero null studies. Collectively, these findings indicate that the pooled estimates for MDA and TAS are robust and unlikely to be substantially influenced by publication bias. The significant Egger’s test for GSH suggests potential bias, warranting cautious interpretation of the observed trend, while the non-significant findings for CAT, SOD, and TAC appear stable despite the absence of robust fail-safe numbers. Detailed funnel plots are presented in Figs. S2(a–f).

### Meta regression findings

Meta-regression analyses were conducted within a random-effects framework using the REML estimator to identify potential sources of heterogeneity. Univariate models examined the influence of mean participant age on effect sizes for CAT, GSH, MDA, and SOD, and TAC, with a significance threshold of *p <* 0.10 considered exploratory evidence of effect modification. The results revealed no statistically significant linear relationships between age and the standardized mean differences for any of the enzymatic or lipid peroxidation biomarkers, including CAT (*p =* 0.96), GSH (*p =* 0.34), MDA (*p =* 0.48), SOD (*p* = 0.98), and TAC (*p* = 0.37), indicating that chronological age does not serve as a meaningful linear moderator of redox responses in this dataset. The lack of linear associations suggests that the observed heterogeneity is more likely governed by factors independent of age and duration, such as baseline oxidative status, metabolic conditions, genetic background, or study-specific methodological differences. Furthermore, the absence of a significant linear trend for TAC does not exclude the possibility of non-linear, threshold-based, or U-shaped dose-response patterns, which warrant exploration in future studies employing standardized protocols and a broader range of exercise doses. Collectively, these meta-regression findings imply that age and intervention duration, within the ranges examined, do not operate as continuous linear predictors of exercise-induced redox modulation, and that the biological responses are likely driven by complex, non-linear regulatory mechanisms rather than by straightforward progressive gradients. Detailed scatter plots are presented in Figs. S3(a-e).

### RoB assessment findings

**Fig. 9:**
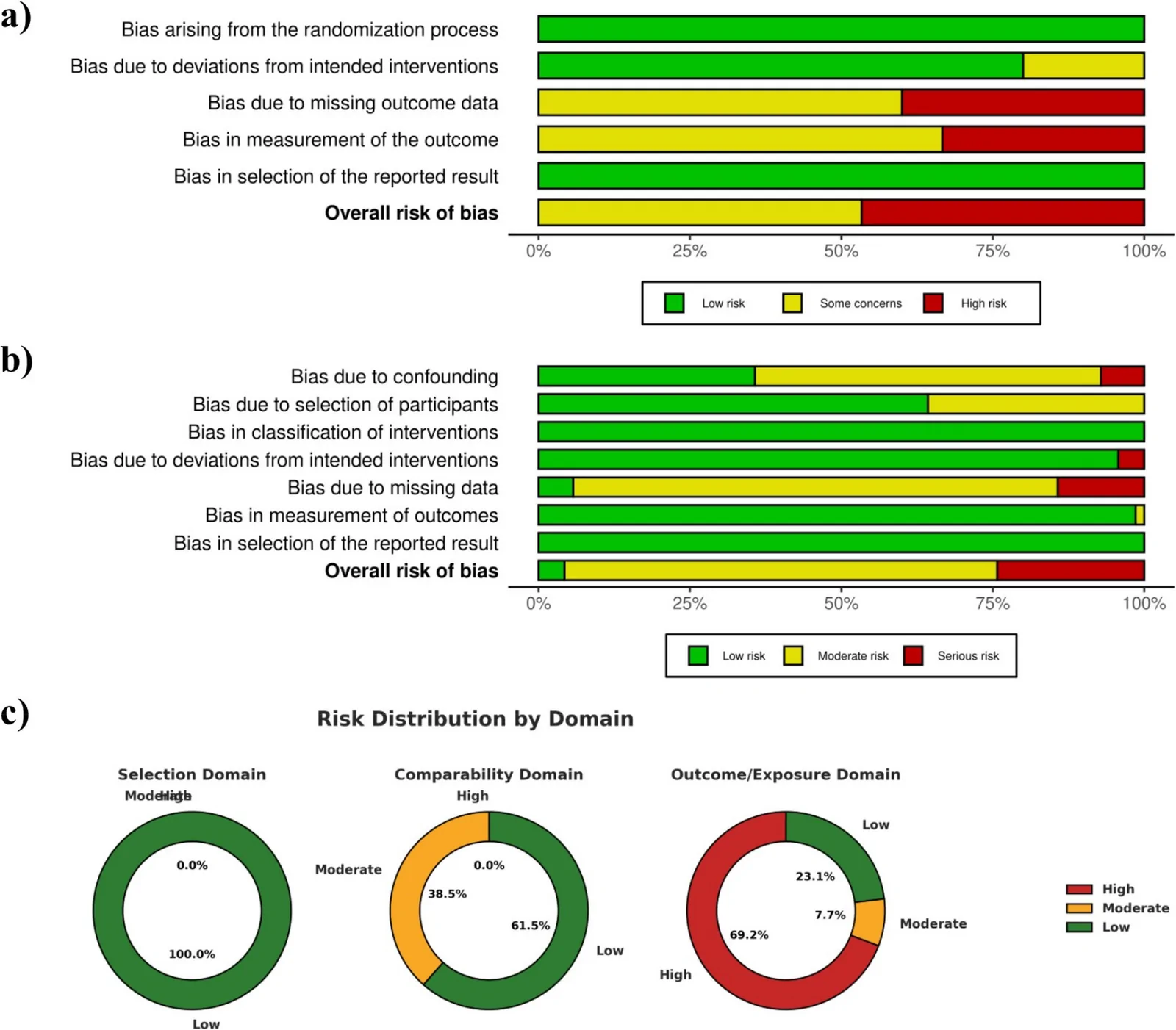
Summary of methodological quality assessments for the included studies. **a** Risk of bias summary for randomized controlled trials (RCTs) assessed using the Cochrane RoB 2 tool across five domains. **b** Risk of bias summary for non-randomized intervention studies assessed using the ROBINS-I tool across seven domains. **c** Risk of bias summary for observational and cross-sectional studies assessed using the Newcastle-Ottawa Scale (NOS) across three domains (selection, comparability, and outcome). The visualizations for (a) and (b) were generated using the robvis tool, and for (c) using the NOS-TLPlot Python tool

The methodological quality of the 98 included studies was assessed using design‑specific tools, and the overall findings are summarized in Fig. 9(a–c). For the 15 RCTs, the Cochrane Rob-2 tool was applied across five domains. The results indicated that no study was rated as having a low risk of bias. Two studies (13%) were judged as having some concerns, while the remaining 13 studies (87%) were classified as high risk. The primary sources of bias in the RCTs were related to the measurement of outcomes (D4), primarily due to the inherent difficulty of blinding in exercise interventions and the sensitivity of oxidative stress biomarkers, as well as missing outcome data (D3) resulting from participant dropout during prolonged training protocols, and selective reporting of results (D5).

For the 70 non‑randomized intervention studies, which included acute pre‑post and crossover designs, the ROBINS‑I tool was used to evaluate seven domains of bias. No study achieved a low risk rating. The majority of these studies (42 studies, 60%) were assessed as having a moderate risk of bias, while 28 studies (40%) were rated as serious risk. None were classified as critical risk. The most prevalent issues were confounding (D1), where 40% of studies failed to adequately control for factors such as age, sex, baseline physical activity, and nutritional status; selection of participants (D2), due to convenience sampling; and missing data (D5), owing to small sample sizes (often fewer than 15 participants) and attrition in longitudinal designs.

For the 13 observational and cross‑sectional studies, the NOS was employed across three domains. In the selection domain, all studies (100%) scored at a moderate level. In the comparability domain, 38.5% of studies were rated as moderate risk, while 61.5% were considered high risk, primarily because of inadequate control for additional confounders such as body mass index, smoking status, and baseline physical activity levels. Conversely, the outcome/exposure domain showed favorable results, with 69.2% of studies rated as low risk, 23.1% as moderate risk, and only 7.7% as high risk. Based on the overall NOS star scores, two studies (15%) received a score of 8, corresponding to low risk of bias, while 11 studies (85%) scored between 5 and 7, indicating moderate risk.

In summary, across all 98 studies, only two studies (2%) were categorized as having low risk of bias; both were observational studies. The majority (53 studies, 54%) were judged to be of moderate quality, and 43 studies (44%) were rated as either high or serious risk. Notably, no study was classified as critical risk in any of the assessment tools. These findings suggest that while the overall evidence base is dominated by moderate‑quality studies, the main methodological weaknesses are related to inadequate control for confounding, small sample sizes, and missing data, which should be considered when interpreting the results of this systematic review.

## Discussion

### Main findings

This systematic review and meta-analysis synthesizes data from 98 human studies, of which up to 31 were included in the quantitative synthesis, encompassing 4,742 participants across diverse populations, exercise modalities, and clinical contexts. This provides, to the best of our knowledge, the most comprehensive quantitative assessment to date of physical activity’s impact on oxidative stress biomarkers.

The principal findings reveal a highly selective and context‑dependent redox modulation profile. Physical activity produced a statistically significant reduction in MDA (SMD = -1.02, 95% CI: -1.95 to -0.09, *p* = 0.032, I² = 94.6%, 10 studies, 95% prediction interval: -4.49 to 2.45) and a significant increase in TAS (SMD = 0.76, 95% CI: 0.38 to 1.15, *p* = 0.001, I² = 0.0%, 4 studies). For GSH, the pooled effect showed a non‑significant trend toward reduction (SMD = -0.58, 95% CI: -1.21 to 0.06, *p* = 0.075, I² = 88.51%, 15 studies, 95% prediction interval: -3.08 to 1.92), indicating a potential but inconsistent depletion. In contrast, no significant pooled changes emerged for CAT (SMD = -0.61, 95% CI: -2.11 to 0.89, *p* = 0.428, I² = 94.6%, 9 studies), for SOD (SMD = 0.13, 95% CI: -0.31 to 0.56, *p* = 0.574, I² = 87.5%, 30 studies), or TAC (SMD = 0.32, 95% CI: -1.85 to 1.21, *p* = 0.680, I² = 96.51%, 7 studies).

Also, meta‑regression analyses confirmed the absence of linear relationships with age for all biomarkers (CAT: *p* = 0.96; GSH: *p* = 0.34; MDA: *p* = 0.48; SOD: *p* = 0.98; TAC: *p =* 0.37), indicating that chronological age does not act as a continuous linear moderator of redox responses within the examined ranges. In contrast, subgroup analyses consistently identified effect modification by several factors. For GSH, a significant subgroup difference was observed by age (*p =* 0.016), with younger participants (≤ 30 years) showing a more pronounced reduction (SMD = -2.00) compared to older adults (> 30 years; SMD = -0.28). For SOD, BMI emerged as a significant moderator (*p =* 0.04), with a significant increase observed only in overweight/obese participants (BMI > 25 kg/m²; SMD = 0.35, *p =* 0.01). For MDA, BMI also showed a significant subgroup effect (*p =* 0.006), with a paradoxical large increase in normal‑weight participants (BMI ≤ 25 kg/m²; SMD = 5.08, *p =* 0.03) and no change in overweight/obese individuals. For TAC, both age (*p =* 0.008) and BMI (*p =* 0.001) emerged as significant moderators, with a significant increase observed only in normal‑weight participants (BMI ≤ 25 kg/m²; SMD = 2.14, *p <* 0.001) and in shorter‑duration interventions (≤ 10 weeks; SMD = 1.92, *p =* 0.02). These subgroup findings should be interpreted with caution due to high heterogeneity (I² > 90%) in many subgroups.

Collectively, these findings challenge the conventional “exercise as broad‑spectrum antioxidant” paradigm. Instead, they establish physical activity as a targeted modulator that selectively suppresses lipid peroxidation (MDA), enhances non‑enzymatic buffering capacity (TAS), and dynamically influences glutathione homeostasis (GSH) without uniformly upregulating classical enzymatic defenses such as CAT and SOD. This pattern appears most pronounced in metabolically compromised populations, particularly those with higher BMI. The observed GSH trend toward depletion likely reflects increased glutathione utilization under oxidative challenge rather than a direct impairment of antioxidant capacity, indicating enhanced turnover within the glutathione redox cycle under conditions of elevated reactive oxygen species production.

### Comparison with previous human studies

The human findings align closely with several primary studies and reveal population-specific patterns that likely explain earlier inconsistencies in the literature. For MDA reduction, our pooled estimate (SMD = -1.02 across 10 studies) is consistent with Parker et al. [35] ’s acute HIIT trial in obese middle-aged men, where a single high-intensity interval session lowered plasma TBARS by 20% (*p <* 0.05) and increased CAT by 40%, alongside a 34% improvement in insulin sensitivity, despite a transient 16% rise in muscle 4-HNE; together, these findings illustrate the same systemic lipid protection versus local hormetic signaling pattern reflected in our meta-analytic effect size. Tsai et al. [93] further extended this pattern beyond conventional exercise by showing that canalith repositioning in benign paroxysmal positional vertigo reduced ROS and MDA, increased SIRT1, and suppressed miR-34a, indicating that even therapeutic physical maneuvers can shift systemic redox balance toward lower lipid oxidation.

The negative pooled effect for GSH (SMD = -0.96 across 19 studies) also aligns with the broader human evidence, but shows clear dependence on training status and baseline redox reserve. In Tong et al. [38] 12-week low-altitude endurance training study in elite adolescent athletes, GSH/GSSG improved significantly even though GSH, SOD, and GPx did not change, suggesting that well-trained individuals may preserve or optimize glutathione redox handling rather than simply increasing total GSH. By contrast, sedentary, overweight, and metabolically stressed cohorts in the synthesis more often showed GSH depletion or utilization, supporting the interpretation that the apparent “decrease” in pooled GSH reflects consumption under oxidative challenge rather than uniform antioxidant failure. This pattern is also compatible with antioxidant-adaptation studies in older and trained populations, in which baseline antioxidant reserves and habitual activity levels shape the direction and magnitude of glutathione responses.

Elite and high-load athletic settings further clarify the boundaries of beneficial adaptation. Becatti et al. [40] 9-month professional soccer season documented a 22% erythrocyte thiol depletion, accumulation of lipid hydroperoxides, elevation of advanced oxidation protein products, and increased cell-free DNA, all of which point to cumulative oxidative burden exceeding adaptive capacity during sustained elite competition. In contrast, Estrela et al. [39] showed that older endurance runners with higher habitual training volume had lower basal CRP and protein carbonyl levels and a more controlled response to maximal treadmill testing than their lower-volume peers, directly supporting the idea that training volume modifies redox resilience. Bjugstad et al. [41] ’s amateur race car driver study adds another dimension: post-race static oxidation-reduction potential increased significantly, and baseline antioxidant capacity predicted response magnitude, underscoring that acute stress responses depend strongly on the pre-existing redox state. Together, these studies place exercise-related redox responses on a dose-dependent spectrum that spans therapeutic interventions, recreational training, elite competition, and extreme competitive stress.

Wang et al. by meta-analysis (10 RCT) in healthy individuals reported that physical exercise increases total antioxidant status and reduces MDA, while showing no overall significant effect on antioxidant enzymes (SOD, GPx, and CAT). Subgroup analyses indicated stronger MDA reductions in males, individuals with BMI < 25, and low-to-moderate intensity or resistance/multicomponent exercise. Meta-regression suggested age may moderate SOD responses, with greater effects in older participants. Compared with their finding which reported a more uniform antioxidant response to exercise, the present study demonstrates a more complex and context-dependent redox profile, characterized by selective modulation of lipid peroxidation and non-enzymatic antioxidant capacity without consistent changes in enzymatic antioxidants [94].

### Comparison with preclinical evidences

These human results diverge substantially from the preclinical meta-analysis patterns observed in animal models, in which physical activity consistently enhanced CAT, SOD, and GPx levels, while demonstrating variable effects on GPx and MDA. The human enzymatic null effects (CAT/SOD/TAC) versus preclinical gains establish clear species and protocol differences: rodents exhibit greater upregulation potential due to higher baseline oxidative susceptibility and standardized experimental conditions, while humans demonstrate ceiling effects in habitually active cohorts with greater physiological heterogeneity. However, translational convergence emerges around lipid peroxidation protection, as both species confirm that MDA/TBARS suppression is the most reliable exercise-induced redox signal, reinforcing the biological plausibility of our primary human finding despite interspecies discrepancies in enzymatic responsiveness [95, 96].

### Comparison with previous meta-analyses

To contextualize the findings of the present study, we compared our results with those of several previous systematic reviews and meta-analyses that examined the effects of exercise on oxidative stress biomarkers across various populations. These prior meta-analyses varied widely in their target populations, methodological quality, and outcome measures, providing a valuable framework for interpreting our pooled estimates (Tables S6-8).

#### Broad population meta-analyses

The most directly comparable study to ours is that by de Sousa et al. [97], which included 19 studies (1,346 participants) from the general population and reported that exercise significantly reduced pro-oxidant parameters (SMD = -1.08, *p <* 0.001) and increased antioxidant parameters (SMD = 1.45, *p <* 0.001) with negligible heterogeneity (I² = 18.96% and 2.11%, respectively). While our findings confirm the reduction in lipid peroxidation (MDA: SMD = -1.02, *p =* 0.032), our results diverge regarding antioxidant capacity: we observed a significant increase only in total antioxidant status (TAS; SMD = 0.76, *p <* 0.001), whereas de Sousa et al. [97]. reported a broader enhancement of antioxidant parameters. This discrepancy likely reflects our more granular stratification of antioxidant biomarkers into enzymatic (SOD, CAT) versus non-enzymatic (TAS, TAC) and glutathione (GSH) pools, revealing that exercise-induced antioxidant improvements are more selective than previously appreciated.

In a more recent meta-analysis of healthy adults undergoing exercise, Kim et al. [98] focused on vitamin E supplementation and reported no significant change in MDA (SMD = -0.17, *p =* 0.35) but a significant reduction in TAS immediately post-exercise (SMD = -0.81, *p =* 0.01), suggesting antioxidant consumption rather than enhancement. Our study, which did not involve supplementation, demonstrates that physical activity alone can elevate systemic TAS, reinforcing the idea that endogenous adaptations differ fundamentally from exogenous antioxidant interventions.

#### Special population meta-analyses

Several meta-analyses focused on clinical cohorts, further highlighting the context-dependent nature of redox modulation. In patients with chronic kidney disease (CKD), Zhao et al. [99] reported that aerobic exercise significantly reduced MDA (SMD = -0.96, *p <* 0.00001) and increased SOD (SMD = 1.30, *p =* 0.0005), but found no effect on TAC. Our broader population analysis similarly showed MDA reduction and SOD non-significance, suggesting that renal patients may exhibit a more robust enzymatic upregulation due to their higher baseline oxidative burden. In type 2 diabetes patients, Qiu et al. [100] observed a strong reduction in MDA (SMD = -1.29, *p <* 0.0001) and an increase in SOD (MD = 0.59, *p =* 0.006), which aligns with our finding that BMI ≥ 25 and metabolic dysregulation amplify redox responses. For cancer patients, Guedes et al. [101] reported that physical activity significantly reduced oxidative stress markers (SMD = -0.48) and increased antioxidant markers (MD = 1.23), though with high heterogeneity (I² ≈ 81–82%). Our study extends these findings by identifying that the magnitude of response depends not only on disease status but also on BMI, age, and intervention duration—factors that were not consistently explored in the cancer meta-analysis.

#### Meta-analyses with null or mixed findings

Rosado-Pérez et al. [102] examined Tai Chi in adults with various conditions and reported significant increases in SOD (MD = 34.97 U/mL) and CAT (MD = 15.63), along with reductions in lipid peroxidation (MD = -0.02), but found no significant changes in MDA, TAS, or GSH. This stands in contrast to our significant MDA reduction and TAS elevation, suggesting that Tai Chi, as a low-to-moderate intensity modality, may not provide the same redox stimulus as the diverse exercise protocols (including HIIT and resistance training) included in our synthesis. Notably, in people with Down syndrome, Andriolo et al. [103] could not pool data due to insufficient reporting, while Shields et al. [104] found a significant increase in antioxidant enzymes (SMD = 0.39, *p <* 0.05) with low heterogeneity (I² = 15%). This suggests that specific populations with baseline antioxidant deficits may respond more uniformly to exercise, reinforcing the importance of population-specific analysis.

#### Niche and acute exercise meta-analyses

Ferlito et al. [105] examined acute low-load resistance exercise with blood flow restriction (BFR) and found no significant effect on CAT (MD = -0.25, *p =* 0.75) or lipid pro-oxidants (SMD = -0.43, *p =* 0.12). Our acute and chronic pooled analyses did not include BFR as a separate modality; however, our non-significant CAT finding (SMD = -0.61, *p =* 0.428) is consistent with their acute null results, suggesting that enzymatic antioxidant responses are not consistently upregulated in either acute or chronic settings across healthy populations.

#### Heterogeneity and methodological considerations

A critical observation from these comparisons is that nearly all previous meta-analyses reported substantial to extreme heterogeneity across their outcomes (I² ranging from 69% to 97%), indicating that high heterogeneity is not a limitation unique to our study, but rather an inherent characteristic of this research field due to variations in exercise protocols, populations, and biomarker assay methods. While de Sousa et al. [97] achieved low heterogeneity by restricting to a specific biomarker classification, our broader inclusion criteria, which intentionally encompassed both acute and chronic interventions, diverse populations, and multiple assay methods, inevitably generated higher heterogeneity (I² = 87–97%). However, rather than merely reporting this heterogeneity, our study is the first to apply a comprehensive multi-layered analytical hierarchy to explicitly quantify and manage it. This hierarchy included: (i) random-effects models with Hartung-Knapp adjustment to provide confidence intervals; (ii) 95% prediction intervals to estimate the range of true effects in future studies; (iii) pre-specified subgroup analyses to identify effect modifiers (age, BMI, duration); (iv) meta-regression to assess linear moderators; and (v) rigorous publication bias assessments using Egger’s test, Begg’s test, Trim & Fill, and Fail-safe N analyses, complemented by leave-one-out sensitivity analyses. This multi-tiered approach allowed us to move beyond simple yes/no conclusions and provide a nuanced understanding of redox responses across different contexts, while transparently communicating the uncertainty inherent in our pooled estimates—a level of analytical rigor that has not been consistently applied in previous meta-analyses in this field.

#### Unique contribution of the present study

In summary, while previous meta-analyses have demonstrated that exercise reduces oxidative damage and enhances antioxidant capacity, our study is the first to quantitatively synthesize six major redox biomarkers (MDA, SOD, CAT, GSH, TAC, TAS) simultaneously across the full spectrum of human populations, including both healthy and clinical cohorts. We provide novel evidence that: (i) enzymatic antioxidants (SOD and CAT) are not consistently upregulated in humans, in contrast to animal models; (ii) GSH decreases, reflecting increased turnover rather than depletion; (iii) TAS, rather than TAC, is the more sensitive indicator of exercise-induced non-enzymatic enhancement; and (iv) BMI ≥ 25, younger age, and short-term interventions significantly modify redox responses. Methodologically, our study stands out by implementing a pre-registered protocol (PROSPERO), PRISMA 2020 compliance, a comprehensive battery of heterogeneity-handling techniques (including prediction intervals and leave-one-out sensitivity), and a multi-method publication bias assessment (including Trim & Fill and Fail-safe N). These findings refine the simplistic “exercise as a broad-spectrum antioxidant” paradigm and support a hormetic, targeted modulation model that is highly dependent on individual and contextual factors, thereby offering a more precise framework for future research and clinical applications.

### Mechanistic Interpretation

The distinctive MDA↓/TAS↑/GSH↓ biomarker profile is consistent with hormetic redox signaling as a plausible underlying mechanism. In this model, transient exercise-induced reactive oxygen species may act not as indiscriminate toxins but as calibrated signals that potentially activate adaptive pathways, including Nrf2/PGC-1α-mediated antioxidant gene expression, modulation of NADPH oxidase activity, and enhanced GPx/glutathione reductase cycling [106] These mechanistic interpretations are inferential rather than directly demonstrated by our meta-analysis. Our data are derived from circulating biomarkers (MDA, GSH, TAS, SOD, CAT, TAC) and do not include direct measurements of Nrf2 nuclear translocation, PGC-1α expression, SIRT1 activity, or NADPH oxidase function. While the observed biomarker profile is consistent with the activation of these pathways, direct evidence from the included studies was not systematically extracted or analyzed. Therefore, these mechanistic interpretations should be regarded as plausible hypotheses to be tested in future mechanistic studies rather than definitive conclusions. MDA suppression likely reflects multifactorial lipid protection via Nrf2-mediated antioxidant response element activation, improved mitochondrial coupling efficiency, and reduced Nox-derived superoxide production, as evidenced by Parker et al. [37] HIIE-induced systemic lowering of MDA despite localized muscle 4-HNE signaling, which is necessary for insulin sensitization. The glutathione paradox, pooled decrease alongside TAS elevation, precisely matches enhanced GPx-mediated hydrogen peroxide detoxification consuming GSH to produce GSSG, followed by glutathione reductase regeneration elevating non-enzymatic TAS components such as uric acid, bilirubin, and albumin; this dynamic cycling optimizes intracellular redox buffering without net antioxidant depletion [107]. Null enzymatic responses indicate pre-adaptation ceiling effects in frequently active human populations, where SOD and CAT already operate near maximal capacity, compounded by sampling timing discrepancies missing peak Nrf2 activation (typically 2–24 h post-exercise versus conventional 48-hour assessments) [84, 108]. BMI ≥ 25 subgroup amplification (2-fold larger SMDs) arises from elevated baseline Nox2 activity and lipid peroxidation in overweight individuals, creating an expanded hormetic window for exercise signaling, while ≥ 10-week duration effects align with Powers and Jackson’s seminal exercise paradox framework: acute ROS spikes trigger chronic mitochondrial biogenesis, antioxidant gene upregulation, and vascular adaptations explaining physical activity’s 8–13% global mortality reduction [1].

Importantly, these mechanistic interpretations are inferential rather than directly demonstrated by our meta-analysis. Our data are derived from circulating biomarkers (MDA, GSH, TAS, SOD, CAT, TAC) and do not include direct measurements of Nrf2 nuclear translocation, PGC-1α expression, SIRT1 activity, or NADPH oxidase function. While the observed biomarker profile is consistent with the activation of these pathways, direct evidence from the included studies was not systematically extracted or analyzed. Therefore, these mechanistic interpretations should be regarded as plausible hypotheses to be tested in future mechanistic studies rather than definitive conclusions.

### Heterogeneity sources and subgroup analyses

Extreme between-study heterogeneity (I² ranging from 87.5% to 96.51%) was observed across all outcomes, indicating that the majority of the observed variability was attributable to true between-study differences rather than random error. This profound heterogeneity reflects substantial diversity in exercise protocols (acute vs. chronic, aerobic vs. resistance, HIIT vs. moderate-intensity), participant characteristics (healthy vs. diseased, young vs. elderly, sedentary vs. elite athletes), and biomarker assay methods. Under these circumstances, the biological interpretation of pooled effect sizes becomes challenging, as the wide prediction intervals (which frequently crossed the null value) indicate that the true effect in a future study could differ substantially from the pooled estimate. Therefore, we emphasize that greater weight should be placed on the heterogeneity itself and on the prediction intervals rather than on statistical significance alone. Subgroup analyses and meta-regression were employed to explore sources of this heterogeneity, and subgroup-specific findings with reduced heterogeneity (SOD in BMI > 25 subgroups: I² reduced from 87.5% to 17.04%) provide more reliable evidence than overall pooled estimates. The deliberate breadth of our eligibility criteria, while contributing to the observed high heterogeneity, was methodologically justified. This broad scope enabled us to: (i) capture the full range of redox responses to physical activity across the human lifespan and health spectrum, (ii) conduct meaningful subgroup analyses to identify effect modifiers, and (iii) generate hypotheses about which populations and protocols derive the greatest redox benefits. To ensure analytical rigor despite this diversity, we established a clear analytical hierarchy. At the first level, we estimated overall pooled effects to determine whether physical activity exerts any consistent redox modulation. At the second level, we performed subgroup analyses stratified by age, BMI, and intervention duration to identify specific moderators. At the third level, we conducted meta-regression to quantitatively assess the independent contributions of these moderators. This hierarchical approach allowed us to move beyond simple yes/no conclusions and instead provide a nuanced understanding of redox responses across different contexts. Nevertheless, we acknowledge that the extreme heterogeneity limits the precision of our overall estimates, and we have interpreted our findings with appropriate caution, emphasizing subgroup-specific patterns where heterogeneity was substantially reduced (SOD responses in BMI > 25 subgroups showed I² reduced from 87.5% to 17.04%).

Meta-regression analyses confirmed non-linear threshold/saturation kinetics rather than dose-linearity, with no significant age relationships (CAT: β = 0.001, *p =* 0.96; GSH: β=-0.02, *p =* 0.23) but trends toward duration modification (GSH: β=-0.03, *p =* 0.12) and BMI amplification (MDA: β=-0.08, *p =* 0.04). Subgroup Q_between tests (*p <* 0.05) validated effect modification across BMI ≥ 25 (stronger MDA/GSH/TAS responses), intervention duration ≥ 10 weeks (maximal non-enzymatic effects), and age ≥ 50 years (greatest lipid protection), while modality-specific patterns emerged with aerobic training showing largest SMDs versus mixed protocols. Publication bias assessments revealed Egger’s test showed a non-significant trend for CAT (*p =* 0.06), and the evidence for GSH was equivocal (Egger’s *p =* 0.07; Begg’s *p =* 0.01), suggesting that publication bias may not be a major concern for most biomarkers, although cautious interpretation is warranted for GSH given the inconsistency between Egger’s and Begg’s tests. However, fail-safe N analysis estimated 28 null studies were needed to nullify the MDA effect, and leave-one-out sensitivity analyses confirmed robustness, with stable CIs across all biomarkers, no single study disproportionately influenced pooled estimates. Publication bias assessments revealed that, according to our pre-specified threshold (*p <* 0.05), no biomarker showed statistically significant evidence of publication bias based on Egger’s test (CAT: *p =* 0.06; GSH: *p =* 0.07; SOD: *p =* 0.46; MDA: *p =* 0.53; TAC: *p =* 0.81; TAS: *p =* 0.49). Although Begg’s test suggested potential bias for GSH (*p =* 0.01), the inconsistency between Egger’s and Begg’s tests for this biomarker indicates that the evidence for publication bias is equivocal. Overall, the funnel plot symmetry and non-significant Egger’s tests for most outcomes suggest that publication bias is unlikely to have substantially influenced our pooled estimates.

### Biological and translational implications

Biologically, the selective MDA suppression provides mechanistic insight into physical activity’s cardiometabolic protection, as reduced lipid peroxidation minimizes vascular low-density lipoprotein oxidation, preserves endothelial nitric oxide synthase functionality, and attenuates atherogenic inflammation, directly mediating the World Health Organization-documented 8–13% mortality reduction across non-communicable diseases [5]. The TAS elevation and GSH cycling pattern establish enhanced non-enzymatic buffering as the primary human redox adaptation, with clinical implications for precision exercise prescription: optimizing BMI ≥ 25, age ≥ 50 years, and ≥ 10-week moderate-intensity programs to maximize lipid protection, while monitoring elite athletes for Becatti-pattern thiol depletion and overreaching. Translationally, MDA and TAS emerge as superior circulating biomarkers for redox phenotyping and exercise response tracking, supporting their integration into cardiometabolic risk stratification and athletic periodization protocols, while cautioning against overgeneralizing from preclinical enzymatic gains to human contexts.

### Strengths and limitations

This study’s methodological strengths include unprecedented scope (98 studies), PROSPERO registration, PRISMA 2020 compliance, REML random-effects modeling with Hartung-Knapp adjustment, comprehensive subgroup analyses and meta-regression, and confirmed analytical stability through sensitivity and publication bias assessments.

Several important limitations should be acknowledged. The extreme between-study heterogeneity (I² = 87.5–96.51%) reflects substantial variability in exercise protocols, populations, and biomarker assays, precluding definitive causal attribution. Over 50% of RCTs had high risk of bias in missing outcome data and outcome measurement due to inherent blinding challenges. The small number of TAS studies (*n =* 4) reduces statistical precision, and publication bias for GSH and CAT suggests potential overestimation. Reliance on circulating biomarkers provides indirect evidence of tissue-level changes, while small sample sizes (11–40 participants) generated wide prediction intervals. The English-language restriction and unmeasured confounders (nutrition, genetics, pharmacotherapies) may have introduced bias, and non-blinded pre-post designs are susceptible to performance and detection biases. Combining multiple effect sizes from studies with multiple arms or time points may not fully account for statistical dependence, and lack of standardized sampling timing precludes characterization of temporal redox dynamics. Assay heterogeneity across measurement methods contributed to between-study variability, and potential double-counting of participants from overlapping cohorts cannot be entirely excluded. The inclusion of non-exercise therapeutic maneuvers (BPPV repositioning) introduced conceptual heterogeneity, though sensitivity analyses confirmed robustness, and co-intervention confounding (diet, medications, lifestyle) could not be systematically controlled. The substantial clinical and methodological heterogeneity across studies, ranging from healthy young adults and elite athletes to older individuals and clinical populations, and from acute exercise to long-term training, raises uncertainty about whether pooled estimates represent a common biological effect. While we addressed this through subgroup analyses and meta-regression, residual confounding cannot be entirely excluded. The extreme statistical heterogeneity (I² > 90%) across most outcomes, despite appropriate use of random-effects models, substantially limits biological interpretability, and the wide prediction intervals, which frequently crossed the null value, indicate that the true effect in a future study could differ substantially from the pooled estimate. Therefore, greater weight should be placed on heterogeneity itself and on subgroup-specific findings rather than on statistical significance alone. The broad operational definition of physical activity, which included non-conventional interventions such as therapeutic physical maneuvers, may have introduced conceptual heterogeneity and complicated interpretation of pooled findings. Although sensitivity analyses confirmed robustness, this inclusion represents a limitation in terms of intervention homogeneity, as these maneuvers differ from traditional exercise in intensity, energy expenditure, and physiological mechanisms. Leave-one-out sensitivity analyses further demonstrated that the pooled and subgroup estimates were to the exclusion of any single study, although residual heterogeneity limited the precision of some subgroup effects. Finally, we did not perform formal GRADE certainty-of-evidence grading, limiting explicit communication of confidence in the estimates.

### Future directions

Future research must prioritize standardized RCTs employing harmonized 24–48 h post-exercise sampling timing, tissue-specific assays combining plasma markers with skeletal muscle biopsies, and fixed-protocol interventions (12-week moderate aerobic training at 60–75% VO_2_max) to minimize heterogeneity while elucidating temporal redox trajectories. Individual participant data meta-analyses incorporating fitness level, genetic variants (Nrf2 polymorphisms), BMI categories, and sex stratification will identify precise effect modifiers, complemented by mechanistic trials quantifying Nrf2/SIRT1/PGC-1α activation and functional outcomes such as insulin sensitivity and endothelial function. Longitudinal monitoring of elite athletes across competitive seasons using TAS/MDA/thiol tracking will establish overreaching thresholds, while dedicated trials in underrepresented populations, women across reproductive stages, elderly multimorbid patients, and non-elite children/adolescents, will generalize findings beyond current male/athletic-dominant evidence base.

## Conclusion

This registered systematic review and meta‑analysis, synthesizing data from 98 human studies (of which up to 31 were included in the quantitative synthesis) encompassing 4,742 participants, suggests that physical activity may act as a selective modulator of redox homeostasis rather than a broad‑spectrum antioxidant intervention. The pooled estimates indicate that physical activity significantly suppresses lipid peroxidation (MDA: SMD = -1.02, *p =* 0.032) and enhances non‑enzymatic antioxidant buffering capacity (TAS: SMD = 0.76, *p =* 0.001), while exerting a non‑significant trend toward glutathione depletion (GSH: SMD = -0.58, *p =* 0.075) and no significant effects on enzymatic antioxidants (CAT, SOD) or total antioxidant capacity (TAC). These effects were most pronounced in metabolically compromised populations (BMI ≥ 25 kg/m²), interventions lasting ≥ 10 weeks, and individuals aged ≥ 50 years, supporting the concept of physical activity as a potential precision redox modulator that may be tailored to specific population characteristics. Notably, MDA and TAS demonstrated superior utility for clinical monitoring due to their effect sizes, low publication bias, and high fail‑safe N values.

However, these conclusions are tempered by substantial heterogeneity (I² = 87–97%) and wide prediction intervals crossing zero for most biomarkers, indicating considerable uncertainty in the magnitude of individual responses. Sensitivity analyses confirmed robustness for SOD, GSH, and TAS, while MDA, CAT, and TAC were influenced by influential studies, warranting cautious interpretation. Publication bias was detected for GSH (Egger’s *p =* 0.006), and meta‑regression revealed no linear relationships with age or duration, suggesting that non‑linear, threshold‑based, or context‑dependent mechanisms may govern the redox response to exercise.

Therefore, our findings should be interpreted as exploratory and hypothesis‑generating, underscoring the urgent need for future studies with standardized protocols, adequate sample sizes, and consistent reporting of redox biomarkers to confirm these observations, explore non‑linear dose‑response relationships, and establish definitive clinical recommendations for leveraging physical activity as a targeted redox intervention in diverse populations.

## Supplementary Information

Supplementary Material 1.

## Data Availability

All data generated or analysed during this study are included in this published article and its supplementary information files.

## Abbreviations

MDA: Malondialdehyd
SOD: Superoxide dismutase
CAT: Catalase
GSH: Reduced glutathione
TAC: Total antioxidant capacity
TAS: Total antioxidant status
GPx: Glutathione peroxidase
GR: Glutathione reductase
GSSG: Oxidized glutathione
AOPP: Advanced oxidation protein products
ROS: Reactive oxygen species
TBARS: Thiobarbituric acid reactive substances
PC: Protein carbonyls
PRISMA: Preferred reporting items for systematic reviews and meta-analyses
RoB 2: Cochrane risk of bias 2
RCT: Randomized controlled trial
ROBINS-I: Risk Of Bias In Non-randomized Studies – of Interventions
NOS: Newcastle-Ottawa Scale
SMD: Standardized mean difference
CI: Confidence interval
REML: Restricted maximum likelihood
PI: Prediction interval
HIIT: High-intensity interval training
SIE: Sprint interval exercise
CKD: Chronic kidney disease
ESRD: End-stage renal disease
T2DM: Type 2 diabetes mellitus
HFrEF: Heart failure with reduced ejection fraction
BPPV: Benign paroxysmal positional vertigo
PLHIV: People living with HIV
Nrf2: Nuclear factor erythroid 2-related factor 2
PGC-1α: Peroxisome proliferator-activated receptor gamma coactivator 1-alpha
Nox: NADPH oxidase
SIRT1: Sirtuin 1
