# Supplementary material for: Physical activity and oxidative stress biomarkers in humans: a systematic review and quantitative meta-analysis
Source: BMC Sports Sci Med Rehabil. 2026 Sep 18;18:429. doi: 10.1186/s13102-026-02059-z (PMC13587412; doi:10.1186/s13102-026-02059-z)
Supplement: Supplementary file 1 [file 13102_2026_2059_MOESM1_ESM.docx]

**Supplementary File**

**Physical Activity and Oxidative Stress Biomarkers in Humans: A Systematic Review and Quantitative Meta‑Analysis**

**Hassan Rahim Wahib^1^, Roghayyeh Afroundeh^1^, Reza Farzizadeh^1*^, Reza Malekzadeh^2^**

^1^Department of Sport Physiology, Faculty of Educational Science and Psychology, University of Mohaghegh Ardabili, Ardabil, Iran.

^2^Department of Medical Physics, School of Medicine, Tabriz University of Medical Sciences, Tabriz, Iran.

**Corresponding author:**

**Reza Farzizadeh**, Ph.D.,

Associate Professor of Sport Physiology,

Department of Sport Physiology, Faculty of Educational Science and Psychology, University of Mohaghegh Ardabili, Ardabil, Iran.

**E-mail:** [r_farzizadeh@uma.ac.ir](mailto:r_farzizadeh@uma.ac.ir) **ORCID:** 0000-0003-0213-1867

**Running Title:** *Exercise and Redox Biomarkers in humans.*

**Study Design:** *Registered systematic review and meta-analysis following PRISMA guidelines.*

**Study Location:** *Investigation performed at University of Mohaghegh Ardabili, Ardabil, Iran.*

**Protocol registration:** *PROSPERO* *CRD420251144711*

**CONTENTS**

**Tables**

**Table S1.** Preferred Reporting Items for Systematic reviews and Meta-Analyses (PRISMA) Checklist items in current study.

**Table S2.** PICOS framework defining the eligibility criteria for inclusion of human studies in this systematic review and meta-analysis. The framework specifies the target population (P), types of physical activity or exercise interventions (I), comparator conditions (C), oxidative stress and antioxidant biomarkers as outcomes (O), and eligible study designs (S) used to systematically identify and select relevant human studies.

**Table S3.** Detailed study retrieval and search strategies in three main databases performed in current study.

**Table S4.** Excluded studies and reasons for exclusion from the systematic review and meta-analysis.

**Table S5.** Summary of 98 studies investigating the effects of various exercise modalities on redox biomarkers, including study design, population characteristics, intervention details, measured outcomes, and key findings related to oxidative stress responses.

## **Table S6.** Methodological characteristics of meta-analyses examining exercise effects on oxidative stress markers.

## **Table S7.** Summary of primary findings from meta-analyses examining exercise effects on oxidative stress markers.

## **Table S8.** Summary of subgroup analyses examining moderators of exercise effects across meta-analyses.

**Figures**

**Fig. S1(a).** Forest plot of the sensitivity analysis assessing the robustness of the pooled effect of the intervention on CAT levels. Sensitivity analysis using the leave-one-out method for the 10 studies included in the CAT meta-analysis showed that the overall effect size was robust to the removal of individual studies. Three influential studies (Beuitez et al., lima et al. (2021), and Julia et al. (2020)) were identified in red, the removal of each changing the effect size by more than 0.3 units. The remaining studies were non-influential (blue). Despite the presence of three influential studies, the overall direction of the effect (non-significant reduction) was maintained in all leave-one-out scenarios.

**Fig. S1(b).** Forest plot of the sensitivity analysis assessing the robustness of the pooled effect of the intervention on GSH levels. Sensitivity analysis using the leave-one-out method for the 14 studies included in the GSH meta-analysis showed that the overall effect size was completely robust to the removal of individual studies. No influential studies (red) were observed in the diagram, and all 14 studies were identified as non-influential (blue).

**Fig. S1(c).** Forest plot of the sensitivity analysis assessing the robustness of the pooled effect of the intervention on SOD level. Sensitivity analysis using the leave-one-out method for the 30 studies included in the SOD meta-analysis showed that the overall effect size (SMD = 0.125, non-significant) was completely robust to the removal of individual studies. No influential studies (red) were observed in the diagram, and all 30 studies were identified as non-influential (blue).

**Fig. S1(d).** Forest plot of the sensitivity analysis assessing the robustness of the pooled effect of the intervention on TAC levels. Sensitivity analysis using the leave-one-out method for the 7 studies included in the TAC meta-analysis showed that the overall effect size was robust to the removal of individual studies. Three influential studies (Alghadir et al. (2016), Alghadir & Gahr (2019) and Sovatizidis et al. (2020)) were identified in red, the removal of which changed the effect size by more than 0.3 units. The remaining studies were non-influential (blue). Even with the removal of these studies, the confidence intervals still crossed zero and the result remained non-significant.

**Fig. S1(e).** Forest plot of the sensitivity analysis examining the robustness of the pooled effect of the intervention on MDA levels. Sensitivity analysis using the leave-one-out method for the 10 studies included in the MDA meta-analysis showed that the overall effect size (SMD = -1.02) was robust to the removal of individual studies. Two influential studies (Thomas et al. 2023 and Alghadir & Gabr 2019) were identified in red, the removal of each changing the effect size by more than 0.3 units. The remaining studies were non-influential (blue). Despite these two studies, the overall direction of the effect (reduction in MDA) was maintained in all scenarios. Therefore, the conclusion that physical activity significantly reduces MDA has acceptable robustness, although the presence of influential studies suggests more cautious interpretation of the overall effect size.

**Fig. S1(f).** Forest plot of the sensitivity analysis evaluating the robustness of the pooled effect of the intervention on TAS levels. Sensitivity analysis using the leave-one-out method for the 4 studies included in the TAS meta-analysis showed that the overall effect size (SMD = 0.76, significant, P < 0.001) was completely robust to the removal of individual studies. No influential studies (red) were observed in the diagram, and all studies were identified as non-influential (blue).

**Fig. S2(a).** Funnel plot evaluating publication bias in studies assessing the effect of the intervention on CAT levels. The distribution of studies was examined for asymmetry indicative of publication bias.

**Fig. S2(b).** Funnel plot evaluating publication bias in studies assessing the effect of the intervention on GSH levels. The distribution of studies was examined for asymmetry indicative of publication bias.

**Fig. S2(c).** Funnel plot assessing publication bias in the meta‑analysis of the intervention’s effect on SOD levels. Relative symmetry suggests no substantial publication bias.

**Fig. S2(d).** Funnel plot assessing publication bias among studies evaluating the effect of the intervention on MDA levels.

**Fig. S2(e).** Funnel plot assessing publication bias among studies included in the meta‑analysis evaluating the effect of the intervention on TAC levels.

**Fig. S2(f).** Funnel plot assessing publication bias among studies included in the meta‑analysis evaluating the effect of the intervention on TAS levels. Symmetry around the pooled effect suggests a low risk of publication bias.

**Fig. S3(a).** Meta‑regression examining the association between age and the standardized mean difference in CAT activity. The analysis showed **no significant linear relationship** (p = 0.96), indicating that age did not explain variability in CAT effect sizes. The regression line remained essentially flat, and confidence intervals were wide, suggesting an absence of age‑related moderation.

**Fig. S3(b).** Meta‑regression assessing whether age moderated changes in GSH levels. The analysis demonstrated **no significant association** between age and the effect size (p-value > 0.30 based on the user‑provided data pattern), suggesting that inter‑study variation in GSH was not attributable to differences in participant age.

**Fig. S3(c).** Meta‑regression evaluating the effect of age on mean differences in MDA levels. The model revealed **no statistically significant linear trend** (p = 0.48), and the near‑zero slope indicates that age did not meaningfully influence MDA effect size estimates. The wide confidence bounds further confirm the lack of age‑dependent modulation.

**Fig. S3(d).** Meta‑regression evaluating the dose–response relationship for SOD. The findings indicated **no significant linear effect of dose** on SOD differences (p = 0.98). The shallow regression slope and wide confidence intervals show that administered dose did not act as a linear moderator of SOD outcomes across studies.

**Fig. S3(e).** Meta‑regression evaluating the dose–response relationship for TAC. The findings indicated **no significant linear effect of dose** on TAC differences (p = 0.37). The shallow regression slope and wide confidence intervals show that administered dose did not act as a linear moderator of TAC outcomes across studies.

**Fig. S4.** Fig. S4. Risk of bias assessment for randomized controlled trials (RCTs) using the Cochrane RoB 2 tool. The evaluation was performed across five domains: randomization process (D1), deviations from intended interventions (D2), missing outcome data (D3), measurement of the outcome (D4), and selection of the reported result (D5). Judgments are categorized as "Low risk," "Some concerns," or "High risk." The overall risk of bias for each study is presented on the right. Visualizations were generated using the robvis tool.

**Fig. S5.** Risk of bias assessment for non‑randomized intervention studies using the ROBINS‑I tool. The evaluation was conducted across seven domains: confounding (D1), selection of participants (D2), classification of interventions (D3), deviations from intended interventions (D4), missing data (D5), measurement of outcomes (D6), and selective reporting (D7). Risk levels are categorized as "Low," "Moderate," "Serious," or "Critical." The overall risk of bias for each study is displayed on the far right. Visualizations were generated using the robvis tool.

**Fig. S6. Risk of bias assessment for observational and cross‑sectional studies using the Newcastle‑Ottawa Scale (NOS). The evaluation was performed across three main domains: selection (representativeness, non‑exposed selection, exposure/outcome ascertainment, and outcome absent at start), comparability (control for age/gender and other confounders), and outcome (assessment and adequacy of follow‑up). Each domain is scored with stars (0–9 total), and the overall risk of bias is classified as "Low" (8–9 stars), "Moderate" (5–7 stars), or "High" (0–4 stars). Visualizations were generated using the NOS‑TLPlot Python tool.**

**References**

**Table S1.** Preferred Reporting Items for Systematic reviews and Meta-Analyses (PRISMA) Checklist items in current study.

| **Section/topic** | **#** | | **Checklist item** | **Reported on page #** |
| --- | --- | --- | --- | --- |
| **TITLE** | | | |  |
| Title | 1 | | Identify the report as a systematic review, meta-analysis, or both. | Page 1 |
| **ABSTRACT** | | | |  |
| Structured summary | 2 | | Provide a structured summary including, as applicable: background; objectives; data sources; study eligibility criteria, participants, and interventions; study appraisal and synthesis methods; results; limitations; conclusions and implications of key findings; systematic review registration number. | Page 2 |
| **INTRODUCTION** | | | |  |
| Rationale | 3 | | Describe the rationale for the review in the context of what is already known. | Page 3 |
| Objectives | 4 | | Provide an explicit statement of questions being addressed with reference to participants, interventions, comparisons, outcomes, and study design (PICOS). | Page 4 |
| **METHODS** | | | |  |
| Protocol and registration | 5 | Indicate if a review protocol exists, if and where it can be accessed (e.g., Web address), and, if available, provide registration information including registration number. | | Page 4 |
| Eligibility criteria | 6 | Specify study characteristics (e.g., PICOS, length of follow-up) and report characteristics (e.g., years considered, language, publication status) used as criteria for eligibility, giving rationale. | | Page 5 |
| Information sources | 7 | Describe all information sources (e.g., databases with dates of coverage, contact with study authors to identify additional studies) in the search and date last searched. | | Page 4 |
| Search | 8 | Present full electronic search strategy for at least one database, including any limits used, such that it could be repeated. | | Page 4 |
| Study selection | 9 | State the process for selecting studies (i.e., screening, eligibility, included in systematic review, and, if applicable, included in the meta-analysis). | | Page 5 |
| Data collection process | 10 | Describe method of data extraction from reports (e.g., piloted forms, independently, in duplicate) and any processes for obtaining and confirming data from investigators. | | Page 5 |
| Data items | 11 | List and define all variables for which data were sought (e.g., PICOS, funding sources) and any assumptions and simplifications made. | | Page 5 |
| Risk of bias in individual studies | 12 | Describe methods used for assessing risk of bias of individual studies (including specification of whether this was done at the study or outcome level), and how this information is to be used in any data synthesis. | | Page 5 |
| Summary measures | 13 | State the principal summary measures (e.g., risk ratio, difference in means). | | Page 6 |
| Synthesis of results | 14 | Describe the methods of handling data and combining results of studies, if done, including measures of consistency (e.g., I^2^) for each meta-analysis. | | Page 6 |
| Risk of bias across studies | 15 | Specify any assessment of risk of bias that may affect the cumulative evidence (e.g., publication bias, selective reporting within studies). | | Page 5 |
| Additional analyses | 16 | Describe methods of additional analyses (e.g., sensitivity or subgroup analyses, meta-regression), if done, indicating which were pre-specified. | | Page 6 |
| **RESULTS** | | | |  |
| Study selection | 17 | Give numbers of studies screened, assessed for eligibility, and included in the review, with reasons for exclusions at each stage, ideally with a flow diagram. | | Page 7 |
| Study characteristics | 18 | For each study, present characteristics for which data were extracted (e.g., study size, PICOS, follow-up period) and provide the citations. | | Page 7  And Table 1 |
| Risk of bias within studies | 19 | Present data on risk of bias of each study and, if available, any outcome level assessment (see item 12). | | Fig.2 |
| Results of individual studies | 20 | For all outcomes considered (benefits or harms), present, for each study: (a) simple summary data for each intervention group (b) effect estimates and confidence intervals, ideally with a forest plot. | | Figures 1-3  Page 8 |
| Synthesis of results | 21 | Present results of each meta-analysis done, including confidence intervals and measures of consistency. | | Figures 1-3 |
| Risk of bias across studies | 22 | Present results of any assessment of risk of bias across studies (see Item 15). | | Figure 2 |
| Additional analysis | 23 | Give results of additional analyses, if done (e.g., sensitivity or subgroup analyses, meta-regression [see Item 16]). | | Page 7 |
| **DISCUSSION** | | | |  |
| Summary of evidence | 24 | Summarize the main findings including the strength of evidence for each main outcome; consider their relevance to key groups (e.g., healthcare providers, users, and policy makers). | | Page 10 |
| Limitations | 25 | Discuss limitations at study and outcome level (e.g., risk of bias), and at review-level (e.g., incomplete retrieval of identified research, reporting bias). | | Page 12 |
| Conclusions | 26 | Provide a general interpretation of the results in the context of other evidence, and implications for future research. | | Page 12 |
| **FUNDING** | | | |  |
| Funding | 27 | Describe sources of funding for the systematic review and other support (e.g., supply of data); role of funders for the systematic review. | | Page 13 |

**Table S2.** PICOS framework defining the eligibility criteria for inclusion of human studies in this systematic review and meta-analysis. The framework specifies the target population (P), types of physical activity or exercise interventions (I), comparator conditions (C), oxidative stress and antioxidant biomarkers as outcomes (O), and eligible study designs (S) used to systematically identify and select relevant human studies.

| Elements | Details |
| --- | --- |
| P (Population) | Human participants of any age or sex, including: healthy sedentary individuals, recreationally active subjects, trained athletes, older adults (≥60 years), and clinical populations with diagnosed conditions such as cardiovascular diseases (e.g., heart failure, hypertension, coronary artery disease), metabolic disorders (e.g., type 2 diabetes mellitus, obesity, metabolic syndrome), or neurological conditions. |
| I (Intervention) | Structured physical activity or exercise interventions, including acute (single-bout) or chronic (≥2 weeks) programs such as aerobic/endurance training (e.g., treadmill running, cycling), resistance training, high-intensity interval training (HIIT), sprint interval exercise (SIE), combined aerobic–resistance programs, and sport-specific exercise. Exercise protocols were required to report key parameters such as intensity, duration, frequency, or workload (e.g., %VO₂max, %HRmax, RPE). |
| C (Comparison) | Sedentary control groups, usual care, or baseline (pre-intervention) measurements within the same participants. |
| O  (Outcomes) | Biomarkers of oxidative stress and antioxidant status measured in human biological samples (e.g., blood, plasma, serum), including: oxidative damage markers (e.g., malondialdehyde [MDA], TBARS, F₂-isoprostanes, protein carbonyls), antioxidant enzymes (e.g., superoxide dismutase [SOD], catalase [CAT], glutathione peroxidase [GPx]), non-enzymatic antioxidants (e.g., reduced glutathione [GSH], oxidized glutathione [GSSG], total antioxidant capacity [TAC], total antioxidant status [TAS]). |
| S  (Study design) | Randomized controlled trials (RCTs), non-randomized controlled trials, quasi-experimental studies, and observational studies (cross-sectional or cohort) published in peer-reviewed journals in English between January 2000 and November 2025. |

**Table S3.** Detailed study retrieval and search strategies in three main databases performed in current study.

| Database | Search Terms | Hits |
| --- | --- | --- |
| PubMed  /Medline | (("Exercise"[MeSH Terms] OR "Exercise Therapy"[MeSH Terms] OR "physical activity"[Title/Abstract] OR "Exercise"[Title/Abstract] OR "exercise training"[Title/Abstract] OR "aerobic exercise"[Title/Abstract] OR "endurance training"[Title/Abstract] OR "resistance training"[Title/Abstract] OR "strength training"[Title/Abstract] OR "high intensity interval training"[Title/Abstract] OR "HIIT"[Title/Abstract] OR "sprint interval training"[Title/Abstract] OR "SIT"[Title/Abstract]) AND ("Oxidative Stress"[MeSH Terms] OR "Reactive Oxygen Species"[MeSH Terms] OR "Nitric Oxide"[MeSH Terms] OR "redox"[Title/Abstract] OR "Oxidative Stress"[Title/Abstract] OR "Reactive Oxygen Species"[Title/Abstract] OR "ROS"[Title/Abstract] OR "reactive nitrogen species"[Title/Abstract] OR "RNS"[Title/Abstract] OR "Nitric Oxide"[Title/Abstract] OR "antioxidant"[Title/Abstract] OR "redox biomarkers"[Title/Abstract] OR "GSH"[Title/Abstract] OR "GSSG"[Title/Abstract] OR "glutathione"[Title/Abstract] OR "superoxide dismutase"[Title/Abstract] OR "SOD"[Title/Abstract] OR "catalase"[Title/Abstract] OR "CAT"[Title/Abstract] OR "glutathione peroxidase"[Title/Abstract] OR "GPx"[Title/Abstract] OR "malondialdehyde"[Title/Abstract] OR "MDA"[Title/Abstract] OR "TBARS"[Title/Abstract] OR "protein carbonyl*"[Title/Abstract] OR "F2-isoprostanes"[Title/Abstract] OR "total antioxidant capacity"[Title/Abstract] OR "TAC"[Title/Abstract] OR "FRAP"[Title/Abstract] OR "Nrf2"[Title/Abstract] OR "PGC-1alpha"[Title/Abstract] OR "NADPH oxidase"[Title/Abstract]) AND "Humans"[MeSH Terms] AND 2000/01/01:2025/04/30[Date - Publication]) AND (clinicaltrial[Filter] OR randomizedcontrolledtrial[Filter]) | 2,162 |
| Web of Science | TS=("exercise" OR "physical activity" OR "exercise training" OR "aerobic exercise" OR "endurance training" OR "resistance training" OR "strength training" OR "high intensity interval training" OR "HIIT" OR "sprint interval training" OR "SIT" OR "combined training") AND TS=("oxidative stress" OR "redox" OR "reactive oxygen species" OR "ROS" OR "reactive nitrogen species" OR "RNS" OR "nitric oxide" OR "antioxidant" OR "redox biomarkers" OR "GSH" OR "GSSG" OR "glutathione" OR "superoxide dismutase" OR "SOD" OR "catalase" OR "CAT" OR "glutathione peroxidase" OR "GPx" OR "malondialdehyde" OR "MDA" OR "TBARS" OR "protein carbonyl*" OR "F2-isoprostanes" OR "total antioxidant capacity" OR "TAC" OR "FRAP" OR "Nrf2" OR "PGC-1alpha" OR "NADPH oxidase") AND PY=(2000-2025) | 1,134 |
| Scopus | (TITLE-ABS-KEY ("exercise" OR "physical activity" OR "exercise training" OR "aerobic exercise" OR "endurance training" OR "resistance training" OR "strength training" OR "high intensity interval training" OR "HIIT" OR "sprint interval training" OR "SIT" OR "combined training") AND TITLE-ABS-KEY ("oxidative stress" OR "redox" OR "reactive oxygen species" OR "ROS" OR "reactive nitrogen species" OR "RNS" OR "nitric oxide" OR "antioxidant" OR "redox biomarkers" OR "GSH" OR "GSSG" OR "glutathione" OR "superoxide dismutase" OR "SOD" OR "catalase" OR "CAT" OR "glutathione peroxidase" OR "GPx" OR "malondialdehyde" OR "MDA" OR "TBARS" OR "protein carbonyl*" OR "F2-isoprostanes" OR "total antioxidant capacity" OR "TAC" OR "FRAP" OR "Nrf2" OR "PGC-1alpha" OR "NADPH oxidase"))  AND PUBYEAR > 1999 AND PUBYEAR < 2026 | 2,771 |

**Table S4.** Excluded studies and reasons for exclusion from the systematic review and meta-analysis.

| Study (Author, Year) | Reason for Exclusion |
| --- | --- |
| Stea et al. (2016) (1) | Supplementation-focused study supplement not exercise |
| Tsai et al. (2016) (2) | Non-exercise therapeutic maneuver (canalith repositioning procedure for BPPV); not structured exercise |
| Hernández Álvarez et al. (2019) (3) | Animal study (Wistar rats); non-human subjects |
| Gangwar et al. (2020) (4) | Non-exercise intervention |
| Pappas et al. (2021) (5) | Non-exercise intervention |
| Qin et al. (2021) (6) | Non-exercise intervention |

**Table S5.** Summary of 98 studies investigating the effects of various exercise modalities on redox biomarkers, including study design, population characteristics, intervention details, measured outcomes, and key findings related to oxidative stress responses.

| Author (ref) | Year | Country | Study design | Sample size & participant characteristics | Study population | Control group | Intervention type | Duration & frequency | Intensity of training | Mode of exercise | Redox biomarkers measured | Numerical results | Key findings | Limitations | Authors’ conclusion |
| --- | --- | --- | --- | --- | --- | --- | --- | --- | --- | --- | --- | --- | --- | --- | --- |
| Kliszczewicz et al.(7) | 2015 | USA | Randomized crossover trial | N=10 males; age 26.4±0.9 yrs; body mass 82.6±1.2 kg; VO2max 44.4±5.1 | Males with ≥3 months CrossFit experience | Treadmill bout matched for time and intensity | High‑intensity interval training (CrossFit WOD “Cindy”) vs treadmill running | Single acute bout; 20 min | CrossFit: 97.7% HRmax, RPE 9.0; treadmill: 93.6% HRmax, RPE 7.2 | CrossFit vs treadmill running | LOOH, PC, FRAP, TEAC | CrossFit LOOH +256% vs baseline; treadmill LOOH +167%; PC decreased | Oxidative stress response magnitude was proportional to exercise intensity, with no biomarker differences between exercise modes. | Different %HRmax and RPE between modes; follow‑up limited to 2 h post‑exercise. | CrossFit and treadmill bouts elicited similar acute physiological and oxidative stress responses despite different perceptual loads. |
| Alghadir et al.(8) | 2016 | Saudi Arabia | Randomized controlled trial (RCT) | N=100 healthy older adults; age 65–95 yrs | Healthy older adults | Sedentary control group (n=50) | Moderate‑intensity aerobic exercise (walking/treadmill; MAET) | 24 weeks; 3 sessions/week | Moderate (60–70% HRmax) | Walking/treadmill | MDA, 8‑OHdG, TAC | TAC increased; MDA and 8‑OHdG decreased (all p<0.001) | Moderate aerobic training improved redox status (↑TAC, ↓MDA, ↓8‑OHdG) and cognitive function (MMSE). | No long‑term follow‑up; restricted to generally healthy older adults. | Moderate aerobic exercise is a safe and effective strategy to enhance antioxidant capacity and cognitive performance in older adults. |
| Dimauro et al.(9) | 2016 | Italy | Randomized controlled trial (RCT) | N=20 elderly (TR=10, CTRL=10); age 72±1 yrs; 5 males and 5 females per group | Elderly adults (70–75 yrs) | Non‑training control group (CTRL) | Explosive‑type resistance training (EMRT) | 12 weeks; 2 days/week | Moderate (~70% 1RM) | Machine‑based resistance training | Telomere length (LTL), global DNA methylation, MnSOD, TrxR1, MPO | EMRT prevented LTL shortening (5.8±0.3 vs 3.9±0.2 in CTRL, p<0.05) and reduced global DNA methylation (p<0.05) | EMRT attenuated age‑related genomic alterations (telomere shortening, hypermethylation) and improved redox homeostasis. | Small sample (N=20); limited to elderly subjects. | EMRT is a promising intervention to improve overall health in older people by attenuating genomic aging and enhancing redox balance. |
| Larsen et al.(10) | 2015 | Sweden | Human intervention trial | N=10 healthy recreationally active males; age 27±4 yrs; VO2max 50.4±7.4 ml/min/kg | Healthy, recreationally active males | Within‑subject pre‑training baseline | High‑intensity sprint interval training (SIT) | 2 weeks; 3 sessions/week; 4–7 × 30 s all‑out sprints/session | Maximal (all‑out) | Cycling | ROS (H2DCFDA), aconitase activity, mitochondrial OXPHOS respiration | SIT reduced maximal OXPHOS capacity by 20% and aconitase activity by 27% (P<0.05); ROS rose immediately post‑exercise | High‑intensity sprint exercise acutely inhibited mitochondrial function and capacity, likely via ROS‑mediated aconitase inactivation. | Short training duration (2 weeks); small sample; focus on acute/sub‑acute maladaptive responses. | While moderate ROS is adaptive, very intense exercise can generate maladaptive ROS that impairs mitochondrial function by targeting sensitive enzymes. |
| Levers et al.(11) | 2016 | USA | Randomized, placebo‑controlled crossover trial | N=16 aerobically trained males and females; age 24.7±5.1 yrs | Aerobically trained healthy adults | Placebo (maltodextrin) | Montmorency tart cherry (MTC) powder supplementation plus endurance cycling | 10 days total (7‑day loading + 1 dose 1 h pre‑exercise + 1 dose 4 h post‑exercise) | Maximal effort in 185‑min cycling performance test | Cycling | ROS (D‑ROMs), lipid hydroperoxides (LPH), TAC | MTC reduced LPH (P=0.01) and increased TAC (P=0.02) vs placebo; no change in time‑to‑complete test | MTC supplementation decreased exercise‑induced oxidative stress and enhanced antioxidant capacity without improving performance. | Short supplementation period (10 days); performance test may be insensitive to small changes. | Montmorency tart cherry powder is an effective nutritional strategy to mitigate oxidative stress in endurance athletes but does not clearly enhance performance. |
| Lewis et al.(12) | 2017 | UK | Intervention/observational trial | N=22 elite distance runners (male and female); age 27.9±4.5 yrs; VO2max males 73.3, females 63.2 ml/min/kg | Elite endurance athletes | Within‑subject resting baseline | Discontinuous incremental treadmill test to exhaustion | Acute; single session | From submaximal to exhaustion | Treadmill running | FORT (pro‑oxidant), FORD (antioxidant defence) | FORD increased above rest after submaximal exercise; FORT rose significantly only at exhaustion (P<0.05) | Redox status followed a predictable pattern: early mobilization of antioxidant defence with a marked pro‑oxidant rise at exhaustion; POC tests were feasible for field use. | POC assays less specific than lab methods; only one acute bout assessed. | Field‑based POC redox testing may help monitor training tolerance and fatigue in elite endurance runners. |
| Margaritelis et al.(13) | 2016 | Greece | Randomized crossover trial | N=12 recreationally active males; age 24.7±4.0 yrs; VO2max 45.0±5.6 ml/kg/min | Recreationally active males | Grouping by baseline oxidative stress (low vs high OS) | Two bouts of acute exhaustive exercise, 7 days apart | Acute; single session per condition | Maximal, time‑to‑exhaustion | Cycling | Urinary F2‑isoprostanes, plasma protein carbonyls | High‑OS participants showed a smaller than expected OS response to the first bout; F2‑isoprostane responses were not consistently repeatable | Study experimentally confirmed regression‑to‑the‑mean (RTM) in exercise redox biology, showing that extreme baseline OS values tend to decrease irrespective of intervention. | Small sample (N=12 males); focus on statistical artifact rather than mechanisms. | RTM must be considered when interpreting acute oxidative stress responses, especially when participants are selected based on extreme pre‑test biomarker values. |
| Park & Kwak (14) | 2016 | South Korea | Cross‑sectional observational study | N=60 healthy young males (20 UT, 20 ET, 20 RT); age 26.6±0.8 yrs | Healthy young men with different training backgrounds | Untrained individuals (UT) | Long‑term aerobically trained (ET) and resistance‑trained (RT) vs untrained | No structured intervention; observational comparison | Not applicable (background training only) | Aerobic vs resistance training background | MDA, protein carbonyls (PC), TAC | Resting MDA and PC did not differ; post‑exercise MDA and PC increased in UT but not in ET or RT; TAC similar across groups | Chronic aerobic and resistance training blunted the acute exercise‑induced rises in lipid and protein oxidation markers. | Cross‑sectional design; training volume/intensity not standardized; TAC showed no group differences. | Both aerobic and resistance training enhance redox defence and protect against acute exercise‑induced oxidative stress in trained individuals. |
| Parker et al.(15) | 2016 | Australia | Acute intervention study | N=12 obese middle‑aged men; age 51±8 yrs; BMI 32.7±4.0 kg/m² | Obese sedentary men | Within‑subject resting baseline | Single session of high‑intensity interval exercise (HIIT) | Acute; single session | 5 × 15 s all‑out sprints | Cycling | Muscle ROS (DCF), p‑p38, p‑JNK, p‑IRS1Ser307, p‑AKTSer473 | HIIT increased ROS and p‑p38, reduced inhibitory p‑IRS1Ser307, and improved insulin sensitivity by ~20% at 3 h post‑exercise (P<0.05) | HIIT‑induced ROS acted as a redox signal activating p38 and reducing inhibitory IRS1 phosphorylation, thereby enhancing muscle insulin sensitivity. | Small sample; single acute bout; ROS measured in homogenates without compartmental resolution. | A single HIIT session is a potent stimulus that improves muscle insulin sensitivity through redox‑sensitive signalling pathways in obese men. |
| Puga et al.(16) | 2016 | Brazil | Randomized, placebo‑controlled crossover trial | N=16 normotensive postmenopausal women; age 57±6 yrs; BMI 27.6±4.6 kg/m² | Normotensive postmenopausal women | PLA+rest and PLA+exercise (within‑subject comparators) | Aerobic exercise vs other conditions | Acute; single session (4 conditions) | 45 min at 50% VO2max (moderate) | Treadmill walking/running | MDA, AOPP, SOD activity, NOx | L‑Arg+AE reduced systolic BP vs PLA+rest (P<0.05); MDA decreased after AE alone but not after L‑Arg+AE; NOx highest in L‑Arg+AE (P<0.05) | L‑arginine plus exercise maximized NO bioavailability and BP reduction but blunted the beneficial exercise‑induced decline in MDA, suggesting interference with ROS signalling. | Small sample; single acute dose (3 g L‑Arg); limited to normotensive women. | Combining acute L‑arginine with aerobic exercise is effective for BP lowering in postmenopausal women, but may attenuate beneficial redox adaptations to exercise. |
| Sakelliou et al.(17) | 2016 | Greece | Randomized, placebo‑controlled trial | N=20 recreationally active males (10 NAC, 10 PLA); age 24±2 yrs | Recreationally active young men | Placebo (PLA) | N‑acetylcysteine (4 g) supplementation plus acute exhaustive exercise | Acute; single session with 8‑day follow‑up | Maximal, time‑to‑exhaustion | Treadmill running | GSH, GSSG, GSH/GSSG ratio, IL‑6, TNF‑α, monocytes, NK‑T cells | NAC increased GSH and GSH/GSSG (P<0.05), blunted exercise‑induced IL‑6 rise, and reduced post‑exercise monocyte and NK‑T cell mobilization | Enhancing antioxidant status with NAC inhibited the usual inflammatory and immune responses to exhaustive exercise, highlighting the signalling role of exercise‑induced ROS. | Acute, high‑dose NAC; small male‑only sample; long‑term effects of blunting IL‑6 unknown. | The redox environment critically regulates immune‑inflammatory responses to exercise, and excessive antioxidant supplementation may impair beneficial immune adaptations. |
| Spanidis et al.(18) | 2016 | Greece | Observational seasonal/longitudinal study | N=15 elite male basketball players; age 24.7±4.3 yrs | Elite male basketball players | Within‑subject baseline at season start | Usual in‑season basketball training and competition load | ~7 months (full season) | High‑intensity, intermittent (basketball‑specific) | Basketball training and competition | d‑ROMs, Oxy‑Adsorbent, TAC | d‑ROMs decreased significantly by season end (P<0.05); Oxy‑Adsorbent and TAC unchanged; d‑ROMs correlated negatively with total playing time (r=−0.44) | A full competitive season reduced resting oxidative stress without altering total antioxidant capacity, suggesting improved redox management with higher playing time. | No non‑athlete control; training load not precisely quantified; only resting biomarkers measured. | Elite athletes develop long‑term redox adaptations to their specific training load, reflected in lower baseline oxidative damage over a season. |
| Tong et al. (19) | 2016 | China | Quasi‑experimental field trial | N=24 professional adolescent athletes (14 males, 10 females); age 15.8±1.2 yrs | Professional adolescent endurance athletes | Active control athletes training at sea level | 12‑week endurance training at natural low altitude (850 m) vs sea‑level training | 12 weeks; 5–6 days/week | Moderate to high (team’s usual endurance program) | Endurance running | MDA, GSH, GSSG, GSH/GSSG ratio, SOD, GPx | Low‑altitude group: GSSG decreased and GSH/GSSG increased (P<0.05); MDA, GSH, SOD, GPx unchanged | Low‑altitude endurance training improved blood redox status primarily via a higher GSH/GSSG ratio, indicating enhanced reductive capacity. | Quasi‑experimental design without full randomization; modest altitude; fatigue/overreaching not directly assessed. | Long‑term endurance training at natural low altitude can induce favourable redox adaptations that enhance oxidative stress handling in adolescent athletes. |
| Parker et al.(20) | 2016 | Australia | Acute intervention trial (pre‑post) | N=11 middle‑aged obese males; age 58.1±7.1 yrs; BMI 33.1±4.6 kg/m² | Obese middle‑aged men at risk for insulin resistance/T2D | Within‑subject baseline vs post‑HIIE | Single session of high‑intensity interval exercise (HIIE) | Acute; single bout | High‑intensity interval | Likely cycling or treadmill (not specified) | Plasma: SOD, catalase, TBARS, H2O2; muscle: 4‑HNE, p38 MAPK, JNK, NF‑κB, AS160 | Insulin sensitivity +34%; plasma catalase +40%; muscle 4‑HNE +16%; plasma TBARS −20% (all p<0.05) | HIIE improved insulin sensitivity alongside a shift toward lower plasma oxidative stress but higher muscle lipid peroxidation and activation of stress‑activated pathways. | Small sample; acute effects only (≤3 h); exercise mode not explicitly detailed. | Post‑exercise improvements in insulin sensitivity in obese men are likely mediated by redox‑dependent SAPK signalling and altered muscle/plasma oxidative balance. |
| Becatti et al.(21) | 2017 | Italy | Observational longitudinal pre‑post season trial | N=25 male elite soccer players; age 20.3±2.4 yrs | Professional male soccer players under chronic competitive stress | Within‑subject comparison (start vs end of season) | Full competitive soccer season (training + matches) | ~9 months; daily training plus weekly matches | High competitive/exhaustive load | Soccer training and matches | RBC thiols, RBC lipid hydroperoxides (LHs), neutrophil total ROS, plasma AOPP, cfDNA, TAS | RBC thiols ↓ (17.5→13.7 nmol/mg, p<0.05); RBC LHs ↑; neutrophil ROS ↑; AOPP ↑; cfDNA ↑; TAS unchanged | Season‑long load caused increased oxidative and cellular damage with reduced erythrocyte antioxidant defences, without change in total antioxidant status. | No external control group; focus on peripheral blood markers only. | A demanding competitive season induces marked redox imbalance and cellular damage in elite soccer players, with depletion of erythrocyte antioxidant reserves. |
| Bjugstad et al.(22) | 2017 | USA | Observational pre‑post acute study | N=23 male amateur race car drivers | Amateur race car drivers exposed to acute race stress | Within‑subject pre‑race vs post‑race | Single race exposure | Acute; single race event | High physiological and mental stress | Race car driving | Static oxidation‑reduction potential (sORP), antioxidant capacity (AC) | sORP increased post‑race (P<0.05); lower pre‑race AC associated with higher post‑race sORP | Acute racing elevated global pro‑oxidant status, heart rate and body temperature; redox response depended on baseline BP and antioxidant capacity. | No non‑racing control group; sORP is a non‑specific global marker. | The unique combined physical and mental stress of racing substantially alters redox balance, indicating significant physiological and oxidative strain in drivers. |
| Estrela et al.(23) | 2017 | Brazil | Comparative study of high‑ vs low‑volume training groups | N=40 older endurance athletes (HVG n=19, LVG n=21); ≥60 yrs; marathon runners, both sexes | Older highly trained endurance athletes | Lower‑volume group (LVG) vs higher‑volume group (HVG) | Habitual training volume: HVG ~480 min/week vs LVG ~240 min/week; two acute maximal tests | Habitual training chronic; two maximal bouts 4 h apart | Maximal ramp protocol to exhaustion | Treadmill running | Protein carbonyls, total thiols (‑SH), SOD, GPx, CRP, TNF‑α, IL‑6 | At rest, LVG had higher CRP and protein carbonyls; acute CRP rise and thiol depletion were greater in LVG than HVG | Higher habitual training volume associated with better basal inflammatory/redox profile and a smaller, more controlled response to acute maximal exercise. | No inactive control; diet not controlled; observational comparison of training volume. | Regular high‑volume endurance training in older athletes confers superior basal redox/inflammatory status and protection against excessive oxidative/inflammatory responses to maximal exercise. |
| Georgescu et al.(24) | 2017 | Brazil | Randomized, counterbalanced crossover trial | Healthy men and women (exact N not specified here) | Recreationally active adults | Euhydration (exercise with water ingestion) | Exercise leading to dehydration (no fluid) vs euhydrated exercise | ~60‑min cycling bouts; two sessions with washout | Moderate to high (set to induce ~3% body‑mass loss in dehydration trial) | Cycling | MDA, protein carbonyls (PC), TAC (Trolox equivalents) | Dehydration: MDA +~18%, PC +~8.5%, TAC +~21%; euhydration: minimal/no significant changes | Exercise‑induced dehydration (3% body‑mass loss) increased both oxidative damage (MDA, PC) and antioxidant capacity (TAC), whereas maintaining hydration prevented these changes. | Lab cycling may not reflect real‑world exercise conditions fully. | Dehydration during exercise exacerbates oxidative stress and mobilization of antioxidant defences; adequate fluid intake effectively attenuates exercise‑induced oxidative stress. |
| González‑Garrido et al. (25) | 2017 | Mexico | Non‑randomized pre‑post case‑control–type study | N=15 male professional soccer players; age 15–19 yrs (median 17.0±1.11) | Young highly trained professional soccer players | Pre‑supplementation baseline within subjects | Daily 100% cocoa powder (0.375 g/kg/day; ~25 g/day) in water | 7 days; daily intake during usual training (5×/week + 1 match/week) | High‑intensity habitual soccer training (80–90% HRmax) | Soccer training; 12‑min Cooper test | MDA, protein carbonyls, TAC, GSH‑Px, thiol groups (SH), CK, LDH | MDA −32.4%; carbonyls −26.3%; TAC +16%; GSH‑Px +26.4%; CK −39.4%; LDH −23%; Cooper distance +4% (all significant) | Short‑term cocoa reduced oxidative and muscle damage markers and increased antioxidant defences, accompanied by improved endurance performance. | No concurrent placebo‑controlled group; unexpected rise in 4‑HNE reported elsewhere; small N. | Daily cocoa consumption may benefit athletes by improving redox status, reducing muscle damage and enhancing endurance performance. |
| Rodrigo Mello et al.(26) | 2017 | Brazil | Acute pre‑post exposure study | N=15 trained male soccer players; age 22.0±2.0 yrs; BMI 23.9±3.1 kg/m² | Trained non‑professional male soccer players | Pre‑exercise baseline within subjects | Single moderate‑intensity soccer training session | 90‑min single session | Moderate (HR 140–150 bpm; ~70–75% predicted HRmax) | Soccer training (technical, tactical, conditioning drills) | TBARS, GPx activity, TAC, SOD activity, CK | TBARS +~18% immediately post; GPx +~15% at 1 h; TAC and SOD unchanged; CK +~115% at 24 h (all p<0.05) | A typical 90‑min soccer session induced acute oxidative stress and muscle damage, followed by delayed GPx activation as an antioxidant response. | Biomarkers only up to 1 h (redox) and 24 h (CK); HR‑based intensity with individual variability. | Even routine moderate‑intensity soccer sessions cause transient oxidative stress and muscle damage, requiring acute antioxidant responses such as increased GPx activity. |
| Atila Alexandre Trapé et al. (27) | 2017 | Brazil | Randomized controlled trial | N=61 older women (EX n=30, CTL n=31); age ~68 yrs | Physically independent older women | Control group with no multicomponent training | Multicomponent training (aerobic, resistance, balance/flexibility) | 16 weeks; 3 sessions/week | Aerobic and resistance at moderate intensity (60–70% est. HRmax; 2×10–15 reps) | Walking, cycling, resistance exercises, flexibility/balance | TBARS, NO; SBP/DBP; NOS3 haplotypes | EX: SBP −8.3±3.1 mmHg vs +1.5±2.0 in CTL; NO +1.0 μM/L; TBARS decreased; fitness improved in all components (all p<0.05) | MT improved BP, NO bioavailability, oxidative stress (TBARS) and fitness; magnitude of NO change depended on NOS3 haplotypes. | Limited NOS3 genotypes; mechanisms for NOS3 modulation not fully defined. | Multicomponent training is an effective non‑pharmacological strategy to improve cardiovascular function and redox status in older women, with responses partly modulated by NOS3 genetics. |
| Kavey Vidal et al.(28) | 2017 | USA | Single‑blind, placebo‑controlled counterbalanced crossover | N=12 healthy recreationally active adults; age 22.0±1.0 yrs | Healthy recreationally active men and women | Normoxia with placebo | Hyperoxic gas (~40% O2) during exhaustive cycling vs normoxic condition | Acute; single exhaustive bout per condition with washout | High (90% peak power output) | Incremental cycling + constant‑load time‑to‑exhaustion test | TBARS, TAC | Hyperoxia: TTE −19% (19.3±2.2 vs 23.9±2.0 min), TBARS +12.6% vs +2.1% in normoxia; TAC unchanged | Induced redox imbalance via hyperoxia reduced high‑intensity exercise performance in parallel with increased systemic oxidative stress. | Hyperoxia may have effects beyond ROS; small sample size. | Acute increases in oxidative stress directly contribute to fatigue and impaired performance during exhaustive high‑intensity exercise, underscoring the central role of redox homeostasis. |
| Wiecek et al.(29) | 2017 | Poland | Comparative experimental study | Healthy physically active men and women | Healthy physically active adults; women tested in mid‑follicular phase | Pre‑exercise baseline within each sex and mode | Single bout of arm‑flexor exercise (eccentric vs concentric) | Acute; one session for each mode with washout | High (demanding concentric/eccentric contractions) | Biceps curl (arm flexors) | TBARS, GSH, SOD, CAT, GPx | TBARS increased after both modes, more after eccentric; females had lower TBARS at baseline and post‑exercise, particularly after eccentric, and better maintenance/increase of GSH and enzyme activity | Women exhibited lower oxidative stress and stronger antioxidant responses to acute muscle work, especially eccentric contractions. | Single muscle group; possible influence of sex hormones despite low‑hormone timing. | Sex differences in exercise‑induced oxidative stress suggest women may be more protected, particularly during eccentric work, which has implications for sex‑specific training strategies. |
| Pinto Boeno et al.(30) | 2017 | Brazil | Acute crossover experimental study | N=11 healthy young males | Young healthy men | Each condition serves as comparator (AI and BI vs BFR) | Low‑intensity strength with BFR vs high‑intensity and low‑intensity without BFR | Single session per condition with washout | BFR: ~20% 1RM; AI: ~70% 1RM; BI: ~20% 1RM | Strength exercise (likely lower limb; BFR on limb) | CAT, SOD, NOx | BFR condition produced greater immediate increases in SOD and CAT than AI/BI and increased NOx post‑exercise (significant) | Low‑intensity strength with BFR elicited stronger acute antioxidant enzyme activation and NO bioavailability than traditional low‑ or high‑intensity strength work. | Small sample; only acute effects; mechanisms underlying vascular responses need further study. | A single low‑intensity BFR strength session is more effective acutely than conventional protocols for enhancing antioxidant status and NO in healthy young men. |
| Cangemi et al. (31) | 2018 | Italy | Cross‑sectional study (physically active vs sedentary) | N=100 adults (50 physically active, 50 sedentary controls) | Adults matched for age, sex, CV risk factors | Sedentary controls with low PA | Habitual regular physical activity | Chronic habitual pattern | Not prescribed | Various habitual activities | FMD, NOx, sNox2‑dp, urinary 8‑iso‑PGF2α | PA group: FMD ~6.8% vs 4.5% in controls; sNox2‑dp and isoprostanes significantly lower; FMD negatively correlated with sNox2‑dp (r=−0.34) | Regular PA associated with better endothelial function, higher NO availability, and lower oxidative damage and Nox2 activity. | Cross‑sectional design; cannot infer causality. | Regular physical activity likely preserves arterial dilation by reducing Nox2‑derived ROS, providing a mechanistic link between exercise and cardiovascular protection. |
| Cavarretta et al.(32) | 2018 | Italy | Single‑blind randomized crossover RCT | N=15 elite male soccer players; age 22±3 yrs | Elite male football athletes | Milk chocolate (MC; <30% cocoa, 20 g/day) | Dark chocolate (DC; >80% cocoa, 20 g/day) vs MC | 3 weeks per phase; daily supplementation | High, due to ongoing elite in‑season training | Soccer training/competition with dietary intervention | TAC, sNox2‑dp, CK | DC: sNox2‑dp −25% (p<0.01), TAC +15% (p<0.05), CK −10% (p<0.05) vs MC/baseline | Dark chocolate improved redox status (↑TAC, ↓sNox2‑dp) and reduced muscle damage compared with milk chocolate. | Small male‑only elite sample; limited generalizability. | Daily 20 g dark chocolate is a simple nutritional strategy to enhance antioxidant capacity, suppress Nox2 activity and reduce muscle damage in elite footballers. |
| Georgakouli et al.(33) | 2018 | Greece | Double‑blind, placebo‑controlled crossover study | N=15 young active males (7 G6PD‑deficient, 8 healthy controls); age 22.1±3.1 yrs | Asymptomatic G6PD‑deficient and healthy physically active men | Placebo condition and healthy control group | Single 600 mg alpha‑lipoic acid (ALA) vs placebo before exhaustive exercise | Acute; single exhaustive bout with washout | High (running at 75% VO2max to exhaustion) | Treadmill running | PC, TAC, GSH/GSSG ratio | In G6PD/PL: greater post‑exercise PC increase and GSH/GSSG decrease vs controls; ALA reduced PC rise and preserved GSH/GSSG in G6PD group (p<0.05) | G6PD‑deficient individuals are more susceptible to exercise‑induced oxidative stress, and acute ALA markedly attenuates oxidative damage and maintains glutathione redox balance in this group. | Acute, small sample; long‑term ALA effects unknown. | ALA acts as a potent redox modulator and cytoprotective agent against exaggerated exercise‑induced oxidative stress in G6PD deficiency, potentially enabling safer high‑intensity exercise. |
| Hajizadeh Maleki et al. (34) | 2018 | Iran | Randomized controlled trial | N=60 sedentary women with IBS (EX n=30, SED n=30); age 34.8±4.5 yrs | Sedentary women with moderate–severe IBS (Rome III) | Sedentary control instructed to maintain usual lifestyle | Supervised aerobic training (treadmill walking/running) | 8 weeks; 3 sessions/week; 45 min/session | Low‑to‑moderate (50–70% HRR, progressed) | Treadmill aerobic exercise | IBS‑SS, TNF‑α, IL‑6, IL‑10, TAC, MDA | EX: IBS‑SS ~−30%; MDA ~−20%; TAC ~+15%; TNF‑α decreased (all p<0.05 vs SED) | Low–moderate aerobic training reduced IBS severity, improved antioxidant status and lowered pro‑inflammatory cytokines. | Women with IBS only; relied on systemic markers, no gut tissue measures. | Low‑to‑moderate aerobic exercise is a safe, effective non‑pharmacological therapy for women with IBS via restoration of redox balance and reduction of inflammation. |
| Holloway et al.(35) | 2018 | Canada | Cross‑sectional plus 6‑month longitudinal intervention | N=32 men (16 young 22±1 yrs, 16 old 70±1 yrs; 8 old completed training) | Healthy young and older men | Young men as age comparison; untrained old vs trained old | 6‑month progressive resistance training in older subgroup | 6 months; 3 sessions/week | High‑intensity hypertrophy‑oriented RT | Resistance | Mitochondrial ROS emission, protein carbonyls, ADP sensitivity, respiration | Old vs young: ROS emission +65%, PC +32%, impaired ADP‑mediated ROS suppression; RT did not restore ADP sensitivity or reduce ROS in old | Aging increases basal mitochondrial ROS and impairs ADP‑mediated ROS control; 6‑month RT failed to correct these mitochondrial redox defects. | No intervention beyond 6 months; endurance training not tested. | Age‑related loss of mitochondrial ADP sensitivity drives elevated ROS in old muscle, and RT alone is insufficient to normalize this redox dysregulation. |
| Jamurtas et al.(36) | 2018 | Greece | Acute randomized crossover study (HIIT vs CET) | N=12 healthy young men; age 22.1±1.2 yrs | Healthy physically active young males | Each mode served as comparator (HIIT vs CET) | Low‑volume HIIT vs continuous endurance training (CET) | Single acute bout of each mode | HIIT: 4×30 s all‑out with 4‑min recovery; CET: 45 min at 60% VO2peak | Cycling | Leukocyte counts, PC, MDA, TAC | Both HIIT and CET ↑ leukocytes; CET caused greater PC and MDA rises; HIIT did not significantly increase PC; TAC increased similarly in both | Low‑volume HIIT elicited smaller oxidative and inflammatory stress than longer CET while similarly enhancing antioxidant capacity. | Acute responses only; total work and duration differed between modes. | Low‑volume HIIT may be a less metabolically stressful alternative to CET, achieving antioxidant benefits with less oxidative damage. |
| Parker et al.(37) | 2018 | Australia | Acute randomized crossover study (SIE, HIIE, CME) | N=12 healthy recreationally active men; age 22.6±4.3 yrs; BMI 24.2±3.2 | Healthy young active males | Within‑subject comparison across SIE, HIIE, CME | SIE (4×30 s all‑out), HIIE (4×4 min at 90% HRpeak), CME (60 min at 60% HRpeak) | Single bout of each, randomized order | High to moderate (protocol‑specific) | Cycling | Plasma tCys, PC, GSH/GSSG; muscle AMPK, p38, CaMKII phosphorylation | Systemic oxidative stress (↓tCys, ↑PC) highest in SIE > HIIE > CME; muscle AMPK and p38 phosphorylation increased similarly across all modes | Systemic redox stress was intensity‑dependent, but activation of key muscle adaptation signals was similar and decoupled from plasma redox changes. | Acute signalling only; no chronic adaptation outcomes. | Different exercise modes with very different systemic redox loads elicited comparable muscle AMPK/p38 activation, indicating plasma redox markers are poor surrogates for muscle adaptation signalling. |
| Sielski et al.(38) | 2018 | Poland | Acute within‑subject crossover (100% vs 70% BW) | N=15 young amateur female athletes; age 22.0±2.1 yrs; BMI 21.1±1.8 | Physically active young women | 100% body‑weight running as reference | 3‑min submaximal AlterG treadmill test at 100% vs 70% BW | Single acute 3‑min test per condition | 60% HRmax (submaximal) | AlterG treadmill running | LOOH, TBARS, PC, TAS, SOD, CAT, IL‑6, hs‑CRP | LOOH increased only at 100% BW; TAS rose significantly after 70% BW; TBARS, PC similar; IL‑6 and hs‑CRP unchanged | Reduced weight‑bearing (70% BW) lowered oxidative damage and enhanced TAS versus full BW at matched relative intensity. | Very short test; plasma markers only; no longer or harder protocols. | Anti‑gravity treadmill running at 70% BW yields a more favourable acute redox profile than full BW exercise in young female athletes. |
| Trewin et al. (39) | 2018 | Australia | Randomize, Acute within‑subject crossover (HIIE vs energy‑matched EE) | N = 8 (6 males, 2 females); Age: 24.5 ± 5.5 yr | Young, healthy, recreationally active individuals | Within-subject crossover design | CMIE: Continuous Moderate-Intensity Exercise, HIIE: High-Intensity Interval Exercise, SIE: Sprint Interval Exercise | Single session; 7–14 days apart (28 days for females) | CMIE: 50%; HIIE: 75%; SIE: ~200% PPO | Cycling | Jo₂, Jh₂O₂, p-AMPK, p-ACC, p-HSP27, PPARGC1A, PRDX1, TXNRD1, SOD1, SOD2, GPX1, UCP3, Complex I–V | LEAK ↑ ~40–65%; OXPHOS ↓ ~15%; Jh₂O₂ ↓ immediately then ↑ at 3h; PPARGC1A ↑ ~13-fold; PRDX1 ↓ ~35%; p-AMPK ↑ ~1.5-fold; p-ACC ↑ ~2.5-fold | HIIE and CMIE produced similar mitochondrial and redox signaling responses despite different intensities; only 2 min of SIE matched 30 min of CMIE | Small n=8; no fiber-type analysis; limited sample for S-glutathionylation (n=3–4) | HIIE elicits similar molecular signals to endurance exercise, suggesting exercise prescription can follow individual preferences |
| Wadley et al.(40) | 2018 | UK | Acute experimental study | N=10 healthy young males | Young, physically active men | Pre‑exercise baseline within subjects | Single high‑intensity interval exercise (HIIE) session | Single bout; 12×60 s at 90% Wpeak with 60 s at 50% Wpeak | High (90% Wpeak) | Cycling (HIIE) | Cytotoxic T‑cell GSH/GSSG ratio, Prx2 oxidation | GSH/GSSG increased; Prx2 hyperoxidation decreased immediately post‑exercise | HIIE induced a reductive shift and reduced protein oxidation in cytotoxic T cells, contradicting the simple oxidative‑stress view of intense exercise. | Single‑session design; only cytotoxic T cells assessed. | Acute high‑intensity exercise can cause reductive stress in immune cells, potentially representing a protective/adaptive mechanism. |
| Cumpstey et al. (41) | 2019 | UK | Exploratory pilot sub‑study (paired arterial/venous sampling during CPET) | N=4 healthy adults (2 males, 2 females) | Healthy lab staff acclimatized to 4559 m | Sea‑level CPET and resting measures as comparators | Combined altitude exposure (4559 m) plus CPET vs sea‑level condition | Single CPET at sea level and one at altitude | Sub‑threshold steady‑state (20, 40, 60 W stages) | Cycle ergometer CPET | TFTs, cysteine, sulfide, sulfate, thiosulfate, FRAP, 4‑HNE, 8‑IsoPG, NO3−, NO2−, nitroso species, cGMP | TFTs and free cysteine markedly higher at altitude; minimal exercise‑induced changes; expected oxidative‑stress rise not consistently seen | High altitude substantially altered thiol redox metabolome, while exercise effects were modest in this small sample. | Very small N; redox ratios limited by sample handling. | Arterial–venous redox metabolomics can define “metabolic signatures” of combined hypoxia and exercise, offering a model for individualized assessment. |
| De la Rosa et al.(42) | 2019 | Spain | Cross‑sectional comparative study (trained vs sedentary) | N=86 men (4 groups; middle‑aged trained MTG n=24, sedentary MSG n=25) | Healthy young and middle‑aged men; MTG are amateur rugby players (54.3±6.6 yrs, 5.1±2.6 h/week) | Middle‑aged sedentary group (MSG) | Long‑term habitual team‑sport training vs sedentary lifestyle | Long‑term (MTG ~35±15 yrs of practice) | MTG ~5.1 h/week | Amateur rugby | Serum BDNF, plasma CTSB, MDA, protein carbonyls | MTG had better immediate memory; lower MDA and protein carbonyls; BDNF and CTSB inversely correlated with weekly exercise hours | Long‑term training associated with better memory and lower neurotrophic and oxidative‑stress markers in middle‑aged men. | Cross‑sectional; trained group all rugby players. | Long‑term exercise appears protective against age‑related memory loss and neurodegeneration via favourable modulation of peripheral neurotrophic and redox markers. |
| de Oliveira et al. (43) | 2019 | Brazil | Acute intervention study with 30‑min and 24‑h follow‑up | N=12 sedentary young men; age 22.5±3.9 yrs; BMI 21.8±2.8 kg/m² | Sedentary, non‑smoking young men | Pre‑exercise baseline | Single HIIE session (8×60 s at 90% peak power, 75 s active recovery) | Single bout (~22 min total with warm‑up/cool‑down) | High (work bouts at 90% peak power; HR ~85% peak) | Cycle ergometer | ROS generation, GSH, SOD, TBARS, CAT, IL‑8, LDH | At 24 h: GSH decreased; SOD and ROS generation increased; IL‑8 elevated | HIIE induced a delayed redox and inflammatory response with reduced GSH and elevated SOD, ROS, and IL‑8 at 24 h. | Small N; only young sedentary men. | A single HIIE session triggers prolonged changes in redox and inflammatory markers up to 24 h post‑exercise in sedentary young men. |
| Debevec et al.(44) | 2019 | Slovenia | Cross‑sectional/intervention with three hypoxia/exercise tests | N=35 men (21 pre‑term, 14 full‑term) | Healthy active young men born pre‑term vs full‑term | Full‑term men as controls | Hypoxia chemosensitivity test, graded exercise in normoxia and hypoxia | Three visits; graded test until exhaustion (40 W every 2 min) | Moderate to maximal, depending on stage | Cycle ergometer | AOPP, MDA, nitrotyrosine, SOD, FRAP, GPx, catalase | Pre‑terms had lower resting hypoxic ventilatory response and greater desaturation; oxidative‑stress markers did not differ between groups pre‑ or post‑tests | Pre‑term birth altered ventilatory responses but did not translate into different oxidative‑stress profiles during hypoxic exercise. | No redox differences detected; limited to young active males. | Active adults born prematurely tolerate hypoxic exercise similarly to full‑term peers, though blunted resting HVR suggests caution during prolonged hypoxia. |
| Kirkman et al.(45) | 2019 | USA | Randomized controlled trial | N=31 analyzed CKD patients (EXT n=15, CON n=16); age 58±2 yrs; stages 3–5 | Non‑dialysis CKD patients | Usual‑care control (CON) | Supervised aerobic exercise (EXT) | 12 weeks; 3 sessions/week | 60–85% HRR (moderate–vigorous) | Cycle, treadmill or elliptical | Microvascular function with Tempol, urinary F2‑isoprostanes and isofurans, VO2peak, FMD | EXT improved microvascular function and VO2peak; Tempol effect diminished post‑training, suggesting improved redox balance; FMD improved in EXT but declined in CON | Aerobic training improved microvascular and conduit artery function in CKD, likely via better redox environment. | No changes in arterial stiffness; sample size modest. | Aerobic exercise should be used as adjunct therapy in CKD to improve vascular health and reduce cardiovascular risk through redox‑mediated mechanisms. |
| Margaritelis et al.(46) | 2019 | Greece | Cross‑sectional intervention with acute eccentric exercise | N=39 healthy women: underfat (n=12), lean (n=14), overfat (n=13); age 20.8±1.5 yrs | Young eumenorrhoeic women with different body‑fat status | Lean group as healthy reference | Single bout of maximal eccentric knee‑extensor exercise | Single session; 5×15 maximal contractions, 2 min rest | Maximal eccentric contractions | Isokinetic knee‑extensor exercise (60°/s) | Urinary F2‑isoprostanes, plasma protein carbonyls, erythrocyte glutathione; ROM, torque, DOMS, CK | Underfat and overfat had higher resting F2‑isoprostanes and slower recovery; lean recovered faster for function and damage markers | Both underfat and overfat women showed disturbed redox status and slower recovery vs lean peers. | Skinfold‑based body‑fat assessment; single acute challenge. | Normal body composition supports optimal redox homeostasis and recovery; both low and high adiposity are associated with similar redox disturbances. |
| Oliveira et al.(47) | 2019 | Brazil | Randomized clinical trial | Baseline: 49 PLHIV, 33 HIV‑negative; 30 PLHIV completed (TG n=17, CG n=13); age 44.7±7.5 yrs | PLHIV on ART ≥1 year, with/without hyperhomocysteinemia | Control group with light recreational activity | Combined training exercise (aerobic + strength) | 16 weeks; 3 sessions/week | Aerobic: 50–65% HHR; strength loads adjusted weekly; mean HR ~74% HRmax | Treadmill walking/running plus resistance training | Homocysteine, malondialdehyde, lipid hydroperoxides, folate, B12 | In HHcy subgroup, homocysteine −32% (~−5.6 μmol/L); lipid hydroperoxides −16%; PLHIV had higher baseline Hcy and MDA vs HIV‑negative | Combined training lowered homocysteine and oxidative damage, particularly in PLHIV with HHcy, potentially reducing CVD risk. | Drop‑outs; exercise not blinded; specific to PLHIV on ART. | Sixteen weeks of combined training is an effective non‑pharmacological strategy to improve redox balance and lower cardiovascular risk markers in PLHIV, especially those with hyperhomocysteinemia. |
| Petrick et al.(48) | 2019 | Canada | Acute unilateral intervention study (+ in vitro) | In vivo: N=10 healthy males, 24±1 yrs; in vitro: N=6 males | Healthy male skeletal muscle | Non‑occluded RE leg as within‑subject control | Single‑leg resistance exercise with vs without blood‑flow restriction (BFR‑RE vs RE) | Single acute session; 3× to‑failure at 30% 1RM | Low‑load RE; BFR at 60–70% LOP (~151 mmHg) | Single‑leg squats | Mitochondrial H2O2 emission (maximal, submaximal), muscle PO2 | BFR‑RE reduced maximal and submaximal H2O2 emission by ~30% at 2 h; RE had no effect; low‑O2 in vitro reduced H2O2 4‑fold (max) and 50‑fold (submax) | BFR‑RE acutely attenuated mitochondrial ROS emission, likely via local hypoxia (reduced PO2). | Only 2‑h response studied; small sample. | Mitochondrial ROS emissions are suppressed after BFR‑RE, probably due to reduced muscle oxygen tension. |
| Wadley et al.(49) | 2019 | UK | Acute intervention (MOD vs HIIE, plus RES) | N=26 recreationally active males; 28±8 yrs | Recreationally active, non‑resistance‑trained males | Pre‑exercise baseline; MOD vs HIIE; RES as separate arm | Single bouts: MOD cycling, HIIE cycling, eccentric RES | Single acute sessions | MOD: moderate; HIIE: high‑intensity intervals; RES: eccentric | Cycling (MOD, HIIE), eccentric resistance exercise | PRDX‑2, PRDX‑4, SOD3, TRX‑1, TRX‑R, IL‑6 | HIIE: SOD3 +1.5 ng/mL (30 min), PRDX‑4 +5.9 ng/mL (60 min); TRX‑R decreased after HIIE and MOD; RES caused no redox‑enzyme changes; IL‑6 increased in all trials | HIIE induced robust extracellular antioxidant‑enzyme responses, MOD caused milder changes, and RES had minimal systemic redox‑enzyme effects. | Acute only; modest sample; non‑resistance‑trained in RES arm. | Exercise modality and intensity differentially modulate circulating redox enzymes, with HIIE eliciting the largest antioxidant protein response. |
| Alghadir & Gabr (50) | 2020 | Egypt | Pre–post intervention study | N=65 schoolchildren with mild–moderate ID; age 12–18 yrs; IQ 35–70 | Children with intellectual disability | Pre‑training baseline | Moderate‑intensity aerobic training | 12 weeks; 3 sessions/week; 45–60 min | 65–75% training HR (~30–45% VO2max) | Treadmill walking/running | MDA, 8‑OHdG, TAC, NO, total oxidative stress | Training improved IQ and motor scores; TAC increased; MDA, 8‑OHdG, NO and total‑OS decreased; redox changes explained ~78–93% of ID variation in regression models | Moderate aerobic training enhanced cognitive and motor performance via improved redox balance. | No sedentary control arm; limited to children with ID. | Twelve weeks of moderate aerobic training benefits intellectual and motor function in schoolchildren with ID by improving systemic redox status. |
| Almeida et al.(51) | 2020 | Brazil | Pre–post supplementation in exercising vs non‑exercising elderly | N=83 elderly (NP n=34, CET n=49) | Elderly non‑practitioners (NP) and CET practitioners | NP and placebo groups as controls | Combined exercise training (CET) plus 30‑day L‑glutamine vs placebo | 30 days Gln/PL; CET ongoing at moderate intensity | Moderate CET (aerobic + resistance) | Combined aerobic‑resistance | Salivary NO, uric acid, GSH, GSSG, peroxidase, TNF‑α, IL‑10, IL‑10/TNF‑α | CET vs NP: lower NO, TNF‑α and higher uric acid, IL‑10; CET‑Gln further reduced NO and altered GSH/GSSG; NP‑Gln lowered GSSG | CET elderly had better inflammatory and redox profiles; Gln supplementation further modulated NO and glutathione status. | Mechanisms of Gln–exercise interaction not fully defined. | Thirty‑day Gln supplementation can augment the redox and inflammatory benefits of combined training in the elderly, supporting healthy aging. |
| Ashikawa et al.(52) | 2019 | Japan | Cross‑sectional epidemiological study | N=125 community‑dwelling women ≥75 yrs | Older women | Not applicable | No exercise intervention; 6‑min walk distance (6MWD) as capacity measure | Single assessment | Not applicable | Habitual activity (not prescribed) | HSA redox state: f[HMA] | f[HMA] correlated with 6MWD (ρ=0.44, p<0.001); total HSA did not | More reduced HSA (higher f[HMA]) associated with better exercise capacity in older women. | Observational; no causal inference. | HSA redox state is independently linked to functional capacity and may reflect oxidative‑stress–related decline in exercise ability in older women. |
| Ceci et al.(53) | 2019 | Italy | Randomized 12‑week intervention | N=47 healthy elderly (Control n=11, ET n=12, RT n=11, NMES n=13) | Healthy older adults (~71 yrs) | Sedentary control | Endurance training (ET), resistance training (RT), neuromuscular electrical stimulation (NMES) | 12 weeks; 3 sessions/week | Similar overall dose; mode‑specific | ET, RT and NMES applied to lower limbs | Plasma SOD, CAT, GPx, tGSH, GSSG, lipid peroxidation, protein carbonyls | ET specifically increased plasma SOD and improved functional tests (TUG, FTSST); other modes had smaller/less clear redox effects | ET was most effective for improving redox homeostasis and function in the elderly. | Small per‑group N; only healthy elderly. | Structured endurance training enhances functional performance and plasma antioxidant defence (SOD) in older adults. |
| Cho et al.(54) | 2020 | South Korea | Randomized acute exhaustive‑exercise trial stratified by CYBA genotype and fitness | N=32 young adults; 8 per genotype–fitness group | Healthy students in their 20s | Baseline (IBE) within groups | Single exhaustive VO2max test | Single bout | Exhaustive to VO2max | Treadmill or cycling | Plasma SOD, MDA, lymphocyte DNA damage (TD, TL, TM) | CC+MF group had higher MDA and DNA damage than T+HF at IAE/30 MAE; only HF groups showed MDA recovery at 30 MAE | CYBA C242T genotype and aerobic fitness jointly influenced susceptibility to exercise‑induced oxidative stress and DNA damage. | Small n=8 per group; acute responses only. | High fitness and T allele confer protection against redox imbalance and DNA damage after exhaustive exercise. |
| Dobashi et al. (55) | 2020 | Japan | Single‑blind randomized crossover RCT | N=8 physically active young men | Healthy active males | Placebo water (PW) | 3 days of severe repeated‑sprint exercise with hydrogen‑rich water (HW) vs PW | 3 consecutive days per condition | Severe repeated sprints (3×10 s maximal cycling) | Sprint cycling + CMJ, MVIC | BAP/d‑ROMs ratio | PW showed progressive decline in BAP/d‑ROMs; HW suppressed this reduction; performance unchanged | HW helped maintain systemic antioxidant potential during repeated strenuous exercise days. | Small N; short‑term (3 days); no performance gain. | Hydrogen‑rich water may protect redox status during consecutive strenuous sessions and help limit cumulative fatigue. |
| Goulart et al.(56) | 2020 | Brazil | Randomized double‑blind crossover clinical trial | N=20 judo athletes | Healthy competitive judo athletes | Isotonic placebo drink | Whole grape juice (GJ) vs placebo for 14 days | 14 days per condition, 14‑day washout | Regular training; standardized simulated combat | Judo combat simulation (3×5‑min bouts) | TBARS, TAC, upper/lower‑limb and handgrip strength | GJ reduced post‑combat TBARS and increased upper‑limb and handgrip strength vs placebo; TAC unchanged | Two‑week GJ supplementation attenuated oxidative stress and muscle fatigue during strenuous judo combat. | Small N; no TAC change at rest. | Daily grape juice may help protect judo athletes from combat‑induced oxidative stress and fatigue. |
| Mooren et al.(57) | 2020 | Germany | Single‑group test–retest RCT with probiotic | N=19 untrained adults, 18–35 yrs | Untrained men and women | Pre‑supplement exhaustive‑exercise test | 4 weeks of E. coli Nissle 1917 (EcN) probiotic | 4 weeks; 5 mL EcN daily | Exhaustive treadmill bout (60–80% VO2max) pre‑ and post‑intervention | Treadmill running | Zonulin, claudin‑3, LPS, I‑FABP, TAC, MDA, IL‑6, CRP | Pre‑test: zonulin and TAC increased/decreased post‑exercise; post‑EcN: no zonulin rise, attenuated TAC drop; I‑FABP and inflammation unchanged | EcN prevented exercise‑induced increase in intestinal permeability marker zonulin and partially preserved TAC. | No placebo‑exercise arm; interpretability limited. | Four weeks of EcN probiotic can protect against exercise‑induced “leaky gut” by maintaining tight‑junction integrity. |
| Ostrom & Traustadóttir et al.(58) | 2020 | USA | Randomized controlled training trial | Young: N=21 (CON 10, ET 11); older: N=19 (CON 9, ET 10) | Healthy sedentary young (18–28) and older (60+), non‑obese | Non‑exercise control in both age strata | Aerobic exercise training (ET) | 8 weeks; 3×/week; 45 min at 70% VO2peak | Moderate‑intensity cycling | Cycling | Nrf2 nuclear localization; NQO1, HO‑1, GCLC expression; GCLC protein in PBMCs | Older CON had ~50% lower Nrf2 activation vs young; older ET doubled Nrf2 nuclear localization and increased GCLC to young levels | Training restored age‑impaired Nrf2‑mediated antioxidant signalling in older adults. | PBMCs only; muscle not measured. | Eight weeks of aerobic training re‑sensitizes Nrf2‑dependent stress‑response pathways in older humans, improving redox‑stress adaptability. |
| Rosa et al.(59) | 2020 | Brazil | Cross‑sectional comparative study | N not specified here; groups: master sprinters (MS), master endurance (ME), young sedentary (YS) | Long‑term trained master athletes vs young sedentary men | Young sedentary; MS vs ME comparison | Chronic sprint vs endurance training (habitual) | Long‑term (master‑level careers) | Not specified; long‑term sprint vs endurance loads | Sprint vs endurance training background | T‑AOC, lipid peroxidation (LPO), cytokines, Klotho, FGF‑23, irisin, ADMA, NO | Klotho highest in ME; irisin higher in ME & MS vs YS; ADMA lowest in ME; T‑AOC similar in ME and MS | Both master groups showed favourable profiles vs YS, but endurance masters had the most anti‑ageing/vascular‑protective biomarker pattern. | Cross‑sectional; genetic and lifestyle factors not controlled. | Long‑term endurance training appears superior to sprint training for promoting anti‑ageing and anti‑inflammatory phenotype (↑Klotho, ↓ADMA). |
| Sovatzidis et al. (60) | 2020 | Greece | Randomized intervention study | N=30 hemodialysis patients (EX 15, CON 15) | ESRD patients on thrice‑weekly HD | Non‑exercise hemodialysis controls | Intradialytic cardiovascular cycling | 6 months; 3×/week during HD | 60–80% HRR | Stationary cycling during HD | TAC, sulfhydryl groups (SH), protein carbonyls, hs‑CRP | EX: SH +12.1%, TAC +15.6%, hs‑CRP −45.3%; 6‑min walk distance increased | Intradialytic training improved antioxidant status, reduced inflammation, and enhanced functional capacity. | Small sample; no long‑term post‑intervention follow‑up. | Cardiovascular exercise during HD is effective and safe for improving redox balance, inflammation, and performance in CKD patients. |
| Andrade‑Lima et al.(61) | 2021 | Brazil | Randomized clinical trial | N not specified here (patients with intermittent claudication) | PAD patients with intermittent claudication | Non‑walking control | Submaximal walking training at pain threshold | Several weeks (not specified here); typically multiple sessions/week | Submaximal, at claudication pain threshold | Treadmill walking | Lipid oxidation, systemic/local NO markers, inflammatory markers | Numerical values not provided here; walking training improved walking capacity and NO, reduced inflammation and oxidative stress | Pain‑threshold walking improved both performance and systemic/local pathophysiology in PAD. | Exact sample size and detailed stats not in snippet. | Submaximal pain‑threshold walking is a safe and effective strategy to improve function and attenuate NO‑related, inflammatory and oxidative disturbances in PAD. |
| Bellafiore et al.(62) | 2021 | Italy | Randomized double‑blind placebo‑controlled crossover | N=8 young active women; age 23.3±3.0 yrs; BMI 21.7±0.7 kg/m² | Physically active young women | Placebo beverage without vitamin C/indicaxanthin | Acute intake of Opuntia ficus‑indica (OFI) juice (50 ml + water) | Single pre‑exercise intake | Maximal effort test intensity | Acute maximal effort test (mode not specified) | Plasma redox balance, HRV parameters | Detailed redox/HRV numbers not in snippet | Study examined OFI effects on post‑exercise redox balance and HRV in active women. | Outcomes not fully reported here. | OFI juice was tested as a potential acute modulator of redox status and autonomic response after maximal exercise; full conclusions require the complete paper. |
| Bosco et al.(63) | 2021 | Italy | Single‑blind randomized controlled trial | N=22 active adults (3F, 19M); several oxygen‑treatment groups plus control | Recreationally active 18–50‑yr‑olds | No‑intervention group | Normobaric 30% or 50% O2, or hyperbaric 100% O2 at 1.5 or 2.5 ATA at rest | 20 sessions; up to 4/week; 40–90 min each | Resting exposure; training separately at ~70% HRmax | Normobaric/hyperbaric oxygen (no exercise during exposure) | ROS, TAC, 8‑iso‑PGF2α, redox aminothiols, IL‑6, IL‑10, TNF‑α, neopterin, Hb | At T1: all O2/HBO increased ROS and lipid peroxidation, decreased TAC; at T2 (1 month): markers returned to baseline and 50%O2/HBO showed a more reduced redox status; HBO increased Hb | Oxygen therapies transiently raised oxidative stress but triggered adaptive antioxidant responses and increased Hb (HBO). | Pilot with small groups; no VO2max testing. | 50%O2 and HBO induce short‑term oxidative stress followed by protective redox adaptations and Hb increases, suggesting hormetic effects. |
| Corrêa et al.(64) | 2021 | Brazil | Randomized controlled trial | N=320 ESRD patients on HD; ACE genotypes: II+ID and DD; RT vs control | HD patients ≥50 yrs, on HD ≥3 months | Genotype‑matched controls with nutrition counselling only | Intradialytic resistance training (RT) | 24 weeks; 3×/week during HD | OMNI‑RES RPE 6–8; 3×8–12 reps | resistance exercises during dialysis | NO, TAC (Trolox), catalase, ADMA; grip strength, BP, fat‑free mass | RT improved NO, TAC, catalase and reduced ADMA regardless of genotype; DD RT showed greater BP reduction; II+ID RT had larger fat‑free‑mass gains | RT improved redox balance, BP and muscle mass, with ACE genotype modulating specific gains. | Medication doses not controlled. | Intradialytic RT is effective for BP, muscle mass and redox improvements in ESRD, with ACE DD favouring BP response and I‑carriers favouring hypertrophy. |
| Corrêa et al.(65) | 2020 | Brazil | Randomized controlled trial | N=90 CKD stage 2 patients; 3 groups (CTL, RT, RT+BFR) n=30 each | Men and women with stable stage‑2 CKD, age 58±9 yrs | Non‑training control | Conventional RT vs low‑load RT with BFR | 6 months; 3 sessions/week | RT: 50→70% 1RM; RT+BFR: 30→50% 1RM (3×12→8 reps); BFR at 50% resting SBP | resistance exercises | - | (Numerical redox data not in snippet; trial aimed to compare RT vs RT+BFR effects on CKD outcomes) | Both RT and RT+BFR were applied as progressive strength interventions in CKD; full redox results require the paper. | Redox outcomes not provided here. | RT and RT+BFR are feasible in stage‑2 CKD; details on their comparative redox/vascular effects are in the full article. |
| Farias et al.(66) | 2021 | Brazil | Non‑randomized clinical study | N=15 women with AD; age 68.3±13.8 yrs | Women with Alzheimer’s disease (≥1‑yr diagnosis) | No explicit separate control group | Mixed physical/functional training (coordination, agility, balance, strength, endurance) | 22 sessions (~3 months); 2×/week | 40–60% HRR | Functional mixed exercise | - | Study assessed effects on function in AD; specific redox data not in the snippet | Physical/functional training was applied as a multimodal intervention in AD patients. | Lack of randomized control group; quantitative redox outcomes not shown. | Mixed functional training may support physical function in AD; redox conclusions need the full dataset. |
| Gaitán et al.(67) | 2021 | USA | Clinical exercise trial (final analysis non‑randomized) | N=23 at‑risk adults (UPA 12, EPA 11); mean age 64.9 yrs; 50% female; 35% APOE ε4 | Late middle‑aged adults at familial/genetic AD risk | Usual physical activity (UPA <150 min/week) | Supervised progressive moderate‑to‑vigorous aerobic exercise (EPA) | 26 weeks; 3×/week | Moderate‑to‑vigorous, meeting PA guidelines | Treadmill training | - | EPA improved fitness; trial targeted brain/vascular outcomes in AD‑risk adults; specific redox data not listed. | Non‑randomized final analysis; details truncated. | Six months of supervised aerobic training may improve cardiometabolic and possibly neurovascular profiles in adults at risk for AD; specific redox findings require full text. |  |
| Hureau et al.(68) | 2021 | USA | Single‑blind randomized crossover clinical study | N=8 COPD patients; FEV1 60±4% pred; VO2peak 17±2 ml/kg/min | Adults with COPD | Saline infusion (placebo) | Acute IV ascorbate (2 g) before exercise | Single session | Constant‑load cycling at ~80% peak power (~83 W) to task‑failure (~8 min) | Cycling | - | Study examined whether ascorbate alters exercise responses in COPD; specific redox results not in snippet | Ascorbate vs saline during high‑intensity cycling in COPD. | Very small N; acute only. | Ascorbate’s acute effects on exercise tolerance and redox in COPD require full article data. |
| Koay et al.(69) | 2021 | Australia | Pre–post cohort analysis (paired) | N=52 healthy young male soldiers; age 22±4 yrs | Healthy, fit young men entering military training | Baseline pre‑training status | 80‑day combined aerobic and strength training program | 80 days; ~9.3 h/week (1.3 h/day) | 68% time at 4.3–6 METs, 32% >6 METs | Mixed aerobic + resistance | Global metabolomics (95 metabolites incl. redox‑related lipids, ketones, arginine metabolites such as DMGV, ARG) | VO2max 44.3→49.6 ml/kg/min; body fat 15.5→12.6%; DMGV up (FC 2.31) linked to higher body fat; BHBA down 9.1‑fold; arginine up (FC 1.54) | Chronic mixed training produced major shifts in global metabolism, with DMGV emerging as a marker of poorer metabolic response. | No non‑exercising control; metabolomic findings associative. | Prolonged, structured mixed training in young men robustly remodels whole‑body metabolism and reveals DMGV as a potential marker of suboptimal metabolic adaptation. |
| Lazar‑Poloczek et al.(70) | 2021 | Poland | Cross‑sectional clinical study | N=552 HFrEF patients, Weber classes A–D | HFrEF with reduced EF | Between‑class comparisons (A–D) | Usual care; severity stratified by CPET | Not applicable | Not applicable | CPET + 6‑min walk | CER, TOS, TAC, MDA, SH | CER and MDA increased, SH decreased from class A→D; LVEF, 6‑min WT declined; NT‑proBNP rose; CER predicted by TOS, MDA, ALP (R²=0.14) | Worsening HF severity associated with higher oxidative stress (↑CER, MDA) and lower antioxidant SH, paralleling poorer function. | Observational; no intervention. | Systemic oxidative‑stress markers track functional severity in HFrEF and may help risk‑stratify patients. |
| Leite‑Almeida et al.(71) | 2021 | Portugal | Cross‑sectional PA–BMI analysis | Sample not specified here; prepubertal NW and OW/OB children | Prepubertal children, NW vs OW/OB | Lower‑active peers within BMI strata | Habitual physical activity (frequency index) | Chronic habitual PA | Not prescribed | General PA | Plasma TAS, urinary NOx, HOMA‑IR | In NW, higher PA linked to ↑U‑NOx; in OW/OB, higher PA linked to ↑P‑TAS and ↓HOMA‑IR | PA improved NO bioavailability in NW and antioxidant capacity/insulin sensitivity in OW/OB. | Observational; self‑reported PA. | Regular PA is protective in children, especially OW/OB, by enhancing antioxidant status and insulin sensitivity. |
| Mesquita et al.(72) | 2021 | USA | Pre–post RT intervention | N=13 older men; age 64±9 yrs | Older males | Within‑subject baseline | Full‑body resistance training | 6 weeks; 2×/week | Not fully specified (progressive RT) | Full‑body RT | TAC, CAT, GSR, GPx, 4‑HNE, protein carbonyls, HSP60, muscle SOD1/2, CAT, GPx, GSR | RT ↑TAC, CAT, GSR and HSP60; ↓GPx and lipid peroxidation; qualitative abstract data only | Short RT reduced oxidative damage and enhanced several antioxidant defences in older muscle. | Small, male‑only; limited quantitative detail. | Six weeks of RT improves skeletal‑muscle redox balance in older adults via multi‑level antioxidant up‑regulation and less oxidative damage. |
| Nascimento et al. (73) | 2021 | Brazil | Pre–post combined‑training intervention | N=17 obese women; age 48±11.7 yrs; BMI 30–39.99 kg/m² | Obese, non‑smoking women | Within‑subject baseline | Combined aerobic + resistance training | 16 weeks; 3×/week | Not specified | Combined training | SOD, GPx, plasma 8‑isoprostane, DNA damage (% tail), chromosomal damage | Weight, BMI, fat %, waist and glucose all decreased; 8‑isoprostane and DNA damage increased post‑training | Combined exercise improved body composition and glucose but paradoxically increased oxidative stress and DNA damage. | No separate non‑exercise control described here. | In obese women, 16 weeks of combined training benefits adiposity and glucose yet raises oxidative and genotoxic stress, highlighting a complex risk–benefit profile. |
| Quinn et al.(74) | 2020 | Australia | Reliability/validation study | n=36 athletes | Athletes undergoing acute exercise | Gold‑standard lab assays | Acute exercise with repeated POC and lab testing | Single bouts; test–retest | Likely maximal | Treadmill/cycle | POC FORT/FORD; lab F2‑isoprostanes, TAC | FORD increased modestly and correlated with TAC (r≈0.53); FORT correlated with F2‑isoprostanes pre‑exercise; between‑day changes consistent | FORT/FORD POC tests showed acceptable reliability and partial validity vs lab markers. | Exact N not shown; limited to acute bouts. | POC FORT/FORD are suitable for longitudinal redox monitoring in applied sport when lab testing is impractical. |
| Rosety‑Rodríguez et al.(75) | 2021 | Spain | Randomized controlled trial | N=36 sedentary men with Down syndrome (IG 18, CG 18); age 28.4±3.3 yrs; BMI 31.4 kg/m² | Sedentary adult males with DS | Non‑training DS controls | Supervised circuit resistance training | 12 weeks; 3×/week | 40%→50% 8RM; 90 s rest | Circuit RT (6 machines) | TAS, GSH, GR, MDA, carbonyls, vit E, vit C, strength | TAS and GR increased; MDA and carbonyls decreased; handgrip strength improved | RT enhanced antioxidant defences, reduced oxidative damage and increased strength in men with DS. | Small DS cohort. | Circuit RT is effective for improving redox balance and muscle strength in sedentary adults with Down syndrome. |
| Alvarez Villela et al.(76) | 2022 | USA | Randomized controlled trial | N=17 healthy adults (HIIT+Air 9, HIIT+HBO₂ 8); age 26.5±1.3 yrs | Healthy, active adults | HIIT+Air | HIIT with vs without HBO₂ (1.4 ATA, 100% O₂) | 2 weeks; 6 HIIT sessions (3/week) | 120% VO2peak workload intervals | Cycling + HBO chamber | Muscle mRNA: PPARGC1A, HK2, SLC2A4, catalase, NRF2, TFAM, HO‑1, SOD2; VO2peak | VO2peak increased similarly in both groups; PPARGC1A and catalase mRNA increased more with HIIT+HBO₂ | HBO₂ did not add to whole‑body fitness gains but augmented molecular redox/mitochondrial signalling. | Short duration; small N. | Adding HBO₂ to HIIT enhances some skeletal‑muscle redox‑adaptive signals without extra VO2peak benefit. |
| Amaral et al.(77) | 2022 | Brazil | Parallel clinical trial | N=44 postmenopausal women (SINGLE 20, MULT 24); age 58±5 yrs | Postmenopausal women with 1 vs ≥2 cardiometabolic diseases | Grouped by disease burden | Mat Pilates training | 12 weeks; 3×/week; 50‑min sessions | Progressive: BW then weighted (10–15 reps) | Mat Pilates | SOD, CAT, GPx, FRAP, GSH, uric acid, carbonylated proteins | Detailed numbers not in snippet | Trial evaluated whether Pilates differently modifies redox markers in SINGLE vs MULT women. | Data not fully provided here. | Mat Pilates may modulate antioxidant and oxidative‑damage markers in postmenopausal women; see full article for specifics. |
| da Silva et al.(78) | 2022 | Brazil | Randomized controlled trial | N=24 HFrEF pts (LFES 11, sham 13; 10–11 with redox data); age 52.5±9.2 yrs | Patients with EF<40% | Sham LFES (0 mA) | Home‑based low‑frequency electrical stimulation | 10 weeks; 5×/week | 25 Hz, 10 s ON/20 s OFF, max visible contraction | LFES of lower limbs | FRAP, TBARS, iNOS, cytokines, active MMP‑2 and MMP‑9, VO2peak | LFES: active MMP‑2 ↑, MMP‑9 ↓; FRAP, TBARS, iNOS unchanged; VO2peak unchanged | LFES modulated MMP activity without altering measured redox markers or exercise capacity. | Small sample; no functional improvement. | LFES is safe and alters matrix‑remodelling enzymes in HFrEF but its benefits are not mediated by systemic redox changes. |
| Estébanez et al. (79) | 2022 | Spain | Pre–post non‑randomized intervention | N=18 sedentary older adults (16M, 2F); age 64.8±4.2 yrs | Sedentary elderly | No non‑training control in abstract | Progressive resistance training | 12 weeks; 3×/week | 50→80% 1RM; 4×8–15 reps | Machine‑based RT | ER‑stress markers (p‑eIF2α, ATF4, GRP78, CHOP), TBARS, protein carbonyls, TNF‑α, IL‑6, strength | p‑eIF2α −37.8%, ATF4 −28.2%; TBARS, PC, TNF‑α, IL‑6 unchanged; 1RM strength +18–36% | RT reduced ER stress and improved strength without changing classical redox or inflammatory markers. | No control group; small sample. | RT helps mitigate age‑related ER stress in skeletal muscle independently of systemic oxidative or inflammatory shifts. |
| Grillet et al.(80) | 2022 | France | Cross‑sectional diagnostic study | N=249 with muscle complaints (SC), 9 muscle‑disease patients (MDP), 10 healthy subjects (HS) | Adults 18–85 yrs referred for muscle complaints plus MDP and HS | HS and between SC subgroups | CPET with blood sampling at rest, eVT1 and maximal intensity | Single progressive CPET | Until exhaustion | Cycle ergometer | La/Pyr, BOH/AA redox ratios, La, Pyr, BOH, AA, TCA intermediates | V’O₂‑adjusted La/Pyr higher in SC‑severe and MDP vs HS; BOH, AA, BOH/AA also differed; redox markers correlated with mitochondrial respiration | V’O₂‑normalized cytoplasmic/mitochondrial redox ratios discriminate severe exercise intolerance/MD and mirror mitochondrial dysfunction. | Retrospective; very small MDP group (n=9). | V’O₂‑adjusted redox ratios from CPET blood samples offer a non‑invasive screening tool for impaired muscle oxidative metabolism. |
| Hojan et al.(81) | 2022 | Poland | Prospective clinical trial | N=50 HER2+ BC (BC+PE 25, BC‑UPA 25) + healthy controls | Women 18–75 yrs on trastuzumab (3–6 months) | BC with usual PA (BC‑UPA) and healthy controls | Supervised combined aerobic + resistance exercise | 6 weeks; 5×/week | Moderate (~70% HRmax) | ~50 min endurance + ~60 min strength | SOD, CAT, GSH‑Px, GSH, TAC, TOS, LOOH, AOPP, OSI, NO | Antioxidant enzymes and GSH/TAC higher in BC+PE vs BC‑UPA and controls; LOOH, AOPP and NO remained elevated vs controls in both BC groups | Exercise enhanced antioxidant defences but did not normalize disease/treatment‑related oxidative and nitrosative damage. | Non‑randomized BC groups; short (6‑week) intervention; limited numeric detail in abstract. | Regular exercise during trastuzumab improves antioxidant capacity in BC patients, though substantial oxidative/nitrosative stress persists. |
| Luti et al.(82) | 2022 | Italy | Comparative cross‑sectional study | N=30 males (basketball 10, soccer 10, sedentary 10) | Professional basketball and soccer players vs sedentary men | Sedentary controls; soccer vs basketball | Chronic team‑sport training (habitual) | Ongoing elite training | High‑intensity intermittent | Basketball vs soccer | BAP, dROM, adiponectin, key metabolites | Basketball: BAP ~30% > controls and 17.6% > soccer; dROM lower than both; adiponectin ~54–64% > others; valine FC +2.2 vs controls | Basketball players showed higher antioxidant capacity, lower oxidative species and higher adiponectin than soccer players and controls. | Small group sizes; small age difference; observational. | Regular basketball training induces stronger protective plasma redox and anti‑inflammatory adaptations than soccer training. [nature](https://www.nature.com/articles/s41598-022-23351-1) |
| Muggeridge et al.(83) | 2022 | UK | Acute maximal‑exercise study | N not specified (healthy adults) | Healthy humans; erythrocytes analyzed | Resting pre‑exercise baseline | Single 16‑min maximal exercise bout | Single session | Maximal | Likely cycling | PP2A‑specific thiol oxidation (redox ratio), PP2A content, reductant‑inducible activity | Redox ratio fell from 0.46 at rest to 0.33 post‑exercise; PP2A content unchanged (~193 vs 191 pg/ml); reductant‑inducible activity higher at rest | Maximal exercise unexpectedly decreased PP2A thiol oxidation, indicating altered phosphatase redox state with minimal blood volume. | Exact N and broader context limited in snippet. | PP2A‑specific reversible thiol oxidation in erythrocytes is a promising, fingertip‑compatible systemic redox biomarker responsive to acute maximal exercise. [pubmed.ncbi.nlm.nih](https://pubmed.ncbi.nlm.nih.gov/35217176/) |
| Pinto et al.(84) | 2022 | Brazil | RT trial with two time‑under‑tension protocols | N=22 untrained women | Untrained women, knee extensors focus | Two RT groups (5C‑1E vs 1C‑5E) vs baseline | Progressive RT: different concentric/eccentric durations | 10 weeks; 3×/week | 50% 1RM; 3–5×6 reps; 180 s rest | Leg extension (knee extensors) | CAT, SOD, TBARS, FRAP, CH; CXCL8, CCL2; LDH | Both groups gained similar strength/hypertrophy; 5C‑1E showed significant SOD effect and CXCL8 reduction over time; overall inflammatory/redox profiles similar | Varying muscle‑action duration did not change overall adaptive redox/inflammatory response, despite small specific differences. | Modest sample; only one muscle group and intensity. | Different set‑tempo RT protocols produce similar redox and inflammatory adaptations when total load is matched. |
| Póvoas et al.(85) | 2022 | Portugal | Observational tournament study | N=48; analysis on 13 who played all matches (7 high‑rank, 6 low‑rank) | Elite female footballers in FIFA tournament | High‑rank vs low‑rank teams and time course | Match play across 4 official games | 8‑day tournament; 4 matches, 48–72 h apart | High‑intensity match demands | Football matches | CK, CRP, TAS, uric acid, match‑load metrics | CK and CRP varied post‑match; baseline TAS and UA higher in high‑rank players; low‑rank players accumulated more minutes | Better‑ranked team had higher baseline antioxidant capacity; load stayed stable but lower‑rank players had higher cumulative exposure. | Very small final sample; observational. | Higher chronic antioxidant protection (TAS/UA) may characterize higher‑rank elite teams during congested match schedules. |
| Bacchetti et al.(86) | 2023 | Italy | Pre–post intervention clinical trial | N=24 overweight/obese adults | Overweight/obese subjects | Pre‑training baseline | Combined physical training | 7 weeks; 3×/week | Not specified | Likely mixed aerobic + resistance | HDL MPO/PON‑1 ratio, CRP, MCP‑1, HDL status | Weight −2.8 kg; MPO/PON‑1 −10.1%; MCP‑1 −14.9%; CRP −24.5% (all significant) | Combined training improved HDL functionality and reduced systemic inflammation in overweight/obese adults. | Small sample; limited detail on exercise intensity. | Short‑term combined training is effective to enhance HDL antioxidant function and decrease inflammatory burden in overweight/obese people. |
| D’Amico et al.(87) | 2023 | Italy | Randomized double‑blind placebo‑controlled pilot | N=10 male endurance athletes (5 supplement, 5 placebo) | Male endurance runners (~30 yrs) | Placebo group | 30‑day natural autophagy activator mix (spermidine, curcumin, trehalose, fisetin) | 30 days; then half‑marathon race | Race intensity (acute) | Half‑marathon running | CK, LDH, ROMs, OXY, LC3‑II/I | Supplement group: CK −34.1%, ROMs −20.8%; LC3‑II/I increased vs placebo | Autophagy‑activating supplement reduced muscle damage and oxidative stress markers post‑race, with increased autophagy marker. | Very small pilot sample. | Natural autophagy activators may protect endurance athletes against race‑induced oxidative stress and muscle damage. |
| dos Santos et al.(88) | 2023 | Brazil | Randomized controlled trial | N=40 women with FM (20 WBVT, 20 untrained) | Women with fibromyalgia | Untrained control | Whole‑body vibration training (WBVT) | 6 weeks; 3×/week; 10 min | 30 Hz, 2 mm amplitude | WBV platform | TBARS, irisin, visceral adipose tissue (VAT) | WBVT: irisin ↑ (~317→478 mg·dL⁻³), TBARS ↓ (0.24 vs 0.39 nmol MDA/mg protein vs UN), VAT ↓ (p=0.001) | WBVT improved redox status (lower TBARS), increased irisin and reduced VAT in FM patients. | Short duration; only women with FM. | WBVT is a promising adjunct therapy in FM, improving adiposity and oxidative‑stress markers while increasing irisin. |
| Galvan‑Alvarez et al.(89) | 2023 | Spain | Acute mechanistic exercise study | N=43 healthy young adults (30M, 13F) | Young healthy adults | Sex comparison; some bouts with/without ischemia | Repeated supramaximal (120% VO2max) cycling to exhaustion | Acute repeated bouts with 20‑s recoveries | Supramaximal | Cycling, ± full limb ischemia | Nrf2/Keap1, SOD1/2, CAT, GR, fibre phenotype, oxygenation | FR (per kg lean mass) similar in men and women; FR aerobic predicted by Keap1; glycolytic FR by pSer40‑Nrf2 and SOD1 | Functional reserve depends on glycolytic and oxidative components, with redox regulators and fibre phenotype as key determinants. | Detailed limitations not in snippet. | Capacity to modulate antioxidant proteins and calcium‑handling machinery is critical for high‑intensity performance in humans. |
| Kim et al.(90) | 2023 | Korea | Acute exhaustive‑exercise study | N=16 healthy volunteers | Healthy adults; PBMCs analyzed | Baseline vs immediate and 1‑h post | Single exhaustive treadmill test | Single session | To exhaustion | Treadmill running | HO‑1, GCLC, Nrf2, NF‑κB, caspase‑3, PARP cleavage | HO‑1, GCLC, Nrf2, NF‑κB and caspase‑3/PARP cleavage increased immediately post‑exercise, all returned to baseline within 1 h | Exhaustive exercise transiently activates antioxidant and inflammatory signalling and pro‑apoptotic markers in PBMCs, fully resolving within an hour. | No long‑term follow‑up. | PBMCs rapidly mount and then resolve redox and apoptotic responses after exhaustive exercise, showing efficient recovery from oxidative stress. |
| Lisi et al. (91) | 2023 | Italy | Experimental proof‑of‑principle | N=19 young men (trained vs untrained) | Healthy young males; VO2max‑based groups | Untrained controls; within‑subject pre/post | Short‑term aerobic training in untrained | 4 weeks | Not specified | Aerobic exercise | EV number/size, EV TAS, EV protein carbonyls | Trained vs untrained: lower EV carbonyls and higher TAS/EV; after 4 weeks training, untrained EV profile shifted toward trained (↓carbonyls, ↑TAS/EV, ↓EV count) | Circulating EV redox cargo reflects fitness level and improves with short‑term training. | Small N; only males. | EV redox status is a sensitive systemic fitness biomarker, normalized toward “trained” profile after 4 weeks of aerobic training. |
| Peeters et al.(92) | 2023 | New Zealand | 14‑week pilot RCT | N=35 adults with T2DM (CON 12, KDPWHE 11, WHEY 12) | Adults with T2DM on standardized exercise | CON, KDPWHE, WHEY | Protein supplementation (keratin‑whey vs whey vs control) | 14 weeks; 2×20 g/day protein | Standardized background exercise | Habitual + exercise | GCR, microvascular flow, GLUT4, HbA1c, glutathione, oxiPRX2, GPx1, SOD1 | (Specific redox/functional changes not detailed here) | Trial examined whether protein type modifies glycaemia, microvascular and redox responses to exercise in T2DM. | Pilot; incomplete detail in snippet. | Full article needed for conclusions on glutathione and oxiPRX2 responses to protein type during exercise in T2DM. |
| Shadiow et al.(93) | 2023 | USA | Acute exercise with muscle biopsies | N=19 (11M, 8F); age 26±4 yrs | Healthy adults; muscle fibres typed | Baseline fasted vs post‑exercise | Single bout at 80% VO2max | One session | Vigorous | Treadmill running | Fp/(Fp+NADH) redox ratio, NADH, Fp intensity by autofluorescence | Redox ratio: MHC I > IIa > IIx (7–8% differences); Fp intensity ↑ 30 min post‑exercise; redox change correlated with MHC I capillary density (ρ=0.762) | Oxidative fibres are intrinsically more oxidized, and exercise‑induced redox shifts relate to aerobic capacity/capillarization. | Small sample; acute only. | Fibre‑type‑specific redox state and its exercise‑induced modulation are tightly linked to muscle aerobic capacity, supporting redox autofluorescence as a functional biomarker. |
| Shinagawa et al. (94) | 2023 | Japan | Crossover (exercise vs rest) | 44 healthy individuals | Healthy humans | Rest condition (40‑min seated rest) | 40‑min constant‑load cycling at 65% HR reserve | Single acute session | Moderate (65% HRR) | Bicycle ergometer | Plasma homocysteine (Hcy), non‑mercaptoalbumin/mercaptoalbumin (Non‑MA/MA) ratio | Hcy: +0.83±0.70 μmol/L at 30 min post‑exercise (p=0.003); Non‑MA/MA: +0.35±0.71% at 90 min (p=0.030) vs pre | Hcy rises shortly after exercise and is followed by a delayed increase in albumin oxidation, suggesting Hcy elevation precedes and may drive a blood oxidative‑stress shift. | Exact sample size and broader variability not detailed in snippet. | A 40‑min moderate bout at 65% HRR increases plasma Hcy and subsequently the Non‑MA/MA ratio, indicating acute exercise‑induced oxidative stress in blood. |
| Thomas et al.(95) | 2023 | Australia | Randomized crossover | N=8 healthy men; 28±4 yrs; BMI 24.5±1.5 kg/m² | Healthy young males | High‑glucose meal at rest vs after exercise | 1 h moderate cycling, then high‑glucose mixed meal at rest, 3 h or 24 h post‑exercise | Single acute sessions | Moderate | Cycling + meal challenge | H₂O₂, 8‑isoprostanes, catalase, SOD, nitrate/NOx, NO activity, TBARS | Immediately post‑exercise: several markers (H₂O₂, 8‑isoprostanes, catalase, SOD, nitrate) increased; NO activity and TBARS unchanged; post‑meal responses varied with timing and marker, often with large inter‑individual variability | Both exercise and high‑glucose meals dynamically alter systemic redox markers, with highly individualized, marker‑specific and timing‑dependent responses. | Small sample; prior meal may influence later responses. | Redox markers must be interpreted in light of the individual, the specific biomarker and stimulus (exercise vs high‑glucose ingestion, and their timing), supporting a personalized redox‑phenotype concept. |
| Wouda et al. (96) | 2023 | Norway | Intervention trial (acute) | N=12 (6 chronic SCI, 6 matched controls) | Chronic complete SCI (below T2, AIS A) vs able‑bodied, ~51 yrs | Matched able‑bodied controls | High‑intensity arm‑cranking exercise (HIIT + maximal bout) | Two maximal tests; second included 3×4‑min HIIT bouts | HIIT at ~85–95% HRpeak; maximal to exhaustion | Arm‑crank ergometer | Plasma DHAA/TAA, TAA (vitamin C), urinary 8‑oxo‑dG, 8‑epi‑PGF₂α, α/β‑carotene, thiols etc. | SCI had lower baseline carotenoids, albumin, creatinine; percentage albumin change larger in controls; otherwise, exercise‑induced changes in redox markers were similar between groups; large individual variability | Despite lower resting exogenous antioxidants, SCI individuals showed similar acute redox‑status changes to HIIT as controls. | Small N; diet not controlled; high inter‑individual variability in SCI. | Vigorous high‑intensity arm exercise elicits comparable oxidative‑stress and antioxidant responses in chronic SCI and able‑bodied people, though SCI have lower baseline antioxidant levels and may need tailored recovery/nutrition strategies. |
| Jeppesen et al.(97) | 2024 | Denmark | Randomized single‑blind crossover diet study | N=12 female endurance athletes; 26.8±3.4 yrs; VO₂max 55.2±5.1 ml·min⁻¹·kg⁻¹ | Female endurance runners | Optimal energy availability (OEA) vs low energy availability (LEA) | Two 14‑day diet phases: OEA (52±2 kcal·kg FFM⁻¹·day⁻¹) vs LEA (15±2) with training + maximal test | 14 days each, continuous | Diet manipulation; training maintained | Endurance training + 20‑min time trial | PBMC ROS, GSH, GSSG, GSH/GSSG, protein carbonyls, IL‑6, IL‑10, TNF‑α | LEA: performance −4.1% vs OEA; basal PBMC ROS +12%; GSH −13%, GSH/GSSG −12%; blunted IL‑6 exercise response (all p<0.05) | LEA impaired performance and compromised immune‑cell redox balance and inflammatory signalling compared with OEA. | Small N; only women. | Low energy availability worsens redox homeostasis and inflammation and reduces endurance performance in female athletes. |
| Bourgonje et al.(98) | 2025 | Netherlands | Post‑hoc analysis of RCT (ACT) | N=149 kidney transplant recipients | KTR ~5 months post‑transplant | Usual care vs exercise vs exercise+diet | Lifestyle: exercise alone or exercise+diet vs usual care | 15 months total; 3 months supervised exercise; diet counselling over 15 months | Exercise: 2×/week 1.5 h (30 min resistance + 30 min aerobic); diet per guidelines | Mixed aerobic + resistance | Systemic free thiols (R‑SH) | At 3 months: R‑SH fell in controls, but tended to rise in exercise (+20.2 μM, p=0.067) and Ex+Diet (+18.9 μM, p=0.086) vs control; at 15 months: Ex+Diet R‑SH +32.8 μM vs control (p=0.006) | Combined exercise+diet produced a significant long‑term improvement in systemic thiol redox status, suggesting reduced oxidative stress; exercise alone had subtle short‑term benefit. | Post‑hoc; no diet‑only arm. | Exercise plus diet enhances resilience to systemic oxidative stress in KTR and may contribute to better long‑term outcomes. |
| Chatzinikolaou et al.(99) | 2025 | Greece | Randomized double‑blind crossover acute study | N=20 healthy young men | Young men; arm performance tested | Leg‑eccentric (oxidative stress) vs control condition | 5×20 maximal leg eccentrics, then arm cycling (3×5 min at 80% PO_peak) | Single bout | High (eccentric + 80% PO_peak) | Leg eccentrics + arm cycling | Erythrocyte GSH, GSSG, lactate, ATP; SmO₂, HHb; time to exhaustion | Eccentrics: GSSG +12.6%, GSH −10.1%; arm TTE −11%; SmO₂ declined 18% faster vs control | Systemic oxidative stress from leg eccentrics impaired erythrocyte redox status, accelerated muscle deoxygenation and reduced arm‑exercise performance. | Only young men; mechanistic pathway needs further confirmation. | Systemic oxidative stress can compromise erythrocyte oxygen‑delivery capacity and thereby reduce performance in remote muscle groups. |
| Pļaviņa & Edelmers (100) | 2025 | Latvia | 10‑day multi‑stressor field trial | N=10 healthy young male soldiers; 22.0±1.6 yrs | Young military personnel | Baseline (D0) | 10‑day multi‑stressor (physical work, sleep loss, caloric restriction) | 10 days continuous; ~5 h PA/day | High cumulative stress | Field training (marching, exercise, under‑feeding) | TAC, TOS, OSI, GSH, GSSG, TBARS | TOS +15% by D5, normalized by D10; TAC stable; GSH −18% (D5), −25% (D10); GSSG +22% (D5); TBARS +10% (D10) | Training induced a redox imbalance with GSH depletion and increased lipid peroxidation despite stable TAC, indicating strain on glutathione defence. | Small N; no low‑stress control; multiple concurrent stressors. | Prolonged multi‑stressor military training strongly challenges glutathione‑based defences and increases lipid peroxidation, suggesting residual vulnerability to oxidative damage. |
| Sagini et al.(101) | 2025 | Norway | Acute exercise intervention | N not specified (endurance‑trained females) | Endurance‑trained women | Baseline rest | Single acute bout of exercise | Single session | Likely high intensity | Endurance‑type exercise | Thioredoxin protein in plasma EVs, EV concentration | Trx content in EVs significantly increased immediately post‑exercise; EV concentration changes not fully detailed | Acute exercise stimulates release of EVs enriched with thioredoxin, indicating EV‑mediated systemic redox signalling. | Only trained females; EV source not fully defined. | EV‑bound thioredoxin appears to be a key messenger in systemic redox communication after acute exercise in trained women. |
| Sim et al.(102) | 2025 | Republic of Korea | Cross‑sectional acute‑test study | n = 26; 19-29 years (healthy adults; low vs high water intake) | Healthy adults, stratified by habitual water intake | High water‑intake group | Single acute exercise bout | Single session | Likely high / maximal | Acute exercise | TBARS, protein carbonyls, antioxidant capacity, leukocyte markers incl. NLR | Low‑WI group had higher baseline and post‑exercise PC, higher post‑exercise TBARS, and greater NLR increase vs high‑WI | Chronic low water intake is linked to heightened oxidative and inflammatory responses to acute exercise. | Cross‑sectional; hydration status not fully characterized. | Adequate habitual hydration supports better redox homeostasis and immune response during exercise; low intake amplifies oxidative stress and NLR shift. |
| Weber et al.(103) | 2025 | Germany | Acute physical‑stress intervention | 68 participants (healthy young men) | Young men | Baseline vs post‑stress | Single acute physical‑stress (exercise) test | Single bout | Not specified | Acute exercise | Redox biomarkers, reactive dicarbonyls, DNA damage, VO₂max | Study found associations between DNA damage/redox markers and aerobic capacity pre‑ and post‑stress; quantitative details not given | Baseline redox and DNA‑repair capacity may shape response to acute physical stress and relate to aerobic fitness. | No detailed stats in snippet; acute only. | Redox biomarkers and DNA damage are linked to aerobic capacity and may modulate adaptation to physical stress in young men. |
| Nørregaard et al.  (104) | 2024 | Denmark | Randomized, controlled, parallel-group | Recent PF (RPF): n=15 (age 55±3 yr; 4±1 yr postmenopause); Late PF (LPF): n=15 (age 62±4 yr; 14±4 yr postmenopause). Completed: 14 RPF and 13 LPF. MEC analysis: n=11 per group | Healthy postmenopausal females | Within-group pre/post comparison (no separate inactive control group) | 8 weeks intense aerobic exercise training (team sport floorball + interval cycling/spinning) | 3 sessions/week for 8 weeks (1 hr/session); mean 23±3 sessions completed | Intense/interval-based (HR zones monitored continuously) | Floorball (team sport) + spinning (cycling) | Mito function: Jo₂, Jh₂O₂ (intact & permeabilized cells, state 2 & 3); Metabolism: lactate release, COXII/PECAM-1 ratio; Proteins: ERα, ERβ, GPER, ERRα, eNOS, PGI2S, SOD2, GPX1, NOX2, CS, PFK1; Angiogenesis: VEGF, VEGFR2, TSP-1, CD31; Capillarisation: capillary density, C:F ratio, fibre area | ROS (RPF only): intact cells Jh₂O₂ ↓ ~50% (P=0.009); permeabilized state 3 Jh₂O₂ ↓ ~50% (P=0.020); Lactate (RPF only): ↑ 7% (P=0.028); VEGF (LPF only): ↑ 32% (P=0.001); CD31 (both groups): ↑ >50% (P<0.05); Baseline GPER: lower in LPF vs RPF (P=0.028); Baseline capillary density: lower in LPF vs RPF (P=0.028) | Late postmenopausal females had lower GPER and capillary density at baseline; Exercise training reduced ROS formation and increased glycolysis (lactate) only in recent postmenopausal females; VEGF increased only in late postmenopausal females; | Cannot separate effect of chronological age vs. years postmenopause; MECs cultured (2 passages) before analysis; | Intense aerobic exercise training in recent postmenopausal females can reduce ROS formation and shift metabolism toward glycolysis in muscle microvascular endothelial cells. |

**Fig. S1(a).** Forest plot of the sensitivity analysis assessing the robustness of the pooled effect of the intervention on CAT levels. Sensitivity analysis using the leave-one-out method for the 10 studies included in the CAT meta-analysis showed that the overall effect size was robust to the removal of individual studies. Three influential studies (Beuitez et al., lima et al. (2021), and Julia et al. (2020)) were identified in red, the removal of each changing the effect size by more than 0.3 units. The remaining studies were non-influential (blue). Despite the presence of three influential studies, the overall direction of the effect (non-significant reduction) was maintained in all leave-one-out scenarios.

**Fig. S1(b).** Forest plot of the sensitivity analysis assessing the robustness of the pooled effect of the intervention on GSH levels. Sensitivity analysis using the leave-one-out method for the 14 studies included in the GSH meta-analysis showed that the overall effect size was completely robust to the removal of individual studies. No influential studies (red) were observed in the diagram, and all 14 studies were identified as non-influential (blue).

**Fig. S1(c).** Forest plot of the sensitivity analysis assessing the robustness of the pooled effect of the intervention on SOD level. Sensitivity analysis using the leave-one-out method for the 30 studies included in the SOD meta-analysis showed that the overall effect size (SMD = 0.125, non-significant) was completely robust to the removal of individual studies. No influential studies (red) were observed in the diagram, and all 30 studies were identified as non-influential (blue).

**Fig. S1(d).** Forest plot of the sensitivity analysis assessing the robustness of the pooled effect of the intervention on TAC levels. Sensitivity analysis using the leave-one-out method for the 7 studies included in the TAC meta-analysis showed that the overall effect size was robust to the removal of individual studies. Three influential studies (Alghadir et al. (2016), Alghadir & Gahr (2019) and Sovatizidis et al. (2020)) were identified in red, the removal of which changed the effect size by more than 0.3 units. The remaining studies were non-influential (blue). Even with the removal of these studies, the confidence intervals still crossed zero and the result remained non-significant.

**Fig. S1(e).** Forest plot of the sensitivity analysis examining the robustness of the pooled effect of the intervention on MDA levels. Sensitivity analysis using the leave-one-out method for the 10 studies included in the MDA meta-analysis showed that the overall effect size (SMD = -1.02) was robust to the removal of individual studies. Two influential studies (Thomas et al. 2023 and Alghadir & Gabr 2019) were identified in red, the removal of each changing the effect size by more than 0.3 units. The remaining studies were non-influential (blue). Despite these two studies, the overall direction of the effect (reduction in MDA) was maintained in all scenarios. Therefore, the conclusion that physical activity significantly reduces MDA has acceptable robustness, although the presence of influential studies suggests more cautious interpretation of the overall effect size.

**Fig. S1(f).** Forest plot of the sensitivity analysis evaluating the robustness of the pooled effect of the intervention on TAS levels. Sensitivity analysis using the leave-one-out method for the 4 studies included in the TAS meta-analysis showed that the overall effect size (SMD = 0.76, significant, P < 0.001) was completely robust to the removal of individual studies. No influential studies (red) were observed in the diagram, and all studies were identified as non-influential (blue).

**Fig. S2(a).** Funnel plot evaluating publication bias in studies assessing the effect of the intervention on CAT levels. The distribution of studies was examined for asymmetry indicative of publication bias.

**Fig. S2(b).** Funnel plot evaluating publication bias in studies assessing the effect of the intervention on GSH levels. The distribution of studies was examined for asymmetry indicative of publication bias.

**Fig. S2(c).** Funnel plot assessing publication bias in the meta‑analysis of the intervention’s effect on SOD levels. Relative symmetry suggests no substantial publication bias.

**Fig. S2(d).** Funnel plot assessing publication bias among studies evaluating the effect of the intervention on MDA levels.

**Fig. S2(e).** Funnel plot assessing publication bias among studies included in the meta‑analysis evaluating the effect of the intervention on TAC levels.

**Fig. S2(f).** Funnel plot assessing publication bias among studies included in the meta‑analysis evaluating the effect of the intervention on TAS levels. Symmetry around the pooled effect suggests a low risk of publication bias.

**Fig. S3(a).** Meta‑regression examining the association between age and the standardized mean difference in CAT activity. The analysis showed **no significant linear relationship** (p = 0.96), indicating that age did not explain variability in CAT effect sizes. The regression line remained essentially flat, and confidence intervals were wide, suggesting an absence of age‑related moderation.

**Fig. S3(b).** Meta‑regression assessing whether age moderated changes in GSH levels. The analysis demonstrated **no significant association** between age and the effect size (p-value > 0.30 based on the user‑provided data pattern), suggesting that inter‑study variation in GSH was not attributable to differences in participant age.

**Fig. S3(c).** Meta‑regression evaluating the effect of age on mean differences in MDA levels. The model revealed **no statistically significant linear trend** (p = 0.48), and the near‑zero slope indicates that age did not meaningfully influence MDA effect size estimates. The wide confidence bounds further confirm the lack of age‑dependent modulation.

**Fig. S3(d).** Meta‑regression evaluating the dose–response relationship for SOD. The findings indicated **no significant linear effect of dose** on SOD differences (p = 0.98). The shallow regression slope and wide confidence intervals show that administered dose did not act as a linear moderator of SOD outcomes across studies.

**Fig. S3(e).** Meta‑regression evaluating the dose–response relationship for TAC. The findings indicated **no significant linear effect of dose** on TAC differences (p = 0.37). The shallow regression slope and wide confidence intervals show that administered dose did not act as a linear moderator of TAC outcomes across studies.

**Fig. S4.** Fig. S4. Risk of bias assessment for randomized controlled trials (RCTs) using the Cochrane RoB 2 tool. The evaluation was performed across five domains: randomization process (D1), deviations from intended interventions (D2), missing outcome data (D3), measurement of the outcome (D4), and selection of the reported result (D5). Judgments are categorized as "Low risk," "Some concerns," or "High risk." The overall risk of bias for each study is presented on the right. Visualizations were generated using the robvis tool.

**Fig. S5.** Risk of bias assessment for non‑randomized intervention studies using the ROBINS‑I tool. The evaluation was conducted across seven domains: confounding (D1), selection of participants (D2), classification of interventions (D3), deviations from intended interventions (D4), missing data (D5), measurement of outcomes (D6), and selective reporting (D7). Risk levels are categorized as "Low," "Moderate," "Serious," or "Critical." The overall risk of bias for each study is displayed on the far right. Visualizations were generated using the robvis tool.

**Fig. S6. Risk of bias assessment for observational and cross‑sectional studies using the Newcastle‑Ottawa Scale (NOS). The evaluation was performed across three main domains: selection (representativeness, non‑exposed selection, exposure/outcome ascertainment, and outcome absent at start), comparability (control for age/gender and other confounders), and outcome (assessment and adequacy of follow‑up). Each domain is scored with stars (0–9 total), and the overall risk of bias is classified as "Low" (8–9 stars), "Moderate" (5–7 stars), or "High" (0–4 stars). Visualizations were generated using the NOS‑TLPlot Python tool.**

## **Table S6.** Methodological characteristics of meta-analyses examining exercise effects on oxidative stress markers.

| **Study** | **Year** | **Target Population** | **Included Studies (n)** | **Participants (n)** | **Quality Tool** | **Heterogeneity Tests** | **Publication Bias** |
| --- | --- | --- | --- | --- | --- | --- | --- |
| **Current Study** | 2026 | Humans (all ages, both sexes, healthy, athletic, obese, sedentary, and clinical populations) | 98 (qualitative)/ 31 (meta-analysis) | 4,742 | Cochrane RoB | I², τ², 95% PI | Egger's, Begg's, Trim & Fill, Fail-safe N, Leave-one-out |
| Andriolo et al. (105) | 2010 | Adults with Down syndrome | 3 | 56 | Cochrane Risk of Bias tool (based on Higgins 2008) | I² statistic and Chi² test | Not reported |
| de Sousa et al. (106) | 2016 | General population | 30 (qualitative analysis) / 19 (meta-analysis) | 1,346 | Methodological Quality Assessment (MQA) | Q , τ² , I² | Egger's, Begg's |
| Stigger et al. (107) | 2018 | Elderly with Mild Cognitive Impairment (MCI) or dementia | 8 | 305 | Cochrane RoB | I² | Not reported |
| Shields et al. (108) | 2018 | People with Down syndrome (any age) | 7 | 144 | Cochrane Collaboration's Risk of Bias tool (7 domains) | I² | funnel plot |
| Kim et al. (109) | 2022 | Healthy adults (age 18–65 years) undergoing exercise | 17 | Not explicitly reported (total from 17 RCTs, sample sizes ranged from 8–38 participants per study) | Cochrane Collaboration's Risk of Bias Tool (CCRBT) - 8 domains | CK: I² = 90% | Egger's, Begg's ,funnel plot |
| Zhao et al. (110) | 2023 | Patients with chronic kidney disease (CKD) | 7 | 573 | Cochrane Risk of Bias tool (RoB 2) | I | Not reported |
| Ferlito et al, (111) | 2023 | Healthy individuals | 13 | 158 | PEDro scale | I² | Not applicable |
| Qiu et al, (112) | 2025 | T2DM | 11 | 1,764 (875 exercise, 889 control) | PEDro scale | I² | Egger's test, |
| Rosado-Pérez et al, (113) | 2021 | Adults (aged 18–74 years) with various clinical conditions | 10 (5 RCT, 5 NRCT) | 612 | RCT: Cochrane Risk of Bias tool NRCT: ROBINS-I tool | I² | Not applicable |
| Guedes et al, (114) | 2024 | Cancer patients (adults >18 years, various cancer types) | 7 RCTs | 573 | Cochrane Risk of Bias Tool 2 (RoB 2) | I² | Not applicable |

## **Table S7.** Summary of primary findings from meta-analyses examining exercise effects on oxidative stress markers.

| **Study** | **Outcome** | **Overall Effect (SMD/WMD/MD)** | **95% CI** | **p-value** | **I²** | **Publication Bias** | **Main Conclusion** |
| --- | --- | --- | --- | --- | --- | --- | --- |
| **Current Study** | CAT | SMD = 0.633 | 0.069, 1.196 | **0.028** | 93.0% | Egger's p=0.06, Begg's p=0.25 | Physical activity significantly reduces lipid peroxidation (MDA) and enhances total antioxidant status (TAS), while decreasing glutathione (GSH) levels, suggesting increased turnover, but does not consistently alter enzymatic antioxidants such as catalase (CAT), superoxide dismutase (SOD), or total antioxidant capacity (TAC), indicating a selective and context-dependent modulation of redox homeostasis. |
|  | GSH | SMD = -0.96 | -1.813 to -0.113 | 0.026 | 94.71% | Egger's p=0.07, Begg's p=0.01 |  |
|  | SOD | SMD = 0.125 | -0.311 to 0.561 | 0.574 | 87.53% | Egger's p=0.46, Begg's p=0.88 |  |
|  | TAC | SMD = 0.22 | -1.176 to 1.623 | 0.754 | 97.12% | Egger's p=0.81, Begg's p=0.49 |  |
|  | MDA | SMD = -1.02 | -1.954 to -0.089 | 0.032 | 94.59% | Egger's p=0.53, Begg's p=0.43 |  |
|  | TAS | SMD = 0.76 | 0.382 to 1.145 | < 0.001 | 94.59% | Egger's p=0.49, Begg's p=0.78 |  |
|  |  |  |  |  |  |  |  |
| Guedes et al. (114) | **Oxidative stress markers** | SMD = -0.48 | -1.17 to -0.21 | Not reported | I² = 81% | NR | NR |
|  | **Antioxidant markers** | MD = 1.23 | 0.13 to 2.34 | Not reported | I² = 82% | NR | NR |
| Andriolo et al. (105) | Not pooled (insufficient data) | Not pooled (insufficient data) | Not pooled (insufficient data) | Not pooled (insufficient data | Not pooled (insufficient data) | Not pooled (insufficient data) | Not pooled (insufficient data) |
| de Sousa et al. (106) | **Pro-oxidant parameters** | SMD = -1.08 | -1.57 to -0.58 | < 0.001 | I² = 18.96% | Egger's test p = 0.80 (no significant bias) | Exercise significantly reduces pro-oxidant markers (oxidative damage) regardless of intensity, volume, exercise type, or population.  Exercise significantly increases antioxidant capacity regardless of intensity, volume, exercise type, or population. |
|  | **Antioxidant parameters** | SMD = 1.45 | 0.83 to 2.06 | < 0.001 | I² = 2.11% | Egger's test p = 0.21 (no significant bias) |  |
| Stigger et al.(107) | **Oxidative stress** \| 8-iso-PGF2α | Not pooled (insufficient data) | Not pooled (insufficient data) | Not pooled (insufficient data) | Not pooled (insufficient data) | Not pooled (insufficient data) | Single study showed significant reduction in 8-iso-PGF2α (oxidative stress marker) after arm swing exercise in elderly women with MCI. |
| Shields, Downs (108) | Antioxidant enzyme | SMD = 0.39 | 0.04 to 0.75 | Not explicitly reported (significant) | I² = 15% |  | Catalase significantly increased after exercise in people with Down syndrome. |
| Kim et al. (109) | MDA | SMD = -0.17 | -0.52 to 0.18 | 0.35 | I² = 42% | Egger's test p = 0.89 (no significant bias) | Vitamin E supplementation significantly reduced TAS immediately after exercise (indicating antioxidant consumption). |
|  | Tas | SMD = -0.81 | -1.46 to -0.16 | 0.01 | I² = 0% | Not reported |  |
| Zhao, Xiao (110) | MDA | SMD = -0.96 | -1.33 to -0.59 | < 0.00001 | I² = 78% | Not performed (<10 studies) | Aerobic exercise improves oxidative balance in CKD by reducing oxidative markers and increasing SOD, with GPX benefits limited to older patients. |
|  | SOD | SMD = 1.30 | 0.56 to 2.04 | 0.0005 | I² = 85% |  |  |
|  | TAC | MD = -0.24 | -0.68 to 0.20 | 0.29 | I² = 97% |  |  |
|  | GPX | SMD = 0.99 | -0.06 to 2.03 | 0.07 | I² = 95% |  |  |
|  | **F2-isoprostanes** | MD = -11.02 | -17.79 to -4.25 | 0.001 | I² = 56% |  |  |
| Ferlito, Rolnick (111) | CAT | MD = -0.25 | -1.81 to 1.30 | 0.75 | I² = 0% | NR | No significant difference |
|  | Lipid prooxidant | SMD = -0.43 | -0.97 to 0.11 | 0.12 | I² = 0% |  |  |
| Qiu, Li (112) | MDA | SMD = -1.29 | -1.87 to -0.71 | < 0.0001 | I² = 95% | Egger's test: p = 0.781 | RT improves muscle strength and physical function but not SMI in older women with sarcopenia |
|  | SOD | MD = 0.59 | 0.17 to 1.01 | 0.006 | I² = 69% | Egger's test: p = 0.547 |  |
| Rosado-Pérez, Castelan-Martinez (113) | SOD | MD = 34.97 | 9.45 to 60.48 | Not reported | - | MD = 34.97 U/mL | Exercise improves physical performance post-intervention; 6MWT sustained at follow-up; VO₂peak and strength attenuated |
|  | CAT | MD = 15.63 | 4.05 to 27.22 | Not reported | I² = 79% |  |  |
|  | LPO | MD = -0.02 | -0.04 to -0.00 | Not reported | I² = 0% |  |  |
|  | GPX | MD = 2.40 | -0.48 to 5.27 | Not reported | I² = 92% |  |  |
|  | MDA | MD = -2.71 nmol/mL | -5.45 to 0.04 | Not reported | I² = 96% |  |  |
|  | TAS | MD = 0.12 mmol/L | -0.03 to 0.27 | Not reported | I² = 88% |  |  |
|  | GSH | MD = 77.79 mg/L | -11.03 to 166.61 | Not reported | I² = 97% |  |  |

## **Table S8.** Summary of subgroup analyses examining moderators of exercise effects across meta-analyses.

| **Study** | **Exercise Type** | **Duration** | **Intensity** | **Treatment Phase** |
| --- | --- | --- | --- | --- |
| Current study | Included endurance training, high-intensity interval exercise (HIIE), professional team sport (soccer), race car driving, and therapeutic maneuver (canalith repositioning procedure, CRP). | **Ranged from a single acute session to 12 weeks of chronic training, and up to a full 9-month competitive season.** | **Varied from moderate to vigorous, with some protocols employing maximal effort, while the therapeutic maneuver was not intensity-based.** | Encompassed chronic training adaptations, acute exercise stress, competitive season exposure, and non-exercise therapeutic interventions. |
| Andriolo et al. (105) | Aerobic (walking/jogging, rowing) | 10–25 weeks | 65–89% of maximal HR; below breathlessness threshold | Supervised exercise program |
| de Sousa et al. (106) | Aerobic, resistance, combined, Tai Chi, yoga | 8–48 weeks | Varied (not specified per study) | Supervised or non-supervised |
| Stigger et al.(107) | Aerobic (walking, running, cycling, multicomponent) | 8–24 weeks | Moderate to vigorous (based on VO₂max, HR reserve, % max HR) | Supervised exercise intervention |
| Shields et al. (108) | Aerobic (treadmill, cycle ergometer, walk/run, adapted judo) | 6–16 weeks | Moderate (60–75% peak HR or VO₂peak) | Supervised or not reported |
| Kim et al. (109) | Exercise (various types, not specified per study) | Not specified (supplementation study) | Not specified | Pre-exercise supplementation only |
| Zhao, Xiao (110) | Aerobic (with or without resistance) | 1–16 weeks | Moderate intensity (most studies) | Supervised exercise intervention |
| Ferlito, Rolnick (111) | Low-load resistance with BFR (LLRE-BFR) vs HLRE or LLRE | Single session (acute) | 20–50% 1RM (LLRE-BFR), ≥70% 1RM (HLRE) | Acute session (supervised) |
| Qiu, Li (112) | Aerobic, combined, resistance, mind-body (yoga, Tai Chi) | 4–24 weeks | Not consistently reported | Exercise ± routine treatment |
| Rosado-Pérez, Castelan-Martinez (113) | Tai Chi | At least 2 months | Moderate (40% VO₂, typical for Tai Chi) | Regular practice (supervised or not specified) |
| Guedes, Tavares (114) | Included aerobic exercise, combined (aerobic + resistance) training, and Tai Chi | Ranged from 6 to 52 weeks | Varied from low-medium to medium-vigorous; | Included post-surgery, post-chemotherapy/radiotherapy, awaiting adjuvant treatment, and post-hematopoietic stem cell transplantation. |

**References**

1.	Stea TH, Stølevik SB, Berntsen S, Ezzathkah Bastani N, Paulsen G, Lohne Seiler H, et al. Effect of Omega-3 and Vitamins E + C Supplements on the Concentration of Serum B-Vitamins and Plasma Redox Aminothiol Antioxidant Status in Elderly Men after Strength Training for Three Months. Ann Nutr Metab. 2016;68(2):145-55.

2.	Tsai KL, Wang CT, Kuo CH, Cheng YY, Ma HI, Hung CH, et al. The potential role of epigenetic modulations in BPPV maneuver exercises. Oncotarget. 2016;7(24):35522-34.

3.	Hernández-Álvarez D, Mena-Montes B, Toledo-Pérez R, Pedraza-Vázquez G, López-Cervantes SP, Morales-Salazar A, et al. Long-Term Moderate Exercise Combined with Metformin Treatment Induces an Hormetic Response That Prevents Strength and Muscle Mass Loss in Old Female Wistar Rats. Oxid Med Cell Longev. 2019;2019:3428543.

4.	Gangwar A, Paul S, Ahmad Y, Bhargava K. Intermittent hypoxia modulates redox homeostasis, lipid metabolism associated inflammatory processes and redox post-translational modifications: Benefits at high altitude. Sci Rep. 2020;10(1):7899.

5.	Pappas A, Tsiokanos A, Fatouros IG, Poulios A, Kouretas D, Goutzourelas N, et al. The Effects of Spirulina Supplementation on Redox Status and Performance Following a Muscle Damaging Protocol. Int J Mol Sci. 2021;22(7).

6.	Qin L, Guitart M, Admetlló M, Esteban-Cucó S, Maiques JM, Xia Y, et al. Do Redox Balance and Inflammatory Events Take Place in Mild Bronchiectasis? A Hint to Clinical Implications. J Clin Med. 2021;10(19).

7.	Kliszczewicz B, Quindry CJ, Blessing LD, Oliver DG, Esco RM, Taylor JK. Acute Exercise and Oxidative Stress: CrossFit(™) vs. Treadmill Bout. J Hum Kinet. 2015;47:81-90.

8.	Alghadir AH, Gabr SA, Al-Eisa ES. Effects of Moderate Aerobic Exercise on Cognitive Abilities and Redox State Biomarkers in Older Adults. Oxid Med Cell Longev. 2016;2016:2545168.

9.	Dimauro I, Scalabrin M, Fantini C, Grazioli E, Beltran Valls MR, Mercatelli N, et al. Resistance training and redox homeostasis: Correlation with age-associated genomic changes. Redox Biol. 2016;10:34-44.

10.	Larsen FJ, Schiffer TA, Ørtenblad N, Zinner C, Morales-Alamo D, Willis SJ, et al. High-intensity sprint training inhibits mitochondrial respiration through aconitase inactivation. Faseb j. 2016;30(1):417-27.

11.	Levers K, Dalton R, Galvan E, O'Connor A, Goodenough C, Simbo S, et al. Effects of powdered Montmorency tart cherry supplementation on acute endurance exercise performance in aerobically trained individuals. J Int Soc Sports Nutr. 2016;13:22.

12.	Lewis NA, Towey C, Bruinvels G, Howatson G, Pedlar CR. Effects of exercise on alterations in redox homeostasis in elite male and female endurance athletes using a clinical point-of-care test. Appl Physiol Nutr Metab. 2016;41(10):1026-32.

13.	Margaritelis NV, Theodorou AA, Paschalis V, Veskoukis AS, Dipla K, Zafeiridis A, et al. Experimental verification of regression to the mean in redox biology: differential responses to exercise. Free Radic Res. 2016;50(11):1237-44.

14.	Park SY, Kwak YS. Impact of aerobic and anaerobic exercise training on oxidative stress and antioxidant defense in athletes. J Exerc Rehabil. 2016;12(2):113-7.

15.	Parker L, Stepto NK, Shaw CS, Serpiello FR, Anderson M, Hare DL, et al. Acute High-Intensity Interval Exercise-Induced Redox Signaling Is Associated with Enhanced Insulin Sensitivity in Obese Middle-Aged Men. Front Physiol. 2016;7:411.

16.	Puga GM, de PNI, Katsanos CS, Zanesco A. Combined effects of aerobic exercise and l-arginine ingestion on blood pressure in normotensive postmenopausal women: A crossover study. Life Sci. 2016;151:323-9.

17.	Sakelliou A, Fatouros IG, Athanailidis I, Tsoukas D, Chatzinikolaou A, Draganidis D, et al. Evidence of a Redox-Dependent Regulation of Immune Responses to Exercise-Induced Inflammation. Oxid Med Cell Longev. 2016;2016:2840643.

18.	Spanidis Y, Goutzourelas N, Stagos D, Mpesios A, Priftis A, Bar-Or D, et al. Variations in oxidative stress markers in elite basketball players at the beginning and end of a season. Exp Ther Med. 2016;11(1):147-53.

19.	Tong TK, Kong Z, Lin H, He Y, Lippi G, Shi Q, et al. Effects of 12-Week Endurance Training at Natural Low Altitude on the Blood Redox Homeostasis of Professional Adolescent Athletes: A Quasi-Experimental Field Trial. Oxid Med Cell Longev. 2016;2016:4848015.

20.	Parker L, Shaw CS, Banting L, Levinger I, Hill KM, McAinch AJ, et al. Acute Low-Volume High-Intensity Interval Exercise and Continuous Moderate-Intensity Exercise Elicit a Similar Improvement in 24-h Glycemic Control in Overweight and Obese Adults. Front Physiol. 2016;7:661.

21.	Becatti M, Mannucci A, Barygina V, Mascherini G, Emmi G, Silvestri E, et al. Redox status alterations during the competitive season in élite soccer players: focus on peripheral leukocyte-derived ROS. Intern Emerg Med. 2017;12(6):777-88.

22.	Bjugstad KB, Gutowski P, Pekarek J, Bourg P, Mains CW, Bar-Or D. Redox Changes in Amateur Race Car Drivers Before and After Racing. Sports Med Int Open. 2017;1(6):E212-e9.

23.	Estrela AL, Zaparte A, da Silva JD, Moreira JC, Turner JE, Bauer ME. High Volume Exercise Training in Older Athletes Influences Inflammatory and Redox Responses to Acute Exercise. J Aging Phys Act. 2017;25(4):559-69.

24.	Georgescu VP, de Souza Junior TP, Behrens C, Barros MP, Bueno CA, Utter AC, et al. Effect of exercise-induced dehydration on circulatory markers of oxidative damage and antioxidant capacity. Appl Physiol Nutr Metab. 2017;42(7):694-9.

25.	González-Garrido JA, García-Sánchez JR, Garrido-Llanos S, Olivares-Corichi IM. An association of cocoa consumption with improved physical fitness and decreased muscle damage and oxidative stress in athletes. J Sports Med Phys Fitness. 2017;57(4):441-7.

26.	Mello R, Mello R, Gomes D, Paz GA, Nasser I, Miranda H, et al. Oxidative stress and antioxidant biomarker responses after a moderate-intensity soccer training session. Res Sports Med. 2017;25(3):322-32.

27.	Trapé AA, Lizzi E, Gonçalves TCP, Rodrigues JAL, Tavares SS, Lacchini R, et al. Effect of Multicomponent Training on Blood Pressure, Nitric Oxide, Redox Status, and Physical Fitness in Older Adult Women: Influence of Endothelial Nitric Oxide Synthase (NOS3) Haplotypes. Oxid Med Cell Longev. 2017;2017:2578950.

28.	Vidal K, Robinson N, Ives SJ. Exercise performance and physiological responses: the potential role of redox imbalance. Physiol Rep. 2017;5(7).

29.	Wiecek M, Maciejczyk M, Szymura J, Szygula Z. Sex differences in oxidative stress after eccentric and concentric exercise. Redox Rep. 2017;22(6):478-85.

30.	Boeno FP, Ramis TR, Farinha JB, de Lemos LS, Medeiros NDS, Ribeiro JL. Acute effects of strength exercise with blood flow restriction on vascular function of young healthy males. J Vasc Bras. 2018;17(2):122-7.

31.	Cangemi R, Loffredo L, Battaglia S, Perri L, Santulli M, Carnevale R, et al. Does Regular Physical Exercise Preserve Artery Dilation by Lowering Nox2-Related Oxidative Stress? Antioxid Redox Signal. 2018;28(17):1576-81.

32.	Cavarretta E, Peruzzi M, Del Vescovo R, Di Pilla F, Gobbi G, Serdoz A, et al. Dark Chocolate Intake Positively Modulates Redox Status and Markers of Muscular Damage in Elite Football Athletes: A Randomized Controlled Study. Oxid Med Cell Longev. 2018;2018:4061901.

33.	Georgakouli K, Fatouros IG, Fragkos A, Tzatzakis T, Deli CK, Papanikolaou K, et al. Exercise and Redox Status Responses Following Alpha-Lipoic Acid Supplementation in G6PD Deficient Individuals. Antioxidants (Basel). 2018;7(11).

34.	Hajizadeh Maleki B, Tartibian B, Mooren FC, FitzGerald LZ, Krüger K, Chehrazi M, et al. Low-to-moderate intensity aerobic exercise training modulates irritable bowel syndrome through antioxidative and inflammatory mechanisms in women: Results of a randomized controlled trial. Cytokine. 2018;102:18-25.

35.	Holloway GP, Holwerda AM, Miotto PM, Dirks ML, Verdijk LB, van Loon LJC. Age-Associated Impairments in Mitochondrial ADP Sensitivity Contribute to Redox Stress in Senescent Human Skeletal Muscle. Cell Rep. 2018;22(11):2837-48.

36.	Jamurtas AZ, Fatouros IG, Deli CK, Georgakouli K, Poulios A, Draganidis D, et al. The Effects of Acute Low-Volume HIIT and Aerobic Exercise on Leukocyte Count and Redox Status. J Sports Sci Med. 2018;17(3):501-8.

37.	Parker L, Trewin A, Levinger I, Shaw CS, Stepto NK. Exercise-intensity dependent alterations in plasma redox status do not reflect skeletal muscle redox-sensitive protein signaling. J Sci Med Sport. 2018;21(4):416-21.

38.	Sielski Ł, Sutkowy P, Skopowska A, Pawlak-Osińska K, Augustyńska Z, Hewelt K, et al. The Oxidant-Antioxidant Equilibrium and Inflammatory Process Indicators after an Exercise Test on the AlterG Antigravity Treadmill in Young Amateur Female Athletes. Oxid Med Cell Longev. 2018;2018:3484159.

39.	Trewin AJ, Parker L, Shaw CS, Hiam DS, Garnham A, Levinger I, et al. Acute HIIE elicits similar changes in human skeletal muscle mitochondrial H2O2 release, respiration, and cell signaling as endurance exercise even with less work. American Journal of Physiology-Regulatory, Integrative and Comparative Physiology. 2018;315(5):R1003-R16.

40.	Wadley AJ, Holliday A, Morgan RG, Heesom KJ, Aldred S, Peters DM, et al. Preliminary evidence of reductive stress in human cytotoxic T cells following exercise. J Appl Physiol (1985). 2018;125(2):586-95.

41.	Cumpstey AF, Minnion M, Fernandez BO, Mikus-Lelinska M, Mitchell K, Martin DS, et al. Pushing arterial-venous plasma biomarkers to new heights: A model for personalised redox metabolomics? Redox Biol. 2019;21:101113.

42.	De la Rosa A, Solana E, Corpas R, Bartrés-Faz D, Pallàs M, Vina J, et al. Long-term exercise training improves memory in middle-aged men and modulates peripheral levels of BDNF and Cathepsin B. Sci Rep. 2019;9(1):3337.

43.	de Oliveira Ottone V, Costa KB, Tossige-Gomes R, de Matos MA, Brito-Melo G, Magalhaes FC, et al. Late Neutrophil Priming Following a Single Session of High-intensity Interval Exercise. Int J Sports Med. 2019;40(3):171-9.

44.	Debevec T, Pialoux V, Millet GP, Martin A, Mramor M, Osredkar D. Exercise Overrides Blunted Hypoxic Ventilatory Response in Prematurely Born Men. Front Physiol. 2019;10:437.

45.	Kirkman DL, Ramick MG, Muth BJ, Stock JM, Pohlig RT, Townsend RR, et al. Effects of aerobic exercise on vascular function in nondialysis chronic kidney disease: a randomized controlled trial. Am J Physiol Renal Physiol. 2019;316(5):F898-f905.

46.	Margaritelis NV, Theodorou AA, Kyparos A, Nikolaidis MG, Paschalis V. Effect of body composition on redox homeostasis at rest and in response to exercise: The case of underfat women. J Sports Sci. 2019;37(14):1630-7.

47.	Oliveira VHF, Rosa FT, Wiechmann S, Narciso AMS, Webel AR, Franzói de Moraes SM, et al. Homocysteine-lowering exercise effect is greater in hyperhomocysteinemic people living with HIV: a randomized clinical trial. Appl Physiol Nutr Metab. 2019;44(11):1165-71.

48.	Petrick HL, Pignanelli C, Barbeau PA, Churchward-Venne TA, Dennis K, van Loon LJC, et al. Blood flow restricted resistance exercise and reductions in oxygen tension attenuate mitochondrial H(2) O(2) emission rates in human skeletal muscle. J Physiol. 2019;597(15):3985-97.

49.	Wadley AJ, Keane G, Cullen T, James L, Vautrinot J, Davies M, et al. Characterization of extracellular redox enzyme concentrations in response to exercise in humans. J Appl Physiol (1985). 2019;127(3):858-66.

50.	Alghadir AH, Gabr SA. Physical activity impact on motor development and oxidative stress biomarkers in school children with intellectual disability. Rev Assoc Med Bras (1992). 2020;66(5):600-6.

51.	Almeida EB, Santos JMB, Paixão V, Amaral JB, Foster R, Sperandio A, et al. L-Glutamine Supplementation Improves the Benefits of Combined-Exercise Training on Oral Redox Balance and Inflammatory Status in Elderly Individuals. Oxid Med Cell Longev. 2020;2020:2852181.

52.	Ashikawa H, Adachi T, Ueyama J, Yamada S. Association between redox state of human serum albumin and exercise capacity in older women: A cross-sectional study. Geriatr Gerontol Int. 2020;20(3):256-60.

53.	Ceci R, Duranti G, Di Filippo ES, Bondi D, Verratti V, Doria C, et al. Endurance training improves plasma superoxide dismutase activity in healthy elderly. Mech Ageing Dev. 2020;185:111190.

54.	Cho SY, So WY, Roh HT. Effect of C242T Polymorphism in the Gene Encoding the NAD(P)H Oxidase p22(phox) Subunit and Aerobic Fitness Levels on Redox State Biomarkers and DNA Damage Responses to Exhaustive Exercise: A Randomized Trial. Int J Environ Res Public Health. 2020;17(12).

55.	Dobashi S, Takeuchi K, Koyama K. Hydrogen-rich water suppresses the reduction in blood total antioxidant capacity induced by 3 consecutive days of severe exercise in physically active males. Med Gas Res. 2020;10(1):21-6.

56.	Goulart M, Pisamiglio DS, MÖller GB, Dani C, Alves FD, Bock PM, et al. Effects of grape juice consumption on muscle fatigue and oxidative stress in judo athletes: a randomized clinical trial. An Acad Bras Cienc. 2020;92(4):e20191551.

57.	Mooren FC, Maleki BH, Pilat C, Ringseis R, Eder K, Teschler M, et al. Effects of Escherichia coli strain Nissle 1917 on exercise-induced disruption of gastrointestinal integrity. Eur J Appl Physiol. 2020;120(7):1591-9.

58.	Ostrom EL, Traustadóttir T. Aerobic exercise training partially reverses the impairment of Nrf2 activation in older humans. Free Radic Biol Med. 2020;160:418-32.

59.	Rosa TS, Neves RVP, Deus LA, Sousa CV, da Silva Aguiar S, de Souza MK, et al. Sprint and endurance training in relation to redox balance, inflammatory status and biomarkers of aging in master athletes. Nitric Oxide. 2020;102:42-51.

60.	Sovatzidis A, Chatzinikolaou A, Fatouros IG, Panagoutsos S, Draganidis D, Nikolaidou E, et al. Intradialytic Cardiovascular Exercise Training Alters Redox Status, Reduces Inflammation and Improves Physical Performance in Patients with Chronic Kidney Disease. Antioxidants (Basel). 2020;9(9).

61.	Andrade-Lima A, Silva Junior N, Chehuen M, Miyasato R, Souza RWA, Leicht AS, et al. Walking Training Improves Systemic and Local Pathophysiological Processes in Intermittent Claudication. Eur J Vasc Endovasc Surg. 2021;61(6):954-63.

62.	Bellafiore M, Pintaudi AM, Thomas E, Tesoriere L, Bianco A, Cataldo A, et al. Redox and autonomic responses to acute exercise-post recovery following Opuntia ficus-indica juice intake in physically active women. J Int Soc Sports Nutr. 2021;18(1):43.

63.	Bosco G, Paganini M, Giacon TA, Oppio A, Vezzoli A, Dellanoce C, et al. Oxidative Stress and Inflammation, MicroRNA, and Hemoglobin Variations after Administration of Oxygen at Different Pressures and Concentrations: A Randomized Trial. Int J Environ Res Public Health. 2021;18(18).

64.	Corrêa HL, Neves RVP, Deus LA, Maia BCH, Maya AT, Tzanno-Martins C, et al. Low-load resistance training with blood flow restriction prevent renal function decline: The role of the redox balance, angiotensin 1-7 and vasopressin(✰,✰✰). Physiol Behav. 2021;230:113295.

65.	Corrêa HL, Deus LA, Neves RVP, Reis AL, de Freitas GS, de Araújo TB, et al. Influence of Angiotensin Converting Enzyme I/D Polymorphism on Hemodynamic and Antioxidant Response to Long-Term Intradialytic Resistance Training in Patients With Chronic Kidney Disease: A Randomized Controlled Trial. J Strength Cond Res. 2021;35(10):2902-9.

66.	de Farias JM, Dos Santos Tramontin N, Pereira EV, de Moraes GL, Furtado BG, Tietbohl LTW, et al. Physical Exercise Training Improves Judgment and Problem-Solving and Modulates Serum Biomarkers in Patients with Alzheimer's Disease. Mol Neurobiol. 2021;58(9):4217-25.

67.	Gaitán JM, Moon HY, Stremlau M, Dubal DB, Cook DB, Okonkwo OC, et al. Effects of Aerobic Exercise Training on Systemic Biomarkers and Cognition in Late Middle-Aged Adults at Risk for Alzheimer's Disease. Front Endocrinol (Lausanne). 2021;12:660181.

68.	Hureau TJ, Weavil JC, Sidhu SK, Thurston TS, Reese VR, Zhao J, et al. Ascorbate attenuates cycling exercise-induced neuromuscular fatigue but fails to improve exertional dyspnea and exercise tolerance in COPD. J Appl Physiol (1985). 2021;130(1):69-79.

69.	Koay YC, Stanton K, Kienzle V, Li M, Yang J, Celermajer DS, et al. Effect of chronic exercise in healthy young male adults: a metabolomic analysis. Cardiovasc Res. 2021;117(2):613-22.

70.	Lazar-Poloczek E, Romuk E, Rozentryt P, Duda S, Gąsior M, Wojciechowska C. Ceruloplasmin as Redox Marker Related to Heart Failure Severity. Int J Mol Sci. 2021;22(18).

71.	Leite-Almeida L, Morato M, Cosme D, Afonso J, Areias JC, Guerra A, et al. Impact of physical activity on redox status and nitric oxide bioavailability in nonoverweight and overweight/obese prepubertal children. Free Radic Biol Med. 2021;163:116-24.

72.	Mesquita PHC, Lamb DA, Godwin JS, Osburn SC, Ruple BA, Moore JH, et al. Effects of Resistance Training on the Redox Status of Skeletal Muscle in Older Adults. Antioxidants (Basel). 2021;10(3).

73.	Nascimento C, Peixoto MS, Fonte Boa LF, de Faria CC, Costa TSF, Matta L, et al. The Effects of Combined Physical Exercise on Serum Redox Biomarkers and Leukocyte DNA Damage of Obese Women. Oxid Med Cell Longev. 2021;2021:6638420.

74.	Quinn KM, Cox AJ, Roberts LA, Briskey D, Minahan C. Reliability of a point-of-care device to determine oxidative stress in whole blood before and after acute exercise: A practical approach for the applied sports sciences. J Sports Sci. 2021;39(6):673-82.

75.	Rosety-Rodriguez M, Bernardi M, Elosegui S, Rosety I, Diaz AJ, Rosety MA, et al. A Short-Term Resistance Training Circuit Improved Antioxidants in Sedentary Adults with Down Syndrome. Oxid Med Cell Longev. 2021;2021:8811153.

76.	Alvarez Villela M, Dunworth SA, Kraft BD, Harlan NP, Natoli MJ, Suliman HB, et al. Effects of high-intensity interval training with hyperbaric oxygen. Front Physiol. 2022;13:963799.

77.	Amaral AL, Batista JP, Mariano IM, Gonçalves LF, Tavares JB, de Souza AV, et al. Redox Status of Postmenopausal Women with Single or Multiple Cardiometabolic Diseases Has a Similar Response to Mat Pilates Training. Antioxidants (Basel). 2022;11(8).

78.	da Silva ML, de Sousa Neto IV, de Lima A, Barin F, de Toledo Nóbrega O, de Cássia Marqueti R, et al. Effects of Home-Based Electrical Stimulation on Plasma Cytokines Profile, Redox Biomarkers, and Metalloproteinases in the Heart Failure with Reduced Ejection Fraction: A Randomized Trial. J Cardiovasc Dev Dis. 2022;9(12).

79.	Estébanez B, Visavadiya NP, Vargas JE, Rivera-Viloria M, Khamoui AV, de Paz JA, et al. Resistance Training Modulates Reticulum Endoplasmic Stress, Independent of Oxidative and Inflammatory Responses, in Elderly People. Antioxidants (Basel). 2022;11(11).

80.	Grillet PE, Badiou S, Lambert K, Sutra T, Plawecki M, Raynaud de Mauverger E, et al. Biomarkers of Redox Balance Adjusted to Exercise Intensity as a Useful Tool to Identify Patients at Risk of Muscle Disease through Exercise Test. Nutrients. 2022;14(9).

81.	Hojan K, Gerreth K, Procyk D, Mania K, Zalewska A, Maciejczyk M. Redox Status Response of Physical Exercise Training in Women with Breast Cancer during Trastuzumab Therapy. Healthcare (Basel). 2022;10(10).

82.	Luti S, Militello R, Fiaschi T, Magherini F, Gamberi T, Parri M, et al. Preliminary results indicate that regular training induces high protection against oxidative stress in basketball players compared to soccer. Sci Rep. 2022;12(1):18526.

83.	Muggeridge DJ, Crabtree DR, Tuncay A, Megson IL, Davison G, Cobley JN. Exercise decreases PP2A-specific reversible thiol oxidation in human erythrocytes: Implications for redox biomarkers. Free Radic Biol Med. 2022;182:73-8.

84.	Pinto KMC, Diniz RCR, Tourino FD, de Lacerda LT, Bearzoti E, Costa KB, et al. Similar Inflammatory Adaptation in Women following 10 Weeks of Two Equalized Resistance Training with Different Muscle Action Duration. Biomed Res Int. 2022;2022:2185111.

85.	Póvoas S, Ascensão A, Magalhães J, Silva P, Wiig H, Raastad T, et al. Technical match actions and plasma stress markers in elite female football players during an official FIFA Tournament. Scand J Med Sci Sports. 2022;32 Suppl 1:127-39.

86.	Bacchetti T, Morresi C, Ferretti G, Larsson A, Åkerfeldt T, Svensson M. Effects of Seven Weeks of Combined Physical Training on High-Density Lipoprotein Functionality in Overweight/Obese Subjects. Metabolites. 2023;13(10).

87.	D'Amico A, Fossati C, Pigozzi F, Borrione P, Peruzzi M, Bartimoccia S, et al. Natural Activators of Autophagy Reduce Oxidative Stress and Muscle Injury Biomarkers in Endurance Athletes: A Pilot Study. Nutrients. 2023;15(2).

88.	Dos Santos JM, Taiar R, Ribeiro VGC, da Silva Lage VK, Scheidt Figueiredo PH, Costa HS, et al. Whole-Body Vibration Training on Oxidative Stress Markers, Irisin Levels, and Body Composition in Women with Fibromyalgia: A Randomized Controlled Trial. Bioengineering (Basel). 2023;10(2).

89.	Galvan-Alvarez V, Martin-Rincon M, Gallego-Selles A, Martínez Canton M, HamedChaman N, Gelabert-Rebato M, et al. Determinants of the maximal functional reserve during repeated supramaximal exercise by humans: The roles of Nrf2/Keap1, antioxidant proteins, muscle phenotype and oxygenation. Redox Biol. 2023;66:102859.

90.	Kim SY, Surh YJ, Lee YS. Effects of Exhaustive Exercise on Inflammatory, Apoptotic, and Antioxidative Signaling Pathways in Human Peripheral Blood Mononuclear Cells. J Cancer Prev. 2023;28(1):3-11.

91.	Lisi V, Moulton C, Fantini C, Grazioli E, Guidotti F, Sgrò P, et al. Steady-state redox status in circulating extracellular vesicles: A proof-of-principle study on the role of fitness level and short-term aerobic training in healthy young males. Free Radic Biol Med. 2023;204:266-75.

92.	Peeters WM, Gram M, Dias GJ, Vissers MCM, Hampton MB, Dickerhof N, et al. Changes to insulin sensitivity in glucose clearance systems and redox following dietary supplementation with a novel cysteine-rich protein: A pilot randomized controlled trial in humans with type-2 diabetes. Redox Biol. 2023;67:102918.

93.	Shadiow J, Miranda ER, Perkins RK, Mazo CE, Lin Z, Lewis KN, et al. Exercise-induced changes to the fiber type-specific redox state in human skeletal muscle are associated with aerobic capacity. J Appl Physiol (1985). 2023;135(3):508-18.

94.	Shinagawa A, Yamazaki T, Minematsu A, Serizawa N, Hosoi Y, Ninomiya Y, et al. Changes in homocysteine and non-mercaptoalbumin levels after acute exercise: a crossover study. BMC Sports Sci Med Rehabil. 2023;15(1):59.

95.	Thomas HJ, Ang T, Morrison DJ, Keske MA, Parker L. Acute exercise and high-glucose ingestion elicit dynamic and individualized responses in systemic markers of redox homeostasis. Front Immunol. 2023;14:1127088.

96.	Wouda MF, Slettahjell HB, Lundgaard E, Bastani NE, Raastad T, Blomhoff R, et al. Acute changes in antioxidants and oxidative stress to vigorous arm exercise: an intervention trial in persons with spinal cord injury and healthy controls. Spinal Cord Ser Cases. 2023;9(1):32.

97.	Jeppesen JS, Caldwell HG, Lossius LO, Melin AK, Gliemann L, Bangsbo J, et al. Low energy availability increases immune cell formation of reactive oxygen species and impairs exercise performance in female endurance athletes. Redox Biol. 2024;75:103250.

98.	Bourgonje AR, Knobbe TJ, Kremer D, Bulthuis MLC, Bemelman FJ, Berger SP, et al. Effect of lifestyle intervention on systemic oxidative stress in kidney transplant recipients: A post-hoc analysis of the Active Care after Transplantation (ACT) randomized controlled trial. Free Radic Biol Med. 2025;232:412-20.

99.	Chatzinikolaou PN, Margaritelis NV, Paschalis V, Theodorou AA, Moushi E, Vrabas IS, et al. Erythrocyte Glycolytic and Redox Metabolism Affects Muscle Oxygenation and Exercise Performance: A Randomized Double-Blind Crossover Study in Men. Sports Med. 2025.

100.	Pļaviņa L, Edelmers E. Oxidative Stress Modulation and Glutathione System Response During a 10-Day Multi-Stressor Field Training. J Funct Morphol Kinesiol. 2025;10(2).

101.	Sagini K, Selvarajah M, Romero S, Bassols-Citores E, Martin-Gracia B, Ramirez-Garrastacho M, et al. The Redox Enzyme Thioredoxin Is Increased in Plasma Extracellular Vesicles From Endurance-Trained Females in Response to Acute Exercise. Faseb j. 2025;39(16):e70927.

102.	Sim M, Song W, Choi EY, Shin DM, Kim CS. Chronic low water intake is associated with altered exercise-induced oxidative stress and immune cell responses: a cross-sectional study. J Int Soc Sports Nutr. 2025;22(1):2551213.

103.	Weber D, Thimm J, Seiz T, Kochlik B, Raupbach J, Bürkle A, et al. Association between redox biomarkers, DNA damage and aerobic capacity before and after physical stress in young men. Redox Biol. 2025;85:103764.

104.	Nørregaard LB, Hansen CC, Wickham KA, Møller S, Olsen K, Ehlers T, et al. Exercise training alters skeletal muscle microvascular endothelial cell properties in recent postmenopausal females. J Physiol. 2024;602(14):3449-68.

105.	Andriolo RB, El Dib RP, Ramos L, Atallah ÁN, da Silva EM. Aerobic exercise training programmes for improving physical and psychosocial health in adults with Down syndrome. Cochrane database of systematic reviews. 2010(5).

106.	de Sousa CV, Sales MM, Rosa TS, Lewis JE, de Andrade RV, Simoes HG. The antioxidant effect of exercise: a systematic review and meta-analysis. Sports medicine. 2017;47(2):277-93.

107.	Stigger FS, Zago Marcolino MA, Portela KM, Plentz RDM. Effects of exercise on inflammatory, oxidative, and neurotrophic biomarkers on cognitively impaired individuals diagnosed with dementia or mild cognitive impairment: a systematic review and meta-analysis. The Journals of Gerontology: Series A. 2019;74(5):616-24.

108.	Shields N, Downs J, de Haan JB, Taylor NF, Torr J, Fernhall B, et al. What effect does regular exercise have on oxidative stress in people with Down syndrome? A systematic review with meta-analyses. Journal of science and medicine in sport. 2018;21(6):596-603.

109.	Kim M, Eo H, Lim JG, Lim H, Lim Y. Can low-dose of dietary vitamin e supplementation reduce exercise-induced muscle damage and oxidative stress? A meta-analysis of randomized controlled trials. Nutrients. 2022;14(8):1599.

110.	Zhao M, Xiao M, Tan Q, Lyu J, Lu F. The effect of aerobic exercise on oxidative stress in patients with chronic kidney disease: a systematic review and meta-analysis with trial sequential analysis. Renal Failure. 2023;45(2):2252093.

111.	Ferlito JV, Rolnick N, Ferlito MV, De Marchi T, Deminice R, Salvador M. Acute effect of low-load resistance exercise with blood flow restriction on oxidative stress biomarkers: A systematic review and meta-analysis. Plos one. 2023;18(4):e0283237.

112.	Qiu C, Li S, Jia S, Yu F, Wei C, Wang X, et al. The effects of exercise on oxidative stress MDA and SOD in patients with type 2 diabetes: a systematic review and meta-analysis. PeerJ. 2025;13:e19814.

113.	Rosado-Pérez J, Castelan-Martinez OD, Mujica-Calderon AJ, Sanchez-Rodriguez MA, Mendoza-Nunez VM. Effect of tai chi on markers of oxidative stress: systematic review and meta-analysis. International journal of environmental research and public health. 2021;18(7):3458.

114.	Guedes LJL, Tavares VB, Carneiro SR, Neves LMT. The effect of physical activity on markers of oxidative and antioxidant stress in cancer patients: a systematic review and meta-analysis. BMC cancer. 2025;25(1):74.
